# Long-term electoral participation among Swedish survivors of the 2004 Indian Ocean tsunami

**DOI:** 10.1038/s41467-026-78593-8

**Published:** 2026-10-22

**Authors:** Kalle Ekholm, Lina M. Eriksson, Kåre Vernby

**Affiliations:** 1 Department of Government, Uppsala University, Uppsala, Sweden; 2 Department of Political Science, Linnaeus University, Växjö, Sweden; 3 Department of Political Science, Stockholm University, Stockholm, Sweden

**Keywords:** Politics, Natural hazards

## Abstract

Natural disasters can spark demands for democratic accountability yet impose losses and displacement that depress participation. We study approximately 16,000 Swedish tourists exposed to the 2004 Indian Ocean tsunami who returned home to intact communities. Unlike most people exposed to natural disasters, they faced no increase in the cost of voting, so the case is well suited to studying motivational responses to disaster exposure. We link a survivor registry to population-wide administrative data (*N* up to 7 million) and use entropy balancing to compare survivors to matched non-exposed citizens over 25 years. Exposed individuals do not withdraw from participation. Their turnout is 1.4 percentage points higher in 2010 and 2018, a modest but persistent gap. The gap is more than twice as large among the bereaved. Mobilization is strongest among those exposed between ages 16 and 25, which is what habit formation would predict. A 2023 follow-up survey (3,956 respondents; *N* = 3198 to 3625 per analysis after item non-response) provides descriptive evidence that survivors still evaluate the government’s crisis response negatively and have shifted their partisan sympathies away from the then-incumbent party. The findings indicate that failed crisis management may have lasting consequences for turnout and potentially party choice, and that the electoral stakes of disaster response extend beyond the disaster zone.

## Abstract (web summary)

It is unclear whether surviving a disaster makes people more or less likely to vote. Here, the authors show that Swedish survivors of the 2004 tsunami, who returned to intact homes, voted at higher rates for at least 14 years afterwards

## Introduction

In an era of rapid global warming, climatic extremes and natural disasters have become more frequent and destructive^1^. For example, the World Meteorological Organization reports a fivefold increase in weather-related disasters since 1970^2^, resulting in considerable economic damage and human suffering^3,4^. Investments in crisis preparedness and disaster management are therefore crucial to safeguard at-risk communities^5^. For democratic governments, such investments also carry political importance: leaders have an incentive to prepare for crises only if citizens remain engaged and able to hold them accountable at the polls^6^.

This raises the question of how experiencing a catastrophic disaster influences individuals’ willingness to participate in elections. Previous research suggests mixed expectations. Disasters often inflict personal and economic damage that can demobilize citizens. Displacement and resource loss raise the costs of voting and may discourage civic participation^7–9^. But direct exposure can also work in the opposite direction. Those exposed may want to hold governments accountable for failed crisis management and to demand support for recovery, which can raise participation^10–12^. It is therefore unclear whether the net long-run effect of disaster exposure is to mobilize or suppress electoral participation. Whichever force dominates in the short run, there are theoretical reasons to expect the initial impact on turnout to persist. According to previous research, voting is habit-forming, meaning that citizens who turn out in a given election are more likely to vote in subsequent ones. This self-reinforcing dynamic has been shown in experimental and quasi-experimental studies^13–15^. Moreover, research on political socialization suggests that experiences during the impressionable years (the teenage years and early adulthood) have longer-lasting effects on political behavior than experiences later in life^16,17^. If a disaster leads to an initial change in turnout, this change may persist because of habit formation^18^.

Whether disaster exposure ultimately mobilizes or suppresses electoral participation, and whether any such effect endures, remain open empirical questions. The 2004 Indian Ocean (Boxing Day) tsunami offers an opportunity to address them. It was one of the deadliest natural disasters in modern history, claiming over 225,000 lives and displacing more than a million people. Although the worst devastation occurred in Southeast Asia (with Indonesia, Sri Lanka, and Thailand hardest hit), the tsunami’s impact extended worldwide. Sweden suffered one of the largest losses of life outside the immediate region, as thousands of Swedish tourists were vacationing in Thailand when the tsunami struck^19^. Over 500 Swedish citizens lost their lives (roughly 7% of all casualties officially recorded in Thailand), and many more were injured or narrowly escaped.

The catastrophe became the most intensely covered news event in Sweden since World War II^20^, and the government’s perceived failure to assist its citizens quickly led to a major political accountability crisis. An independent commission was established to investigate the government’s handling of the disaster^19^, and high-profile hearings culminated in the resignation of a cabinet minister^21^. As one survivor put it at the time, “Sweden’s sheer inaction was almost worse than the disaster itself,” reflecting the frustration directed at the Swedish authorities’ handling of the crisis^22^. To this day, the tsunami remains a national trauma in Sweden, with survivors still publicly expressing disappointment in the government’s response^23^.

This setting lets us study the motivational effects of disaster exposure on political participation without the dislocation, resource constraints, and cost-of-voting increases that confound most existing studies. We focus on approximately 16,000 Swedish tourists who experienced the tsunami in the affected regions (primarily in Thailand). These individuals returned to a homeland that was not directly affected by the disaster, meaning that local voting infrastructure and institutions remained intact. Any change in their voting behavior therefore can not be explained by higher costs of voting.

To assess the tsunami’s impact on electoral participation, we use comprehensive individual-level data. We link official records of Swedish tsunami survivors^19^ with population-wide administrative registers from Statistics Sweden. These registers provide detailed demographic, socioeconomic, and voter turnout information for both the survivors and a control population. Because the study is observational, the central identification concern is that Swedes who vacationed in Southeast Asia may differ from those who did not on dimensions that also predict turnout trends. To address this, we use entropy balancing^24^, which aligns the control group with survivors on key characteristics that include pre-tsunami turnout, income, education, age, sex, and geographical region. We then track voter turnout for all these individuals in national elections before and after the tsunami, spanning a period of 25 years (*N* ranges from approximately 3,900,000 to 4,400,000 for each election included in the main analysis).

Here, we show that direct exposure to the tsunami is followed by an increase in long-term electoral participation. In our main entropy-balanced estimates, disaster exposure raises turnout by approximately 1.4 percentage points in both 2010 and 2018, a modest effect in absolute terms. However, it accounts for roughly 10% of the remaining non-voting margin given Sweden’s high baseline turnout of 85–87%. The estimate is virtually identical in the two elections. The increase is especially pronounced among the bereaved, who vote at rates approximately 5–6 percentage points above their entropy-balanced counterparts. It is also more pronounced among those who were aged 16–25 at the time of the disaster, consistent with the habit-formation account outlined above. Beyond turnout, a 2023 follow-up survey of over 3000 survivors and matched controls provides descriptive evidence that exposed respondents still evaluate the government’s crisis response negatively and are less likely to support the party that was in government at the time of the tsunami. While the survey cannot establish whether these patterns are causal, they are consistent with a lasting partisan shift in the affected population.

## Results

### Background

**Fig. 1:**
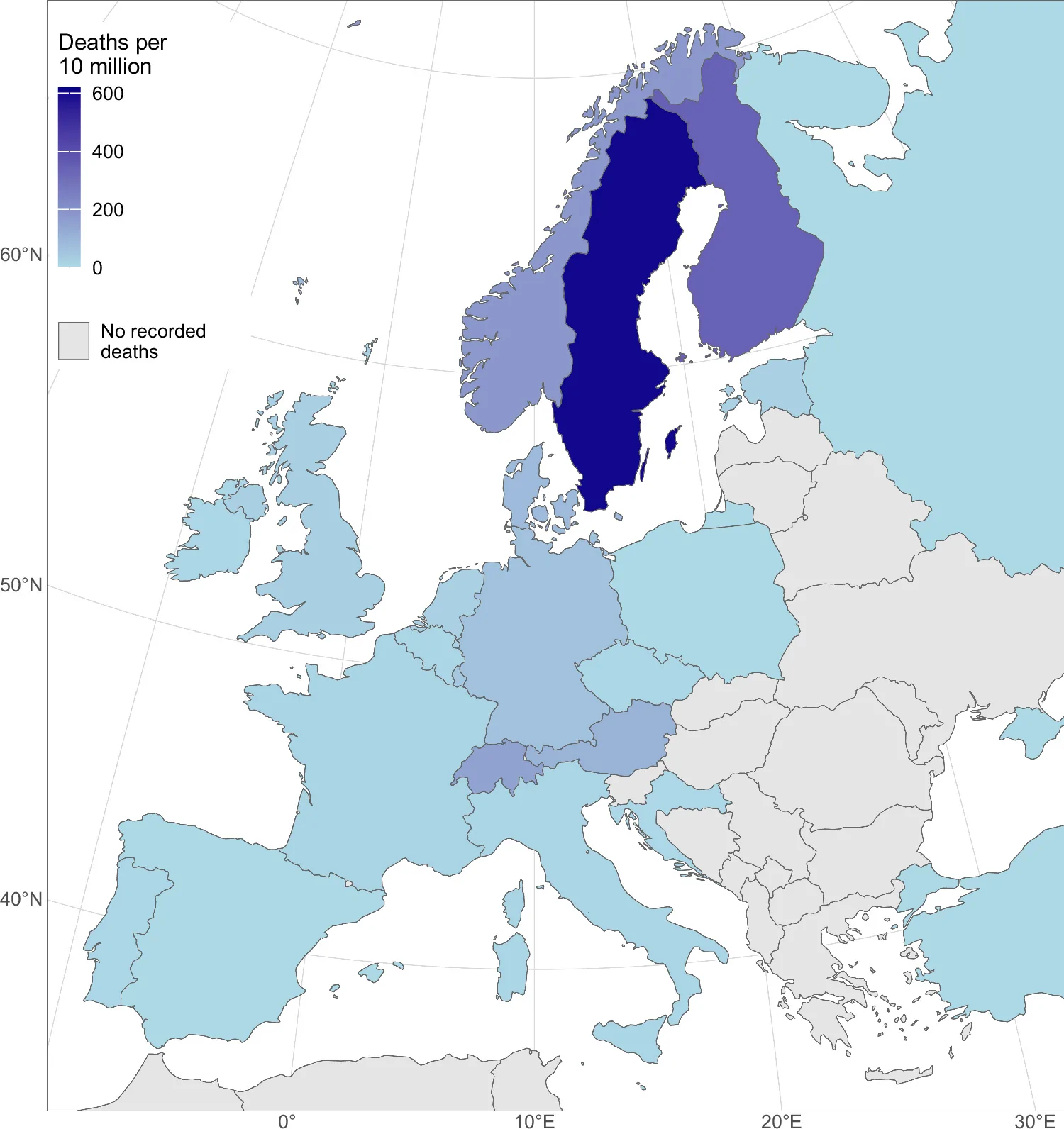
Tourist fatalities per 10 million inhabitants in mostly European countries after the 2004 Boxing Day tsunami. See Supplementary Table 14 for data sources. Source data are provided as a Source Data file.

We begin by documenting the tsunami’s scope and Sweden’s unexpectedly large share of casualties. At the time of the tsunami, Thailand and, to a lesser extent, Sri Lanka were popular winter destinations for vacationing Swedes. On December 26, 2004, the first waves of the tsunami hit the Thai shores at 9.59 a.m. local time. At this point, many tourists were already on the beach and were taken by surprise by the waves^19^. In total, about 600 Swedes were in Sri Lanka at the time of the tsunami, and about 15,500 were in Thailand^19^. The National Police Board, who were in charge of passport controls, registered 16,030 Swedes as tsunami-affected citizens upon their return home^19^. In terms of casualties, a total of 543 Swedes lost their lives. Out of these, 140 were children, and about 60 children lost at least one parent^25^. Figure 1 shows the number of casualties, where such statistics exist, for a group of mostly European countries. As can be seen, Sweden was the hardest-hit country in this region, and this conclusion becomes reinforced once one considers Sweden’s comparatively small population, which was about 9 million in 2004^26^.

How the Swedish state responded—or failed to respond—shaped survivors’ perceptions of governmental competence. The Swedes who lived through the tsunami expected a substantial rescue mission to reach Thailand and Sri Lanka within 14–18 h, but no such help arrived. When they sought assistance at Swedish embassies and consulates, staff referred them to private travel agencies and insurance companies instead. By contrast, the United States, Italy, Germany, Finland, and France promptly dispatched evacuation flights for their nationals, giving Swedish tourists a clear counterfactual of effective crisis response^19^. The resulting sense of discontent and betrayal has endured: “I still feel a great disappointment about how Sweden took care of its citizens. Shame on you. Thank you, Poland, for being there and flying us home,” one respondent wrote in our recent crisis-preparedness survey. Extensive 20-year anniversary coverage in 2024—replete with survivor interviews—confirms that dissatisfaction with the government’s handling of the tsunami remains widespread two decades later^23,27–32^.

Domestic pressure quickly translated into formal inquiries and electoral consequences. In late 2005, the Crisis Commission (Katastrofkommissionen), appointed to evaluate the authorities’ handling of the tsunami, delivered a report consisting of more than 1000 pages of investigations, pinpointing several structural weaknesses in the Swedish crisis response system. The report also contains around 350 stories from tsunami-affected Swedes, collected in the form of personal interviews and letters. From these stories, it is clear that Swedish tsunami victims expected a swift international crisis response by the Swedish government and that the impact of the tsunami on these Swedes was not peripheral. By the end of January 2006, the Committee on the Constitution began holding hearings, which revealed further mishandling. Shortly after, the Foreign Minister handed in their resignation^21^. In the election of September 2006, which was the first general election held following the tsunami, the Social Democrats were ousted from power, and previous research has argued that voter dissatisfaction with their handling of the tsunami played an important role^12^.

The accountability process stayed alive after the election, when it was revealed from some data tapes that the Crisis Commission and the Committee on the Constitution had received incorrect information during the review of the tsunami response. The new evidence made it clear that the cabinet committee was much less active than previously claimed^33^. This is vividly remembered by those who survived the tsunami. As one respondent wrote in our recent survey on societal crisis preparedness: “[T]he government handled the whole tsunami disaster poorly, and it is a complete scandal that they classified the so-called tsunami tapes twice.”

In events such as Hurricane Katrina^8,34^, residents face the destruction of their homes and communities alongside the political impulse to hold leaders accountable, making motivation and resource constraints hard to disentangle. The Swedish tourists returned to intact communities, so resource constraints of this kind were fewer. The case has additional advantages. Because catastrophes strike without warning, individual-level data from before the event rarely exist, and most disaster studies rely on aggregate measures for affected areas^9–12,35^. We link the tsunami registry to population-wide administrative registers and observe every exposed Swede, and a control group, in three elections before and after the disaster. This lets us estimate the tsunami’s effect on individual turnout directly. It also avoids a problem that affects place-based studies: selective out-migration in the wake of shocks, such as natural disasters^36^.

### Main findings

While there are several methodological advantages to studying the Swedish case, it remains an observational study. The central challenge for identification is selection into treatment. In our main analysis, we therefore rely on two complementary strategies to increase the comparability between the exposed and unexposed groups. First, we restrict the sample to individuals who appear in the 1994 turnout file and were thus eligible to vote before the disaster. This ensures that the composition of the exposed and unexposed groups is not affected by aging into eligibility or by post-tsunami migration over the course of the elections studied. Second, we apply entropy balancing^24^, which reweights the control group so that the means of all pre-treatment covariates match those of the treated group exactly. The covariates are prior turnout, income, education, age, age squared, sex, and region indicators. The ability to condition on prior voting is important because it serves as a summary measure of the latent political engagement that observable demographics alone may not fully capture.

Table 1 reports covariate means before and after entropy balancing for the two election-year samples. As expected, imbalances exist prior to re-weighting: tsunami survivors are on average six years younger, considerably more educated, and earn more than the control population. After entropy balancing, all differences are effectively eliminated—including on pre-tsunami turnout, where the two groups were already close but are now indistinguishable (detailed balance statistics, including covariate and regional balance assessments, as well as complete entropy–weight diagnostics, are provided in Supplementary Tables 1–5 and Supplementary Notes 1, 2).

**Table 1:** Covariate balance before and after entropy balancing

	Before EB			After EB		
Covariate	Treated	Control	Diff	Treated	Control	Diff
Panel A: 1994–2010						
Sex (female)	0.461	0.512	−0.051	0.461	0.461	0.000
Income (log)	7.728	7.497	0.231	7.728	7.728	0.000
Education	0.363	0.249	0.114	0.363	0.363	0.000
Age (years)	51.860	58.074	−6.214	51.860	51.870	−0.010
Age^2^	2804.656	3597.671	−793.015	2804.656	2805.966	−1.311
Turnout 1994 (%)	89.223	89.164	0.059	89.223	89.225	−0.002
Panel B: 1994–2018						
Sex (female)	0.461	0.511	−0.049	0.461	0.461	0.000
Income (log)	7.734	7.547	0.187	7.734	7.734	0.000
Education	0.368	0.266	0.102	0.368	0.368	0.000
Age (years)	59.341	63.037	−3.696	59.341	59.342	−0.001
Age^2^	3628.173	4144.461	−516.288	3628.173	3628.245	−0.072
Turnout 1994 (%)	89.236	89.064	0.173	89.236	89.236	0.000

The table reports covariate means for tsunami-exposed individuals (Treated) and unexposed controls (Control) before and after entropy balancing (EB). The sample is restricted to individuals who appear in the 1994 turnout file and were thus eligible to vote before the disaster. Before EB, columns show unweighted means; notable imbalances exist on income, education, and age. After entropy balancing, the control group is reweighted so that the means of all pre-treatment covariates match those of the treated group exactly; all differences are effectively eliminated. Panels A and B differ because the underlying populations change slightly across election years due to mortality and emigration. Turnout is rescaled to percentage points (0–100). All covariates are measured in 2003, prior to the tsunami. The entropy balancing also conditions on regions; full balance tables including regional indicators are reported in Supplementary Tables 1 and 2.

Overall, our results provide clear evidence that exposure to the 2004 Boxing Day tsunami did not reduce voter turnout. Instead, the results in Table 2 show a persistent increase in participation. Using the restricted sample with entropy-balanced weights, we estimate a tsunami-exposure effect on turnout of 1.444 percentage points in 2010 (*t*(4,374,651) = 4.98, *p* < 0.001, 95% CI = [0.876, 2.011]) and 1.442 percentage points in 2018 (*t*(3,880,130) = 5.45, *p* < 0.001, 95% CI = [0.924, 1.961]) (full results in Supplementary Table 6). An alternative way of adjusting for stable individual-level characteristics, entropy-balanced difference-in-differences, yields nearly identical point estimates (Supplementary Table 9). The effect is modest in absolute terms, though it accounts for roughly 10% of the remaining non-voting margin given Sweden’s baseline turnout of 85–87%. More striking than the magnitude is the persistence, with the effect spanning a period of 14 years after the disaster. This absence of decay is consistent with Fujiwara et al.^15^, who estimate that a one-point turnout shock persists at 0.6–1.0 points in the next election, with confidence intervals including full persistence.

**Table 2:** Effect of tsunami exposure on voter turnout, 2010 and 2018

	Estimate	95% CI	*t*	*p*
2010 election				
Tsunami exposure	1.444	0.876–2.011	4.98	<0.001
2018 election				
Tsunami exposure	1.442	0.924–1.961	5.45	<0.001
	2010	2018		
*N*, total	4,374,679	3,880,158		
*N*, exposed	7674	7646		
*N*, unexposed	4,367,005	3,872,512		
Adjusted *R*^2^	0.122	0.088		

Linear probability models estimated by weighted least squares with entropy-balancing weights and heteroskedasticity-robust standard errors. Estimates are the difference in turnout between exposed and unexposed individuals in percentage points (0–100). CI, confidence interval. t- statistics and *p*-values are from two-sided t-tests of the null hypothesis that the estimate equals zero; no adjustment for multiple comparisons was made. All models include the balancing covariates (sex, log income, education, age, age squared, all measured in 2003), turnout in 1994, and region fixed effects; full results are reported in Supplementary Table 6. Sample: restricted (see “Methods”).

The turnout effect is also larger among prior non-voters and lower-income individuals (Supplementary Figs. 1 and 2), so the mobilization was not concentrated among the core voters who typify the exposed population^37^. These analyses use the same restricted sample and entropy-balanced design as Table 2.

Even after entropy balancing, unobserved confounders cannot be ruled out in an observational study. Although traveling abroad is not statistically significantly associated with higher turnout in national survey data (Supplementary Table 13), administrative records cannot capture traits (e.g., cosmopolitanism or institutional trust) that may predict overseas travel and political engagement. We address this residual concern in two ways.

First, we report an informal sensitivity exercise using the standard omitted variable bias formula. Formal frameworks, such as Cinelli & Hazlett^38^, are not applicable here, since entropy balancing makes observed covariates uncorrelated to treatment by construction, and thus leaves no reference point for benchmarking. Anchoring the comparison in education, a canonical predictor of turnout, in 2010 education is associated with a 3.847 percentage-point increase in turnout in the entropy-balanced sample (*t*(4,374,651) = 14.40, *p* < 0.001, 95% CI = [3.323, 4.370]), an unobserved confounder of the same magnitude would need to raise the probability of tsunami exposure by 37 percentage points (1.4/3.8 ≈ 0.37) to eliminate the effect entirely. A confounder of this strength, operating net of an already equalized covariate distribution, is difficult to sustain empirically.

The benchmarking exercise shows that an unobserved confounder would need to be unusually strong to account for the main effect. Our second approach compares survivors with one another, all of whom share whatever traits led them to travel to the region at the time. Not every Swede present in Thailand or Sri Lanka was equally affected by the tsunami. The survivor registry lets us identify a subgroup that suffered the greatest loss: those who lost an immediate family member. We link survivor and fatality lists to Sweden’s multigenerational register and identify individuals who lost a parent, child, partner, sibling, or grandparent in the disaster. We then separate the exposed population into two mutually exclusive groups, bereaved and non-bereaved survivors, and compare them to each other and to unexposed controls.

Comparing bereaved and non-bereaved survivors is useful because traits, such as cosmopolitanism or institutional trust, that may be associated with selecting into overseas travel are shared by all tsunami-exposed individuals. By restricting the comparison to bereaved and non-bereaved survivors, the impact of such traits is netted out. If the turnout effect is a consequence of the severity of disaster exposure rather than pre-existing characteristics of travelers, we should observe larger effects among the bereaved. By contrast, the two groups should look similar if the turnout effect comes from traits associated with travel.

Bereavement clusters within families, and in many cases the children in such families were not yet eligible to vote in 1994. Therefore, conditioning on presence in the 1994 turnout file would eliminate nearly 40% of the already small bereaved subsample. We therefore present this analysis on the unrestricted sample. Table 3 reports the results. The main finding in Table 3 is that the effect of exposure is considerably stronger for the bereaved group than for the non-bereaved group. Non-bereaved survivors show a turnout effect of 2.375 percentage points in 2010 (*t*(6,701,908) = 8.87, *p* < 0.001, 95% CI = [1.850, 2.900]) and 2.095 points in 2018 (*t*(7,038,740) = 9.55, *p* < 0.001, 95% CI = [1.665, 2.525]). For bereaved survivors, the effect is 5.757 points in 2010 (*t*(6,701,908) = 3.53, *p* < 0.001, 95% CI = [2.561, 8.953]) and 4.940 points in 2018 (*t*(7,038,740) = 4.10, *p* < 0.001, 95% CI = [2.578, 7.301]). The difference between bereaved and non-bereaved survivors is statistically significant in both elections: 3.382 points in 2010 (*t*(6,701,908) = 2.05, *p* = 0.041, 95% CI =  [0.145, 6.619]) and 2.845 points in 2018 (*t*(7,038,740) = 2.32, *p* = 0.020, 95% CI = [0.446, 5.243]). According to these results, those who suffered the greatest loss were also the most mobilized to vote. This is the pattern we would expect if the turnout effect comes from the disaster experience itself, and it is hard to explain if the effect comes from traits shared by everyone who traveled.

**Table 3:** Effect of tsunami exposure and bereavement on voter turnout, 2010 and 2018

	Estimate	95% CI	*t*	*p*
2010 election				
Exposed, not bereaved	2.375	1.850–2.900	8.87	<0.001
Exposed and bereaved	5.757	2.561–8.953	3.53	<0.001
Difference, bereaved vs. not	3.382	0.145–6.619	2.05	0.041
2018 election				
Exposed, not bereaved	2.095	1.665–2.525	9.55	<0.001
Exposed and bereaved	4.940	2.578–7.301	4.10	<0.001
Difference, bereaved vs. not	2.845	0.446–5.243	2.32	0.020
	2010	2018		
*N*, total	6,701,936	7,038,768		
*N*, exposed and bereaved	241	297		
*N*, exposed, not bereaved	12,648	14,371		
*N*, unexposed	6,689,047	7,024,100		
Adjusted *R*^2^	0.033	0.022		

Linear probability models estimated by weighted least squares with entropy-balancing weights and heteroskedasticity-robust standard errors. Estimates are differences in turnout relative to unexposed individuals in percentage points (0–100); the difference rows test whether the bereaved and non-bereaved estimates are equal. CI, confidence interval. t statistics and *p*-values are from two-sided t-tests; no adjustment for multiple comparisons was made. All models include the balancing covariates (sex, log income, education, age, and age squared, all measured in 2003) and region fixed effects. Sample: unrestricted (see “Methods”).

The analyses presented here are consistent with a causal interpretation of the effect of tsunami exposure on turnout. Given the observational nature of our study, selection on unobservables cannot be definitively ruled out. However, the strategies presented above, each addressing the threat from a different angle, all speak against a pure selection account.

### Differential effects across age-cohorts

The previous section reveals a striking finding: the mobilization effect persists for well over a decade, which is difficult to reconcile with standard models of retrospective voting that assume a myopic electorate. One explanation for this persistence draws on a well-established regularity in the study of political behavior: voting is habit-forming. Experimental and quasi-experimental research has shown that citizens who turn out in a given election are more likely to vote in the elections that follow^13–15^. A related literature on political socialization suggests that experiences during the so-called impressionable years (often defined as spanning ages 16–25) have an especially strong effect on later-life political attitudes and behavior^16,17^. If tsunami exposure led to an increase in turnout, and if that increase was particularly large among young adults in their impressionable years, habit formation could then sustain the effect long after the initial increase^18^.

To test for this pattern, we estimate an entropy-balanced model in which tsunami exposure is interacted with age-group indicators. We distinguish between four cohorts, which are defined by the age of the exposed at the time of the disaster: 15 or under, 16–25 (the impressionable-years cohort), 26–50, and over 50. Those who were 15 or younger act as the reference category. This analysis requires the unrestricted sample: in the restricted sample, the youngest individual eligible to vote in September 1994 would have been at least 28 at the time of exposure, placing the entire impressionable-years cohort outside the estimation window. Because the youngest cohorts were not yet adults in 2003, their income and education are imputed from parental records. While this imprecision affects exposed and unexposed individuals alike, our results should nevertheless be interpreted with this caveat in mind.

**Fig. 2:**
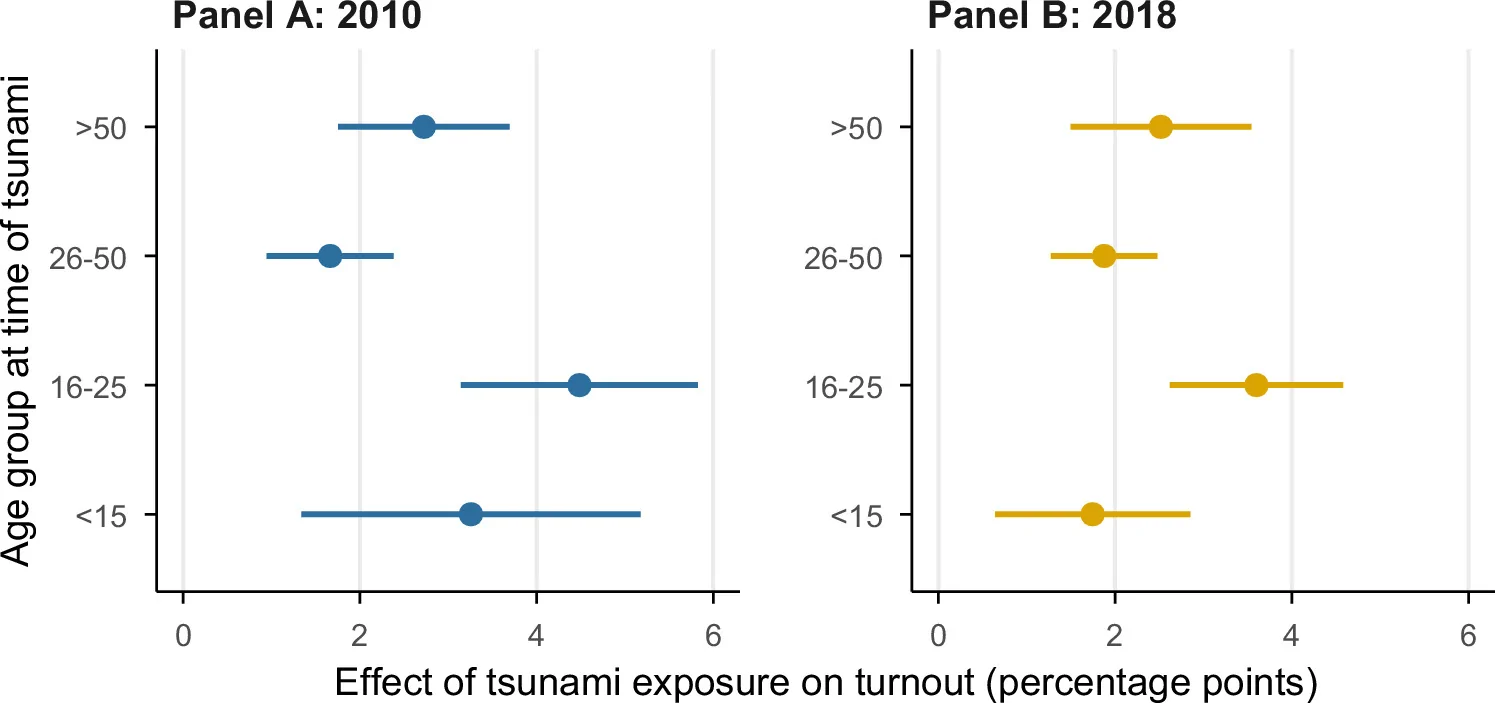
Average marginal effect of tsunami exposure on voter turnout, by age at the time of the tsunami. Points represent average marginal effects of tsunami exposure on voter turnout (percentage points) in 2010 (**A**) and 2018 (**B**), Average marginal effects are estimated separately by age group at the time of the tsunami (measured in 2004: ≤15, 16–25, 26–50, and >50 years), from entropy-balanced linear probability models including region fixed effects and covariates (sex, log income, education, and age^2^). Error bars represent 95% confidence intervals; robust standard errors. Sample: unrestricted (see “Methods”); *N* = 6,701,936 (**A**, 2010) and *N* = 7,038,768 (**B**, 2018). *P* values are two-sided, not adjusted for multiple comparisons, and reported exactly in Supplementary Table 12, which also reports the full underlying model. Source data are provided as a Source Data file.

Figure 2 plots the marginal effect of tsunami exposure on turnout by age group for the 2010 and 2018 elections (the full interaction model is in Supplementary Table 12). In both elections, the largest exposure effects appear among those who were 16–25 at the time of the disaster. In 2010, the effect for this group is 4.5 percentage points (*t*(6,701,904) = 6.55, *p* < 0.001, 95% CI = [3.1, 5.8]), compared to 3.3 for those 15 or under (*t*(6,701,904) = 3.32, *p* < 0.001, 95% CI = [1.3, 5.2]), 1.7 for those aged 26–50 (*t*(6,701,904) = 4.54, *p* < 0.001, 95% CI = [0.9, 2.4]), and 2.7 for those over 50 (*t*(6,701,904) = 5.50, *p* < 0.001, 95% CI = [1.8, 3.7]). In 2018, the effect is also strongest (3.6 percentage points, *t*(7,038,736) = 7.20, *p* < 0.001, 95% CI = [2.6, 4.6]) for the 16–25 cohort. Those who were children at the time of the tsunami (the reference group) also show sizeable effects, despite not yet being politically active when the disaster struck. Exposure at an early age may thus have affected their political behavior later in adulthood.

The results in this section suggest that the tsunami’s mobilizing effect was strongest on age-cohorts that were affected during the period when political habits are most malleable, and that the initial increase translated into a durable increase in participation that remained detectable more than a decade later.

### Did the government’s crisis response trigger a lasting realignment?

The turnout results provide strong evidence that exposure to the tsunami durably mobilized survivors. Did it also change whom they voted for? Key^39^ argued that certain elections produce realignments that persist for extended periods of time. His focus was on system-wide shifts, but recent work suggests that the same logic may apply to narrower groups in response to failed crisis management. Cremaschi et al.^40^ document a lasting electoral shift in Italian communities that felt abandoned by the state after a plant epidemic, while Bechtel et al.^10^ show that competent disaster relief can generate durable electoral rewards. We ask whether the Swedish tsunami produced a shift of the first kind.

Previous research on the Swedish case, relying on aggregate municipality-level exposure and vote shares, has shown that the Social Democrats were punished in the 2006 election, the first general election after the tsunami^12^. To explore whether this initial shift persisted among survivors or faded as the event receded from public attention, we fielded a survey in 2023 on attitudes toward societal crisis preparedness. The survey was sent to 5000 survivors drawn from the tsunami registry and 5000 individuals from a matched control group (matched on income, education, age, sex, and geographical region), and was administered by Statistics Sweden. The overall response rate was 40 percent, yielding over 3000 respondents split approximately equally between the two groups. The questionnaire covered attitudes about crisis preparedness as well as vote choices (see “Methods” for details on the survey design and sampling strategy). Since the survey data are cross-sectional and collected nearly two decades after the event, the patterns reported below are descriptive.

**Fig. 3:**
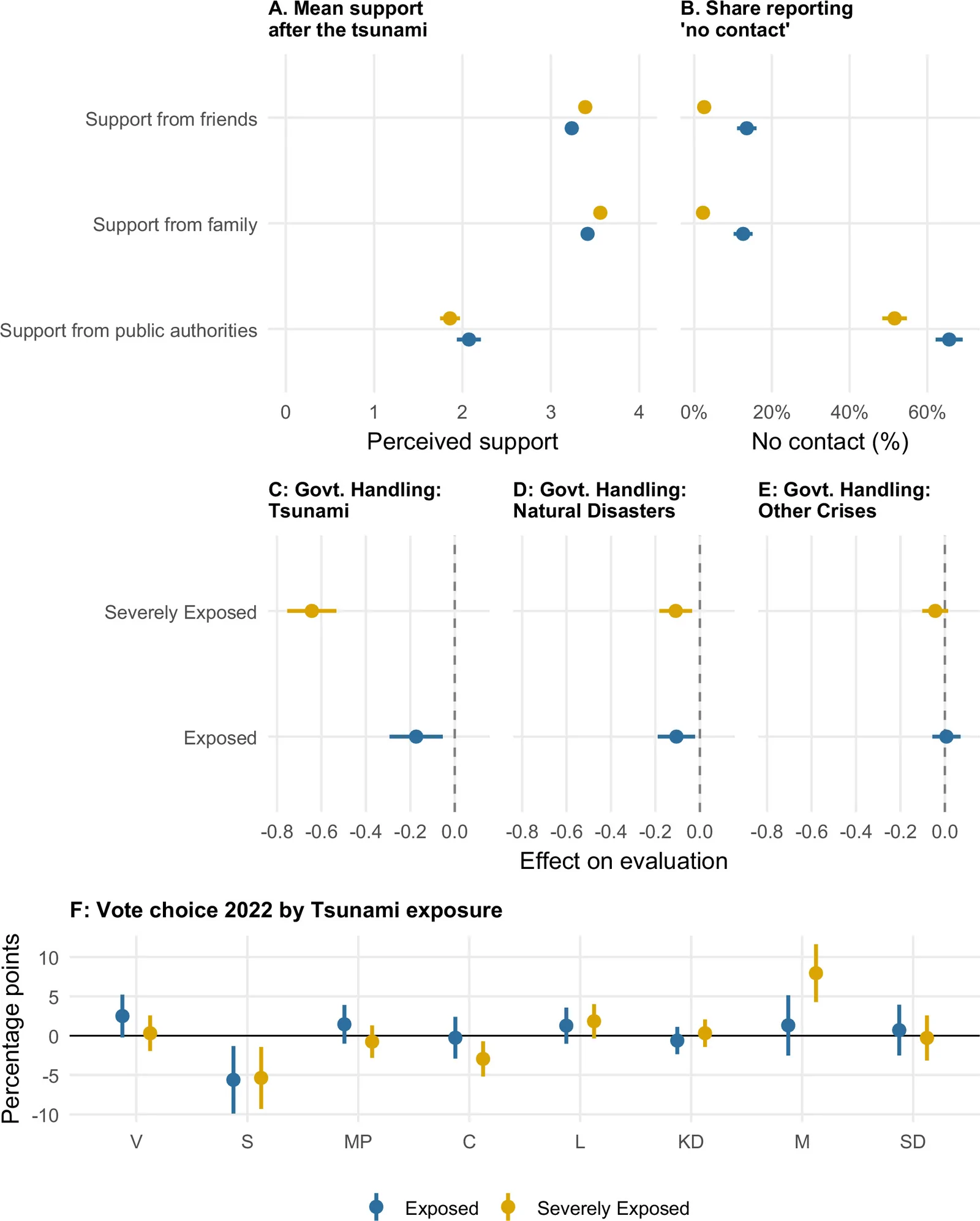
Social support, survivor evaluations of government crisis response and partisanship: survey evidence. **A** Points are the mean level of perceived support from three sources (0 = very poor, 4 = very good), restricted to exposed respondents who reported contact with the respective support source after the disaster (*n* = 631 and 947 for family, 626 and 946 for friends, and 247 and 468 for authorities; exposed and severely exposed, respectively). **B** Points are the share of exposed respondents reporting no contact with the respective support source after the disaster (*n* = 722 and 969 for family, 724 and 971 for friends, and 719 and 967 for authorities; exposed and severely exposed, respectively). Response distributions for **A**, **B** are shown in Supplementary Fig. 5 (Supplementary Note 3). Points are OLS coefficients for the association between exposure and evaluations of the government’s handling of **C** the 2004 tsunami, **D** natural disasters in general, and **E** other crises (0 = very poor, 4 = very good), controlling for age, sex, education, and income. The reference category is non-exposed controls (*n* = 3257, 3221, and 3625 for **C**–**E**, respectively). Full regression results are reported in Supplementary Note 3. **F** Points are OLS coefficients for the association between exposure and self-reported vote choice in the 2022 general election (percentage points), controlling for age, sex, education, and income (*n* = 3198). Full regression results are reported in Supplementary Note 3. Party abbreviations with party families: V, Left Party (socialist); S, Social Democrats (social democratic; the incumbent party in 2004); MP, Green Party (ecological); C, Centre Party (agrarian); L, Liberals (liberal); M, Moderate Party (conservative; the main opposition party in 2004); KD, Christian Democrats (Christian democratic); SD, Sweden Democrats (nationalist). In all panels, blue markers denote exposed respondents and orange markers denote severely exposed respondents. In all panels, bars represent 95% confidence intervals. Source data are provided as a Source Data file.

The survey evidence suggests that the experience of governmental failure among the tsunami-affected is linked to contemporary partisan evaluations. Panels A and B of Fig. 3 describe what survivors received from public authorities after the disaster. Most exposed respondents report no contact with authorities at all, and even among the severely exposed the share is around half (Panel B). Those who did have contact rate the support as no better than neutral, considerably below the ratings they give to family and friends (Panel A).

Panels C–E show that exposed respondents continue to rate the government’s handling of the tsunami significantly worse than non-exposed respondents, and that evaluations are most negative among the severely exposed. By contrast, no statistically significant difference is found when we turn to crisis management in general (Panel E). The association is thus statistically significant for evaluations of the 2004 response but not for crisis management in general. This pattern parallels Bechtel & Mannino^41^, who do not find a statistically significant link between disaster exposure and broad support for preparedness investment. The severely exposed also report significantly lower trust in politicians’ and authorities’ capacity to manage future crises, while there is no statistically significant difference in trust in ordinary people (full results in Supplementary Note 3).

Panel F turns to partisanship and shows that exposed respondents are less likely to support the then-incumbent Social Democrats and more likely to support the Moderates, which were the main opposition party at the time of the crisis. Again, gaps are larger for the severely exposed. No other party shows shifts of comparable size.

These patterns are consistent with a lasting realignment: survivors still evaluate the government’s tsunami response negatively, and these evaluations coincide with a lasting shift in party sympathies. The survey is descriptive and cannot establish that the experience caused the shift. But the grievance is specific; it is strongest among the most exposed, and the results align with the aggregate-level findings of Eriksson^12^.

## Discussion

In an era of increasingly frequent and severe climatic extremes and natural disasters^1^, scholars are paying closer attention to the repercussions of such events on political behavior. Yet most existing studies are based on aggregate data^9–12,35^, which makes it difficult to separate individual-level effects from ecological effects. In addition, they are set mainly in contexts where those most affected by a disaster also face the steepest increases in the cost of voting, complicating efforts to study the motivational impact of exposure on participation.

Using high-resolution population-wide Swedish registry data, we show that exposure to the 2004 Boxing Day tsunami was followed by a lasting increase in voting propensity, an effect that is more pronounced among the bereaved. The persistence of this effect, and its concentration among those exposed during their impressionable years, are consistent with habit formation as a sustaining mechanism. Descriptive survey evidence further suggests that the initial accountability response may have reshaped not only whether survivors vote but whom they vote for.

Because Swedish tourists returned home to intact houses, neighborhoods, and labor markets, the turnout increase cannot be explained by the higher costs of voting that follow most disasters. Our results are therefore consistent with a motivational response to the disaster. However, we cannot rule out the possibility that trauma also affected participation through indirect channels—such as long-term mental health impacts, family disruption, or subtle economic strain—that do not fit neatly into either a motivation or a resources category. Still, the positive direction of the effect weighs against explanations emphasizing the demobilizing consequences of trauma. If mental health deterioration or family disruption were the dominant pathway, we would expect turnout to fall.

How generalizable are our findings? Our design is optimized for internal validity, not external validity. Most disasters analyzed in the literature, like Hurricane Katrina, temporarily raise the cost of voting due to destruction and displacement. Our estimates therefore capture the component of the overall effect that remains when voting costs are held constant. We do not claim that the magnitude of our estimates would transfer directly to other settings. However, our findings suggest that the positive response we document may be latent in other disaster-affected populations. If so, measures that reduce the cost of voting in the aftermath of disasters, such as flexible registration, expanded early voting, or temporary polling stations, would not merely restore baseline participation but could also lead to the kind of durable mobilization we observe in our study.

The findings are encouraging for democratic accountability. While one should not downplay the severity of the psychological and material costs incurred by the victims of natural disasters and poor crisis management by responsible governments, our results do suggest that affected individuals do not necessarily become politically apathetic as a result. More broadly, the finding that survivors were mobilized rather than demobilized resonates with research on post-traumatic growth, which suggests that adversity can under certain conditions strengthen personal agency and civic engagement ^42,43^. For democratic governments, this means that there is an incentive to invest in crisis preparedness and management. Moreover, this incentive extends beyond national borders as governments may be held to account for their response to disasters that affect their citizens abroad.

## Methods

### Research ethics

This research was reviewed and approved by the Swedish Ethical Review Authority (see approval numbers 2022-02590-01 and 2022-04528-01). We combine several administrative registers containing sensitive personal information. The most sensitive source is the Swedish National Police Board’s tsunami register, which lists personal identity numbers, names, and postal addresses of individuals who survived or perished in the tsunami. Statistics Sweden (SCB) performed all linkages between this register and additional administrative data (from the National Board of Health and Welfare, the SCB population register, and turnout data from ref. ^44^). SCB then delivered a pseudonymized dataset to the research team in which all identifiers were replaced by serial numbers. Consequently, no individual can be re-identified in our analyses, and individual consent was not required under Swedish law for this type of register-based research. We also fielded a survey to a subset of individuals in the dataset. This survey was administered by Statistics Sweden, and respondents were informed of the survey’s subject matter prior to providing informed consent. This data was also delivered in pseudonymized form to the researchers.

### Registry data

To examine if the 2004 Boxing Day tsunami had an effect on exposed Swedes’ voter turnout, our analysis relies on two complementary sources of data. To identify those who were exposed, we rely on the Tsunami Registry, which was created by the Swedish National Police Board when screening passports of returning Swedes. The registry was created to account for every Swedish citizen who was evacuated from the areas that were devastated by the 2004 Boxing Day Tsunami. In total, 16,030 living individuals were evacuated, and 530 were identified as deceased^19^. We combine this data with data from various administrative sources maintained by Statistics Sweden. These data include digitized election rolls from the elections of 1994, 2010, and 2018. The pre-tsunami turnout measure comes from digitized electoral rolls collected and validated by researchers at Uppsala University^44^. They cover approximately 90% of the Swedish electorate in 1994. Which individuals are missing was therefore determined a decade before the tsunami and cannot depend on later exposure. Our restricted sample includes only individuals who appear in the 1994 file, so survivors and controls are subject to the same coverage, and the two groups have nearly identical 1994 turnout rates (Table 1). The data for the elections after the tsunami were digitized by the Swedish Election Authority and Statistics Sweden. As a matching strategy, we employ entropy balancing to ensure that the exposed and comparison groups are well balanced on relevant covariates.

Our dependent variable measures whether or not an individual voted in the studied elections. Our main explanatory variable is exposure to the tsunami, which is identified as being included in the tsunami registry. In all empirical models, we include control variables for income (log), education, sex, age, and age squared. These data were all based on Statistics Sweden’s registries. Sex is the legal sex recorded by Statistics Sweden, and the data contain no information on individuals’ gender.

To identify survivors in the bereaved group, we use the multigenerational register from Statistics Sweden. This enables us to link each individual to biological and adoptive parents, children, partners, siblings, and grandparents. Each relative was then cross-referenced with the official fatality records from the tsunami, enabling us to classify those whose relatives appeared in the records as bereaved. This linkage procedure identified 318 bereaved survivors: 179 lost one close relative, 94 lost two, 40 lost three, three lost four, and one survivor each lost five and six relatives. Sex was recorded as legal sex from Statistics Sweden’s population register, not self-reported. Sex was included only as a covariate in the entropy-balancing and regression models; we did not conduct a sex-disaggregated analysis, as sex differences are not the focus of our research question.

We analyze the register data with linear probability models, specifically least-squares regressions with a binary outcome (voted or not), weighted by the entropy-balancing weights. The coefficients of interest are differences in the probability of voting, in percentage points, between exposed and comparable unexposed individuals. Because the weights make exposure uncorrelated with the control variables, these coefficients do not depend on how the controls are specified. The errors of a linear probability model are heteroskedastic by construction, so instead of assuming equal variances we use heteroskedasticity-robust standard errors throughout. Inference relies on the approximately normal sampling distribution of the estimated coefficients when, as is the case here, samples are large. We therefore did not formally test normality. Logistic regression models give average marginal effects of similar size (Supplementary Table 7).

### Sampling strategies

No statistical method was used to predetermine sample size. The register data cover the entire Swedish population, and all individuals who met the eligibility criteria described below were included. As noted above, the linked registries let us observe individual-level turnout once before the tsunami and twice after it. Despite this advantage, selection into exposure could still bias our estimates if unobserved traits that predict trends in turnout also predict the likelihood of vacationing in the disaster-affected area. To address this concern and to balance the trade-offs between internal and external validity, we use two complementary sampling strategies. A challenge in our context is that the composition of the Swedish electorate changes substantially across decades due to, for example, the entry of new cohorts of voters and migration. Our sampling strategy is designed to minimize the risk that our results are driven by such compositional changes in the electorate.

First, our restricted eligibility strategy isolates individuals who were eligible to vote in 1994 and at a subsequent election. This approach constructs a stable sample that remains fixed over the entire period under study and thus avoids the problem of compositional changes that occur over time. Because all individuals in the cohort were already eligible voters in 1994, later differences in turnout can be more credibly interpreted as behavioral changes rather than demographic change within the electorate. Empirically, this strategy yields two distinct subsamples: a 1994–2010 eligibility subsample and a 1994–2018 eligibility subsample. Both subsamples thus maintain a stable baseline electorate.

Second, we implement an unrestricted eligibility strategy in which we construct separate electorate subsamples for each election year (1994, 2010, and 2018). Unlike the restricted cohort, this approach does not impose continuity in eligibility; instead, it reflects the natural evolution of the electorate. This is essential for the age-cohort analysis, which uses exposed (and controls) who were not yet eligible in 1994 but who constitute a substantial portion of the electorate in later years. It is also important for the analysis that uses bereavement as an indicator of the level of exposure (only 148 and 150 bereaved survivors can be identified in the restricted samples for 2010 and 2018, compared to 241 and 297 in the unrestricted samples). Income and education for individuals born 1986 or later are imputed from parental records, as these individuals were too young to have own pre-treatment socioeconomic data.

Taken together, the two sampling strategies form a layered empirical design. It is summarized in Table 4. As is clear, we use the restricted eligibility strategy, which enhances internal validity by minimizing cohort turnover and isolating behavioral continuity within a temporally stable population, in all but two analyses.

**Table 4:** Samples used in different models

	(1)	(2)
	Restricted eligibility samples	Unrestricted eligibility samples
Models presented in the article		
Effect of tsunami exposure on voter turnout, 2010 and 2018 (Table 2)	*✓*	
Effect of tsunami exposure and bereavement on voter turnout, 2010 and 2018 (Table 3)		*✓*
Effect of tsunami exposure on voter turnout by age at exposure, 2010 and 2018 (Fig. 2)		*✓*
Models presented in the Supplementary Information		
Effect of tsunami exposure on voter turnout, 2010 and 2018 (Logistic regression) (Supplementary Table 7)	*✓*	
Effect of tsunami exposure on voter turnout, 2010 and 2018 (Difference-in-difference) (Supplementary Table 9)	*✓*	
Effect of tsunami exposure on voter turnout by previous voting, 2010 and 2018 (Supplementary Fig. 1)	*✓*	
Effect of tsunami exposure on voter turnout by income, 2010 and 2018 (Supplementary Fig. 2)	*✓*	

Following Hainmueller^24^, the EB weights are combined with a weighted linear regression for the outcome. Because EB ensures that the treatment indicator is uncorrelated with the covariates used for balancing, including these covariates in the regression model can improve precision without changing the estimated treatment effect. For geographic adjustment, we balance on region (county). Balancing on smaller geographical units would risk unstable weights due to sparse overlap in exposure^24^.

We diagnose the performance of the entropy balancing procedure using standard weight and balance metrics^24^. The balancing constraints are exactly satisfied for all covariates and for region fixed effects, with weight concentration extremely low and no control units receiving disproportionate weight. Full diagnostics, including pre-/post-balance tables, weight distributions, Herfindahl indices, and effective sample sizes, are provided in Supplementary Tables 1–5 and Supplementary Notes 1, 2.

### Survey data

Using the registry data and matching variables from our main analysis, we conducted a survey of tsunami survivors and a matched control group. The survey was designed by the authors and administered by Statistics Sweden. The questionnaire and sampling strategy were developed in collaboration with Statistics Sweden, with careful consideration for the trauma experienced by those exposed. Participants were informed of the questionnaire’s subject matter prior to providing informed consent. Participants received no compensation.

The goal of the sampling strategy was to obtain two comparable groups, exposed and control, with respect to income, education, region of residence, sex, and age in 2003, prior to the tsunami. Data on these variables, including respondents’ sex and age, were taken from the same registers and follow the same definitions as in the register analyses. Table 5 presents descriptive statistics for the survey sample, including both the tsunami and control groups. Weighted and unweighted means are reported. As expected, individuals with higher levels of education and income were more likely to respond to the survey. However, as can be seen in Table 5, the weighted survey sample is considerably more similar to the tsunami and control populations that serve as the basis of the sampling frame.

**Table 5:** Summary statistics for the tsunami, control, and population samples

	Tsunami group		Control group		Population
	Weighted	Unweighted	Weighted	Unweighted	
Income (SEK)	278,762	301,530	259,591	280,346	203,088
Education	0.321	0.415	0.316	0.413	0.237
Age	40.27	42.42	39.95	42.13	48.71
Women	0.49	0.49	0.49	0.48	0.51

Data shows the weighted and unweighted mean values for all variables and is for the year prior to the tsunami (2003). Income and education data include those who were adults in 2003 and had an income or education to make comparisons with the main analytical sample easier (children under 18 are not included).

To preserve the integrity of the sampling process and minimize researcher influence, Statistics Sweden independently selected the final samples for both groups. The registry identified 16,030 tsunami survivors. To ensure eligibility, we excluded those who had died or emigrated since the event. We also excluded those who were very young when the tsunami occurred, specifically those born in 1995 or later. All respondents were therefore adults who were at least 28 years old at the time of the survey. This left 12,534 survivors eligible for matching. Each was paired with 10 randomly selected control individuals with identical sampling characteristics (exactly matched). Eleven survivors without complete matches were excluded, resulting in 12,523 survivors and 125,230 controls. From these, Statistics Sweden randomly selected 5000 individuals from each group. No statistical method was used to predetermine the survey sample size. We surveyed as many individuals as project resources allowed. We surveyed equal numbers of survivors and controls, which maximizes statistical power for a given total sample size.

The questionnaire covered attitudes about crisis preparedness, as well as more general questions about society, including the political attitudes and vote choices used in this paper. To the tsunami-exposed group, additional questions were posed focusing on their experiences during and after the disaster.

To classify individuals according to the severity of their exposure to the 2004 Indian Ocean tsunami, we constructed a measure of severe exposure grounded in established dimensions of disaster-related danger used in previous research. Earlier studies of Scandinavian tsunami survivors identify direct life threat, physical impact from the waves, serious injury, and loss or injury of close family members as important predictors of post-disaster psychological outcomes. We therefore define individuals as severely exposed if they experienced at least one of the following: (a) being caught, hit, or chased by the tsunami waves; (b) perceiving some or great danger of dying; (c) sustaining injuries requiring hospital admission; or (d) having a family member or close friend who was injured, caught by the waves, or killed in the disaster. Respondents who reported being entirely unaffected by the tsunami were excluded from the survey-based analyses to avoid diluting the contrast between the exposed and comparison groups. All exposure measures are described in Supplementary Note 3.

The survey was first distributed digitally on April 4, 2023, followed by an additional digital mailing and two postal follow-ups. The final reminder was sent on June 9, 2023. Response rates were 40.3% (2013 respondents) in the tsunami group and 38.9% (1943 respondents) in the control group, yielding an overall response rate of 39.6%. Our overall response rate (calculated following AAPOR RR2) is somewhat lower than Sweden’s largest and most well-resourced public opinion survey, the national SOM survey fielded annually by the SOM Institute at the University of Gothenburg, whose comparable rate was 47% in 2022^45^. The response rates in our two groups are nearly identical, which suggests that differential nonresponse between treated and control respondents is unlikely to drive our results. To reduce nonresponse bias, Statistics Sweden provided calibration weights that minimize the difference between the weighted respondents and the population in the same stratum (the tsunami population on the one hand, and the covariate-matched universe of controls on the other) with respect to relevant registry data; weighted and unweighted descriptive statistics are reported in Table 5, and descriptive statistics for the questions are presented in Supplementary Note 3.

We analyze the survey data with least-squares regressions of each outcome on indicators for exposure and severe exposure, weighted by these calibration weights and controlling for age, sex, education and income, with education and income measured in 2020. For the evaluation and trust items (0–4 scales), the coefficients are differences in mean ratings between exposed and unexposed respondents. For vote choice, they are differences in the probability of supporting each party, in percentage points. The exact matching of the two sampling frames, together with the calibration weights, makes the two groups similar on these characteristics. We include controls to adjust for the differences that remain. As for the register data, we use heteroskedasticity-robust standard errors and rely on the approximately normal sampling distribution of the estimated coefficients in large samples. Therefore, no formal tests of equal variance or normality are performed.

### Reporting summary

Further information on research design is available in the Nature Portfolio Reporting Summary linked to this article.

## Supplementary information

Supplementary Information

Reporting Summary

Transparent Peer Review File

## Source data

Source data

## Data Availability

The individual-level register data and survey microdata that support the findings of this study were supplied by Statistics Sweden under an agreement that prohibits public sharing, because they contain sensitive personal information on identifiable individuals (see Statistics Sweden’s terms of use for microdata access: https://www.scb.se/en/services/ordering-data-and-statistics/microdata/mona--statistics-swedens-platform-for-access-to-microdata/rules-and-regulations/terms-of-use/). The data are held on an encrypted remote-access server. Researchers may apply to Statistics Sweden for the same microdata (https://www.scb.se/en/services/ordering-data-and-statistics/microdata/); this requires prior approval from the Swedish Ethical Review Authority (Etikprövningsmyndigheten), and Statistics Sweden grants access for a specified research purpose and project period. At the time of writing, ethical review typically takes about 3 months and Statistics Sweden’s processing of a microdata order about 6 months, but processing times vary. Requests for guidance on replicating the data linkage can be directed to the corresponding author. The data underlying Fig. 1 are provided in Supplementary Table 14. The SOM survey data used in Supplementary Table 13 (National SOM Survey Cumulative Dataset 1986–2022, version 13) are publicly available from the SOM Institute, University of Gothenburg, via the Swedish National Data Service (https://snd.se).  Source data are provided with this paper.

## Code availability

The Stata do-files, log files and R scripts that reproduce all analyses, and that document all variables used, are available at Harvard Dataverse, https://doi.org/10.7910/DVN/0AF9U0.
