# Supplementary material for: Long-term electoral participation among Swedish survivors of the 2004 Indian Ocean tsunami
Source: Nat Commun. 2026 Oct 22;17:11038. doi: 10.1038/s41467-026-78593-8 (PMC13691066; doi:10.1038/s41467-026-78593-8)
Supplement: Supplementary file 1 [file 41467_2026_78593_MOESM1_ESM.pdf]

Supplementary Information "Long-term electoral participation among Swedish survivors of the 2004 Indian Ocean tsunami"

Contents

Supplementary Tables 1
Supplementary Figures 15
Supplementary Note 1: Entropy balancing weight diagnostics for the restricted sample 17
Supplementary Note 2: Entropy balancing weight diagnostics for the unrestricted sample 19
Supplementary Note 3: Survey Analyses 22

Supplementary Tables

Table 1: Covariate and Regional Balance Before and After Entropy Balancing: Restricted Sample, 1994-2010

	Before EB			After EB		
Covariate / Region	Treated	Control	Diff	Treated	Control	Diff
Sex (female)	0.461	0.512	-0.051	0.461	0.461	0.000
Income (log)	7.728	7.497	0.231	7.728	7.728	0.000
Education	0.363	0.249	0.114	0.363	0.363	0.000
Age (years)	51.860	58.074	-6.214	51.860	51.870	-0.010
Age^2	2804.656	3597.671	-793.015	2804.656	2805.966	-1.311
Turnout 1994 (%)	89.223	89.164	0.059	89.223	89.225	-0.002
Region (entropy-balanced but not shown in Table 1)						
Stockholm (1)	0.333	0.197	0.136	0.333	0.333	0.000
Uppsala (3)	0.041	0.036	0.004	0.041	0.041	0.000
Södermanland (4)	0.022	0.028	-0.006	0.022	0.022	0.000
Östergötland (5)	0.035	0.052	-0.017	0.035	0.035	0.000
Jönköping (6)	0.031	0.040	-0.009	0.031	0.031	0.000
Kronoberg (7)	0.010	0.019	-0.008	0.010	0.010	0.000
Kalmar (8)	0.016	0.029	-0.012	0.016	0.016	0.000
Gotland (9)	0.009	0.008	0.001	0.009	0.009	0.000
Blekinge (10)	0.007	0.016	-0.009	0.007	0.007	0.000
Skåne (12)	0.089	0.133	-0.044	0.089	0.089	0.000
Halland (13)	0.037	0.027	0.010	0.037	0.037	0.000
Västra Götaland (14)	0.210	0.162	0.048	0.210	0.210	0.000
Värmland (17)	0.015	0.031	-0.016	0.015	0.015	0.000
Örebro (18)	0.021	0.028	-0.007	0.021	0.021	0.000
Västmanland (19)	0.018	0.030	-0.012	0.018	0.018	0.000
Dalarna (20)	0.020	0.031	-0.012	0.020	0.020	0.000
Gävleborg (21)	0.020	0.028	-0.008	0.020	0.020	0.000
Västernorrland (22)	0.015	0.028	-0.013	0.015	0.015	0.000
Jämtland (23)	0.008	0.015	-0.007	0.008	0.008	0.000
Västerbotten (24)	0.020	0.031	-0.010	0.020	0.020	0.000
Norrbotten (25)	0.021	0.030	-0.009	0.021	0.021	0.000

Notes. The table reports covariate and regional means for tsunami-exposed individuals (Treated) and unexposed controls (Control) before and after entropy balancing (EB), for the restricted sample used in the 1994-2010 analysis (Table 1, Panel A). The sample is restricted to individuals who appear in the 1994 turnout file and were thus eligible to vote before the disaster. Before EB, columns show unweighted means; notable imbalances exist on income, education, and age. After entropy balancing, the control group is reweighted so that the means of all pre-treatment covariates and regional indicators match those of the treated group exactly. Turnout is rescaled to percentage points (0–100). All covariates are measured in 2003, prior to the tsunami. Region is the county (SCB Länskod) of residence in 2003; codes follow the SCB standard and are not consecutive because some historical counties have since merged into others.

Table 2: Covariate and Regional Balance Before and After Entropy Balancing: Restricted Sample, 1994-2018

	Before EB			After EB		
Covariate / Region	Treated	Control	Diff	Treated	Control	Diff
Sex (female)	0.461	0.511	-0.049	0.461	0.461	0.000
Income (log)	7.734	7.547	0.187	7.734	7.734	0.000
Education	0.368	0.266	0.102	0.368	0.368	0.000
Age (years)	59.341	63.037	-3.696	59.341	59.342	-0.001
Age^2	3628.173	4144.461	-516.288	3628.173	3628.245	-0.072
Turnout 1994 (%)	89.236	89.064	0.173	89.236	89.236	0.000
Region (entropy-balanced but not shown in Table 1)						
Stockholm (1)	0.344	0.206	0.138	0.344	0.344	0.000
Uppsala (3)	0.040	0.036	0.003	0.040	0.040	0.000
Södermanland (4)	0.021	0.027	-0.006	0.021	0.021	0.000
Östergötland (5)	0.034	0.051	-0.017	0.034	0.034	0.000
Jönköping (6)	0.030	0.039	-0.009	0.030	0.030	0.000
Kronoberg (7)	0.010	0.018	-0.008	0.010	0.010	0.000
Kalmar (8)	0.016	0.027	-0.012	0.016	0.016	0.000
Gotland (9)	0.008	0.007	0.000	0.008	0.008	0.000
Blekinge (10)	0.007	0.016	-0.009	0.007	0.007	0.000
Skåne (12)	0.087	0.130	-0.043	0.087	0.087	0.000
Halland (13)	0.036	0.027	0.009	0.036	0.036	0.000
Västra Götaland (14)	0.211	0.165	0.046	0.211	0.211	0.000
Värmland (17)	0.014	0.030	-0.016	0.014	0.014	0.000
Örebro (18)	0.020	0.027	-0.007	0.020	0.020	0.000
Västmanland (19)	0.017	0.029	-0.012	0.017	0.017	0.000
Dalarna (20)	0.019	0.030	-0.011	0.019	0.019	0.000
Gävleborg (21)	0.023	0.033	-0.010	0.023	0.023	0.000
Västernorrland (22)	0.014	0.027	-0.013	0.014	0.014	0.000
Jämtland (23)	0.008	0.015	-0.007	0.008	0.008	0.000
Västerbotten (24)	0.020	0.030	-0.010	0.020	0.020	0.000
Norrbotten (25)	0.021	0.029	-0.008	0.021	0.021	0.000

Notes. The table reports covariate and regional means for tsunami-exposed individuals (Treated) and unexposed controls (Control) before and after entropy balancing (EB), for the restricted sample used in the 1994-2018 analysis (Table 1, Panel B). The sample is restricted to individuals who appear in the 1994 turnout file and were thus eligible to vote before the disaster. Before EB, columns show unweighted means; notable imbalances exist on income, education, and age. After entropy balancing, the control group is reweighted so that the means of all pre-treatment covariates and regional indicators match those of the treated group exactly. Turnout is rescaled to percentage points (0–100). All covariates are measured in 2003, prior to the tsunami. Region is the county (SCB Länskod) of residence in 2003; codes follow the SCB standard and are not consecutive because some historical counties have since merged into others.

Table 3: Covariate and Regional Balance Before and After Entropy Balancing: Unrestricted Sample, 1994 Election (Pre-Treatment Placebo)

		Before EB			After EB	
Covariate / Region	Treated	Control	Diff	Treated	Control	Diff
Sex (female)	0.459	0.513	-0.054	0.459	0.459	0.000
Income (log)	7.728	7.460	0.268	7.728	7.728	0.000
Education	0.363	0.239	0.124	0.363	0.363	0.000
Age (years)	45.082	53.629	-8.547	45.082	45.083	-0.001
Age^2	2151.149	3152.488	-1001.339	2151.149	2151.295	-0.146
Region (entropy-balanced, not shown in Table 3)						
Stockholm (1)	0.341	0.200	0.141	0.341	0.341	0.000
Uppsala (3)	0.040	0.035	0.005	0.040	0.040	0.000
Södermanland (4)	0.021	0.028	-0.006	0.021	0.021	0.000
Östergötland (5)	0.033	0.051	-0.018	0.033	0.033	0.000
Jönköping (6)	0.030	0.039	-0.009	0.030	0.030	0.000
Kronoberg (7)	0.010	0.018	-0.008	0.010	0.010	0.000
Kalmar (8)	0.016	0.028	-0.013	0.016	0.016	0.000
Gotland (9)	0.008	0.007	0.001	0.008	0.008	0.000
Blekinge (10)	0.007	0.016	-0.009	0.007	0.007	0.000
Skåne (12)	0.087	0.131	-0.045	0.087	0.087	0.000
Halland (13)	0.036	0.026	0.010	0.036	0.036	0.000
Västra Götaland (14)	0.212	0.165	0.046	0.212	0.212	0.000
Värmland (17)	0.014	0.031	-0.016	0.014	0.014	0.000
Örebro (18)	0.021	0.028	-0.007	0.021	0.021	0.000
Västmanland (19)	0.017	0.029	-0.012	0.017	0.017	0.000
Dalarna (20)	0.020	0.031	-0.011	0.020	0.020	0.000
Gävleborg (21)	0.023	0.034	-0.011	0.023	0.023	0.000
Västernorrland (22)	0.015	0.028	-0.013	0.015	0.015	0.000
Jämtland (23)	0.008	0.015	-0.007	0.008	0.008	0.000
Västerbotten (24)	0.020	0.030	-0.010	0.020	0.020	0.000
Norrbotten (25)	0.020	0.029	-0.009	0.020	0.020	0.000

Notes. Treated N = 8,065; Control N = 5,003,473. The table reports covariate and regional means for tsunami-exposed individuals (Treated) and unexposed controls (Control) before and after entropy balancing (EB), for the unrestricted sample, 1994 Election (Pre-Treatment Placebo).This is a pre-treatment placebo check: 1994 precedes the 2004 tsunami, so entropy balancing here tests whether tsunami-exposed and unexposed individuals already differed on these covariates before the disaster. The estimated difference in 1994 turnout between exposed and unexposed individuals is small and not statistically significant (beta = 0.005, $t(5{,}011{,}511) = 1.46$, $p = 0.144$, 95% CI = $[-0.002, 0.012]$).

Table 4: Covariate and Regional Balance Before and After Entropy Balancing: Unrestricted Sample, 2010

	Before EB			After EB		
Covariate / Region	Treated	Control	Diff	Treated	Control	Diff
Sex (female)	0.484	0.507	-0.023	0.484	0.484	0.000
Income (log)	7.580	7.370	0.210	7.580	7.580	0.000
Education	0.361	0.254	0.107	0.361	0.361	0.000
Age (years)	42.950	49.803	-6.852	42.950	42.962	-0.012
Age^2	2085.548	2845.026	-759.477	2085.548	2086.916	-1.368
Region (entropy-balanced, not shown in Table 3)						
Stockholm (1)	0.319	0.195	0.124	0.319	0.319	0.000
Uppsala (3)	0.042	0.035	0.007	0.042	0.042	0.000
Södermanland (4)	0.023	0.030	-0.007	0.023	0.023	0.000
Östergötland (5)	0.036	0.049	-0.013	0.036	0.036	0.000
Jönköping (6)	0.028	0.038	-0.010	0.028	0.028	0.000
Kronoberg (7)	0.011	0.021	-0.010	0.011	0.011	0.000
Kalmar (8)	0.016	0.028	-0.012	0.016	0.016	0.000
Gotland (9)	0.008	0.007	0.001	0.008	0.008	0.000
Blekinge (10)	0.007	0.018	-0.011	0.007	0.007	0.000
Skåne (12)	0.088	0.131	-0.042	0.088	0.088	0.000
Halland (13)	0.047	0.033	0.014	0.047	0.047	0.000
Västra Götaland (14)	0.215	0.164	0.052	0.215	0.215	0.000
Värmland (17)	0.016	0.031	-0.015	0.016	0.016	0.000
Örebro (18)	0.024	0.032	-0.008	0.024	0.024	0.000
Västmanland (19)	0.019	0.030	-0.011	0.019	0.019	0.000
Dalarna (20)	0.021	0.032	-0.011	0.021	0.021	0.000
Gävleborg (21)	0.019	0.025	-0.007	0.019	0.019	0.000
Västernorrland (22)	0.015	0.029	-0.014	0.015	0.015	0.000
Jämtland (23)	0.008	0.015	-0.007	0.008	0.008	0.000
Västerbotten (24)	0.021	0.030	-0.009	0.021	0.021	0.000
Norrbotten (25)	0.018	0.029	-0.011	0.018	0.018	0.000

Notes. Treated N = 12,889; Control N = 6,689,047. The table reports covariate and regional means for tsunami-exposed individuals (Treated) and unexposed controls (Control) before and after entropy balancing (EB), for the unrestricted sample, 2010. The unrestricted sample includes all eligible voters in 2010, including those who were not yet of voting age in 1994. Income and education for individuals born after 1986 are imputed from parental records.

Table 5: Covariate and Regional Balance Before and After Entropy Balancing: Unrestricted Sample, 2018

	Before EB			After EB		
Covariate / Region	Treated	Control	Diff	Treated	Control	Diff
Sex (female)	0.489	0.505	-0.016	0.489	0.489	0.000
Income (log)	7.615	7.418	0.197	7.615	7.615	0.000
Education	0.383	0.282	0.101	0.383	0.383	0.000
Age (years)	47.691	51.250	-3.559	47.691	47.694	-0.003
Age^2	2551.279	2996.266	-444.987	2551.279	2551.630	-0.351
Region (entropy-balanced, not shown in Table 3)						
Stockholm (1)	0.334	0.206	0.128	0.334	0.334	0.000
Uppsala (3)	0.041	0.035	0.006	0.041	0.041	0.000
Södermanland (4)	0.021	0.029	-0.008	0.021	0.021	0.000
Östergötland (5)	0.034	0.047	-0.013	0.034	0.034	0.000
Jönköping (6)	0.026	0.037	-0.011	0.026	0.026	0.000
Kronoberg (7)	0.011	0.020	-0.009	0.011	0.011	0.000
Kalmar (8)	0.015	0.026	-0.011	0.015	0.015	0.000
Gotland (9)	0.008	0.007	0.001	0.008	0.008	0.000
Blekinge (10)	0.006	0.017	-0.011	0.006	0.006	0.000
Skåne (12)	0.086	0.128	-0.042	0.086	0.086	0.000
Halland (13)	0.045	0.032	0.013	0.045	0.045	0.000
Västra Götaland (14)	0.218	0.169	0.049	0.218	0.218	0.000
Värmland (17)	0.015	0.030	-0.015	0.015	0.015	0.000
Örebro (18)	0.022	0.030	-0.008	0.022	0.022	0.000
Västmanland (19)	0.017	0.029	-0.012	0.017	0.017	0.000
Dalarna (20)	0.020	0.031	-0.010	0.020	0.020	0.000
Gävleborg (21)	0.022	0.031	-0.009	0.022	0.022	0.000
Västernorrland (22)	0.013	0.027	-0.014	0.013	0.013	0.000
Jämtland (23)	0.008	0.014	-0.006	0.008	0.008	0.000
Västerbotten (24)	0.019	0.029	-0.010	0.019	0.019	0.000
Norrbotten (25)	0.018	0.028	-0.010	0.018	0.018	0.000

Notes. Treated N = 14,668; Control N = 7,024,100. The table reports covariate and regional means for tsunami-exposed individuals (Treated) and unexposed controls (Control) before and after entropy balancing (EB), for the unrestricted sample, 2018. The unrestricted sample includes all eligible voters in 2018, including those who were not yet of voting age in 1994. Income and education for individuals born prior to 1986 are imputed from parental records.

Table 6: Full Coefficient Estimates for the Main Model of Tsunami Exposure and Voter Turnout

	2010 election				2018 election			
Variable	Estimate	95% CI	t	p	Estimate	95% CI	t	p
Tsunami	1.444	0.876 to 2.011	4.98	<0.001	1.442	0.924 to 1.961	5.45	<0.001
Women	2.331	1.748 to 2.915	7.83	<0.001	1.152	0.611 to 1.692	4.18	<0.001
Income (SEK)	2.149	1.692 to 2.606	9.22	<0.001	1.646	1.245 to 2.048	8.03	<0.001
Education	3.847	3.323 to 4.370	14.40	<0.001	3.258	2.775 to 3.741	13.22	<0.001
Age	0.211	-0.041 to 0.464	1.64	0.101	1.041	0.672 to 1.411	5.52	<0.001
Age squared	-0.002	-0.005 to 0.000	-1.84	0.066	-0.010	-0.013 to -0.007	-6.38	<0.001
Voted 1994	27.542	25.923 to 29.162	33.33	<0.001	20.196	18.746 to 21.645	27.30	<0.001
Constant	42.677	35.541 to 49.813	11.72	<0.001	34.628	23.763 to 45.494	6.25	<0.001
Region fixed effects	Yes				Yes			
N, total	4,374,679				3,880,158			
N, exposed	7,674				7,646			
N, unexposed	4,367,005				3,872,512			
Adjusted R^2	0.122				0.088			

Notes. Full coefficient estimates for the covariate-adjusted, entropy-balancing-weighted linear regression of voter turnout on tsunami exposure reported in Table 2 (restricted sample). Linear probability models estimated by weighted least squares with entropy-balancing weights and heteroskedasticity-robust standard errors. Estimates are in percentage points (0–100) except for the constant. CI, confidence interval. t statistics and p-values are from two-sided t-tests of the null hypothesis that the estimate equals zero; no adjustment for multiple comparisons was made. Region fixed effects are included in both models (2010: 20 region dummies; 2018: 20 region dummies, one region omitted as reference in each) but are not reported individually. Sample: restricted, that is, individuals eligible to vote in both 1994 and the election shown (see Methods). Source data are provided as a Source Data file.

Table 7: Full Coefficient Estimates for the Main Model of Tsunami Exposure and Voter Turnout (Logistic Regression)

	2010 election				2018 election			
Variable	OR	95% CI	z	p	OR	95% CI	z	p
Tsunami	1.268	1.159 to 1.387	5.18	<0.001	1.312	1.188 to 1.448	5.38	<0.001
Women	1.347	1.238 to 1.466	6.91	<0.001	1.189	1.085 to 1.303	3.71	<0.001
Income (SEK)	1.213	1.174 to 1.253	11.60	<0.001	1.194	1.154 to 1.236	10.12	<0.001
Education	2.171	1.952 to 2.414	14.32	<0.001	2.144	1.908 to 2.409	12.82	<0.001
Age	1.049	1.015 to 1.084	2.88	0.004	1.137	1.089 to 1.187	5.88	<0.001
Age squared	1.000	0.999 to 1.000	-3.08	0.002	0.999	0.998 to 0.999	-7.30	<0.001
Voted 1994	7.838	7.148 to 8.594	43.80	<0.001	6.805	6.144 to 7.537	36.79	<0.001
Constant	0.108	0.044 to 0.265	-4.87	<0.001	0.027	0.007 to 0.102	-5.34	<0.001
Region fixed effects	Yes				Yes			
N, total	4,374,679				3,880,158			
N, exposed	7,674				7,646			
N, unexposed	4,367,005				3,872,512			
Pseudo R^2 (McFadden)	0.150				0.132			

Notes. Full coefficient estimates for the covariate-adjusted, entropy-balancing-weighted logistic regression of voter turnout on tsunami exposure (logit link, weighted maximum likelihood with entropy-balancing weights and heteroskedasticity-robust standard errors). Estimates are odds ratios (exponentiated coefficients); an odds ratio above 1 indicates higher odds of turnout. CI, confidence interval (exponentiated). z statistics and p-values are from two-sided Wald tests of the null hypothesis that the underlying log-odds coefficient equals zero; no adjustment for multiple comparisons was made. Pseudo R^2 is McFadden's pseudo R-squared. Average marginal effects of tsunami exposure from these models, in percentage points: 1.617 (95% CI 1.053 to 2.182; $z = 5.61$, $p < 0.001$) in 2010 and 1.561 (95% CI 1.047 to 2.075; $z = 5.96$, $p < 0.001$) in 2018. Region fixed effects are included in both models (2010: 20 region dummies; 2018: 20 region dummies, one region omitted as reference in each) but are not reported individually. Sample: restricted, that is, individuals eligible to vote in both 1994 and the election shown (see Methods). Source data are provided as a Source Data file.

Table 8: Composition of Bereaved Survivors by Relationship to Deceased

	N	Share (%)
Parent	132	41.5
Child	88	27.7
Sibling	80	25.2
Partner	75	23.6
Grandparent	37	11.6
Total bereaved survivors	318	

Notes. The table reports the relationship to the deceased. Categories are not mutually exclusive; some survivors lost relatives in more than one category and more than one relative in the same category (only counted once in the table). Relationship categories include both biological and adoptive relatives. See Methods for details on the linkage procedure.

Table 9: Difference-in-Differences Estimates of Voting Behavior (Restricted Samples)

	(1) 1994–2010				(2) 1994–2018			
Variable	Estimate	95% CI	t	p	Estimate	95% CI	t	p
Tsunami × Post (Tsunami × After 2004)	1.444	0.844 to 2.045	4.71	<0.001	1.443	0.906 to 1.980	5.26	<0.001
Election year: 2010	89.806	89.775 to 89.837	5640.90	<0.001	–	–	–	–
Election year: 2018	–	–	–	–	91.531	91.503 to 91.560	6260.47	<0.001
Constant	0.892	0.742 to 1.042	11.65	<0.001	0.892	0.758 to 1.027	13.03	<0.001
Year FE		Yes				Yes		
Individual FE		Yes				Yes		
EB-weights		Yes				Yes		
N		8,749,358				7,760,316		
Adjusted R^2		0.913				0.931		

Notes. Two-way fixed-effects difference-in-differences estimates of voting behavior using election data from 1994 and 2010 (Model 1), and 1994 and 2018 (Model 2). Individual and election-year fixed effects; entropy-balancing probability weights; standard errors clustered by individual. CI, confidence interval. t statistics and p-values are from two-sided t-tests of the null hypothesis that the estimate equals zero; no adjustment for multiple comparisons was made. Sample: restricted, that is, individuals eligible to vote in both 1994 and the election shown (see Methods).

Table 10: Tsunami × Previous Voting (Entropy-Balanced OLS with Robust Standard Errors)

	(1) 2010				(2) 2018			
Variable	Estimate	95% CI	t	p	Estimate	95% CI	t	p
Tsunami exposure	5.041	1.874 to 8.207	3.12	0.002	6.680	3.858 to 9.502	4.64	$<$0.001
Voted in 1994	29.547	29.284 to 29.809	220.68	$<$0.001	23.116	22.875 to 23.357	188.18	$<$0.001
Tsunami × Voted in 1994	-4.032	-7.242 to -0.821	-2.46	0.014	-5.869	-8.732 to -3.006	-4.02	$<$0.001
Income (log)	2.157	1.700 to 2.613	9.26	$<$0.001	1.654	1.254 to 2.053	8.11	$<$0.001
Education	3.858	3.335 to 4.381	14.47	$<$0.001	3.276	2.793 to 3.758	13.31	$<$0.001
Age	0.217	-0.036 to 0.470	1.68	0.093	1.053	0.684 to 1.423	5.59	$<$0.001
Age^2	-0.002	-0.005 to 0.000103	-1.87	0.061	-0.010	-0.013 to -0.007	-6.43	$<$0.001
Sex (female)	2.338	1.755 to 2.921	7.86	$<$0.001	1.160	0.620 to 1.700	4.21	$<$0.001
Intercept	40.653	33.619 to 47.688	11.33	$<$0.001	31.556	20.698 to 42.415	5.70	$<$0.001
Region FE	Yes				Yes			
EB-weights	Yes				Yes			
N	4,374,679				3,880,158			
Adjusted R^2	0.122				0.089			

Notes. Entropy-balanced linear probability models of voter turnout (percentage points, 0–100), with tsunami exposure interacted with previous voting behavior (voted in 1994). CI, confidence interval. t statistics and p-values are from two-sided t-tests of the null hypothesis that the estimate equals zero; no adjustment for multiple comparisons was made. Region fixed effects are included but not reported individually. Sample: restricted (see Methods).

Table 11: Tsunami × Income (Entropy-Balanced OLS with Robust Standard Errors)

	(1) 2010				(2) 2018			
Variable	Estimate	95% CI	t	p	Estimate	95% CI	t	p
Tsunami exposure	21.500	16.477 to 26.522	8.39	<0.001	18.022	13.562 to 22.483	7.92	<0.001
Income (log)	3.938	3.816 to 4.060	63.03	<0.001	3.114	3.004 to 3.224	55.41	<0.001
Tsunami × Income (log)	-2.595	-3.224 to -1.966	-8.08	<0.001	-2.144	-2.700 to -1.587	-7.55	<0.001
Sex (female)	2.515	1.939 to 3.091	8.56	<0.001	1.300	0.766 to 1.835	4.77	<0.001
Education	3.700	3.181 to 4.218	13.98	<0.001	3.138	2.658 to 3.618	12.81	<0.001
Age	0.187	-0.066 to 0.439	1.45	0.148	1.025	0.655 to 1.395	5.42	<0.001
Age^2	-0.002	-0.004 to 0.000386	-1.63	0.102	-0.010	-0.013 to -0.007	-6.27	<0.001
Voted in 1994	27.421	25.799 to 29.043	33.14	<0.001	20.098	18.647 to 21.550	27.13	<0.001
Intercept	29.492	23.116 to 35.869	9.06	<0.001	23.764	13.301 to 34.226	4.45	<0.001
Region FE	Yes				Yes			
EB-weights	Yes				Yes			
N	4,374,679				3,880,158			
Adjusted R^2	0.124				0.089			

Notes. Entropy-balanced linear probability models of voter turnout (percentage points, 0–100), with tsunami exposure interacted with log income. CI, confidence interval. t statistics and p-values are from two-sided t-tests of the null hypothesis that the estimate equals zero; no adjustment for multiple comparisons was made. Region fixed effects are included but not reported individually. Sample: restricted (see Methods).

Table 12: Full Entropy-Balanced Interaction Model of Tsunami Exposure, Age Group, and Voter Turnout (Unrestricted Sample)

	Estimate	95% CI	t	p
**2010 election**				
Tsunami exposure	3.256	1.336 to 5.177	3.32	<0.001
Age groups (<15 as ref. group)				
16-25	2.879	2.552 to 3.205	17.28	<0.001
26-50	6.979	6.379 to 7.580	22.79	<0.001
>50	7.662	6.147 to 9.177	9.91	<0.001
Tsunami × age groups (<15 as ref. group)				
Tsunami × 16-25	1.228	-1.114 to 3.571	1.03	0.304
Tsunami × 26-50	-1.594	-3.640 to 0.452	-1.53	0.127
Tsunami × >50	-0.534	-2.680 to 1.612	-0.49	0.626
Sex (female)	2.546	2.017 to 3.075	9.44	<0.001
Income (log)	3.090	2.704 to 3.475	15.71	<0.001
Education	5.552	5.037 to 6.066	21.16	<0.001
Age^2	0.000292	-0.0000871 to 0.001	1.51	0.131
Intercept	54.852	51.761 to 57.944	34.78	<0.001
Region FE		Yes		
EB-weights		Yes		
N, total		6701936		
Adjusted R^2		0.032		
**2018 election**				
Tsunami exposure	1.746	0.641 to 2.852	3.10	0.002
Age groups (<15 as ref. group)				
16-25	4.876	4.544 to 5.209	28.72	<0.001
26-50	7.504	6.714 to 8.294	18.62	<0.001
>50	9.330	7.562 to 11.098	10.34	<0.001
Tsunami × age groups (<15 as ref. group)				
Tsunami × 16-25	1.854	0.378 to 3.329	2.46	0.014
Tsunami × 26-50	0.130	-1.123 to 1.384	0.20	0.839
Tsunami × >50	0.773	-0.732 to 2.278	1.01	0.314
Sex (female)	2.478	2.044 to 2.912	11.19	<0.001
Income (log)	2.620	2.285 to 2.954	15.35	<0.001
Education	3.697	3.268 to 4.127	16.86	<0.001
Age^2	-0.002	-0.002 to -0.001	-7.89	<0.001
Intercept	65.261	62.618 to 67.905	48.38	<0.001
Region FE		Yes		
EB-weights		Yes		
N, total		7038768		
Adjusted R^2		0.022		

Notes. Full entropy-balanced linear probability model of voter turnout, with tsunami exposure interacted with age group at the time of the tsunami (reference: 15 or under). "Tsunami exposure" is the tsunami effect for the reference age group (15 or under); the "Age groups" rows are the age-group main effects; the "Tsunami × age groups" rows are the interaction terms. The age-group-specific tsunami effects plotted in Figure 2 (and reported exactly in the accompanying Source Data file) are the sum of "Tsunami exposure" and the relevant interaction term (e.g., the effect for ages 16–25 is "Tsunami exposure" plus "Tsunami × 16-25"). CI, confidence interval. t statistics and p-values are from two-sided t-tests of the null hypothesis that the estimate equals zero; no adjustment for multiple comparisons was made. Region fixed effects are included but not reported individually. Sample: unrestricted (see Methods).

Table 13: Traveled Abroad and Voting in National Elections

	(1) No FE				(2) With FE			
Variable	Estimate	95% CI	t	p	Estimate	95% CI	t	p
Traveled Abroad (last 12 mo.)	0.580	-0.694 to 1.853	0.89	0.372	0.621	-0.685 to 1.927	0.93	0.351
Household Income (quintile)	1.718	1.185 to 2.251	6.32	$<$0.001	1.716	1.182 to 2.250	6.30	$<$0.001
Education	3.044	1.789 to 4.299	4.75	$<$0.001	3.186	1.896 to 4.475	4.84	$<$0.001
Sex (female = 1)	-2.749	-3.937 to -1.561	-4.54	$<$0.001	-2.721	-3.911 to -1.532	-4.49	$<$0.001
Age	0.542	0.287 to 0.797	4.16	$<$0.001	0.555	0.301 to 0.809	4.29	$<$0.001
Age^2	-0.003	-0.006 to -0.001	-2.68	0.007	-0.004	-0.006 to -0.001	-2.79	0.005
Intercept	67.942	61.655 to 74.228	21.19	$<$0.001	69.378	62.950 to 75.806	21.16	$<$0.001
Year FE	No				Yes			
Region FE	No				Yes			
N	6928				6928			
Adjusted R^2	0.034				0.036			

Notes. Linear probability models of self-reported turnout on having traveled abroad in the last 12 months. Elections included: 1994, 1998, 2006, 2010 (other years lack travel data). The dependent variable is rescaled to percentage points (0–100) for ease of interpretation. Model (1) includes no fixed effects; Model (2) adds region and election-year fixed effects. 95% confidence intervals (in brackets) and exact p-values are from two-sided t-tests of the null hypothesis that the estimate equals zero, based on heteroskedasticity-robust standard errors; no adjustment for multiple comparisons was made. Source: University of Gothenburg, SOM Institute (2024), National SOM Survey Cumulative Dataset 1986–2022 (Version 13) [1].

Table 14: Number of Casualties per 10 Million Inhabitants in the 2004 Boxing Day Tsunami per Country

Country	Deaths	2004 Population	Deaths per 10 Million	Source
Austria	86	8,171,966	105.24	[2]
Belgium	11	10,421,137	10.56	[3]
Croatia	1	4,304,600	2.32	[4]
Czechia	8	10,197,101	7.85	[5]
Denmark	46	5,404,523	85.11	[6]
Estonia	3	1,362,550	22.02	[7]
Finland	179	5,228,172	342.38	[8]
France	95	62,716,306	15.15	[9]
Germany	539	82,516,260	65.32	[10]
Ireland	3	4,070,262	7.37	[11]
Italy	54	57,685,327	9.36	[12]
Luxembourg	2	458,095	43.66	[13]
Malta	1	401,268	24.92	[14]
Netherlands	36	16,281,779	22.11	[15]
Norway	84	4,591,910	182.93	[6]
Poland	1	38,182,222	0.26	[16]
Portugal	5	10,483,861	4.77	[17]
Russia	10	144,067,316	0.69	[18]
Spain	2	42,921,895	0.47	[19]
Sweden	543	8,993,531	603.77	[6]
Switzerland	113	7,389,625	152.92	[20]
Turkey	1	67,785,075	0.15	[21]
United Kingdom	151	59,987,905	25.17	[22]

Notes: Death tolls are drawn primarily from official government sources (e.g., ministries of foreign affairs, national investigative reports) or peer-reviewed publications; where no such source was available, secondary sources (news media) were used instead (see Supplementary References for sources). Population data are 2004 total population figures from World Bank Data – Population, total [23].

Supplementary Figures

Figure 1: Effect of Tsunami Exposure on Voter Turnout by Previous Voting, 2010 and 2018

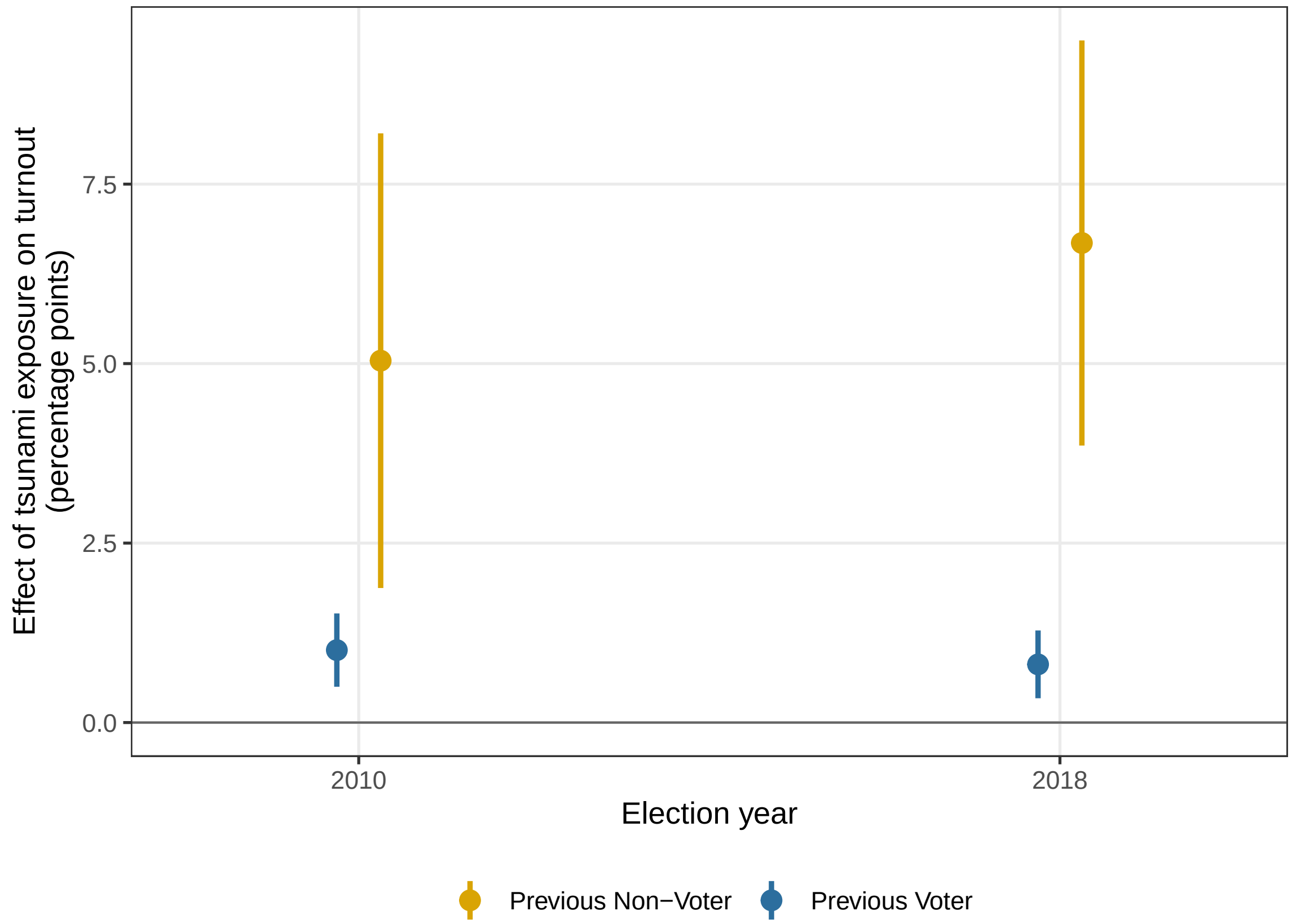

Notes: Points represent the average marginal effect of tsunami exposure on voter turnout (percentage points), estimated separately for previous voters and previous non-voters (voted in 1994 or not), from an entropy-balanced linear probability model including region fixed effects and covariates (sex, log income, education, age^2). Error bars represent 95% confidence intervals; robust standard errors. Sample: restricted (see Methods); n = 4,374,679 (2010) and n = 3,880,158 (2018). P values are two-sided, not adjusted for multiple comparisons, and reported exactly in Supplementary Table 10, which also reports the full underlying model.

Figure 2: Effect of Tsunami Exposure on Voter Turnout by Income, 2010 and 2018

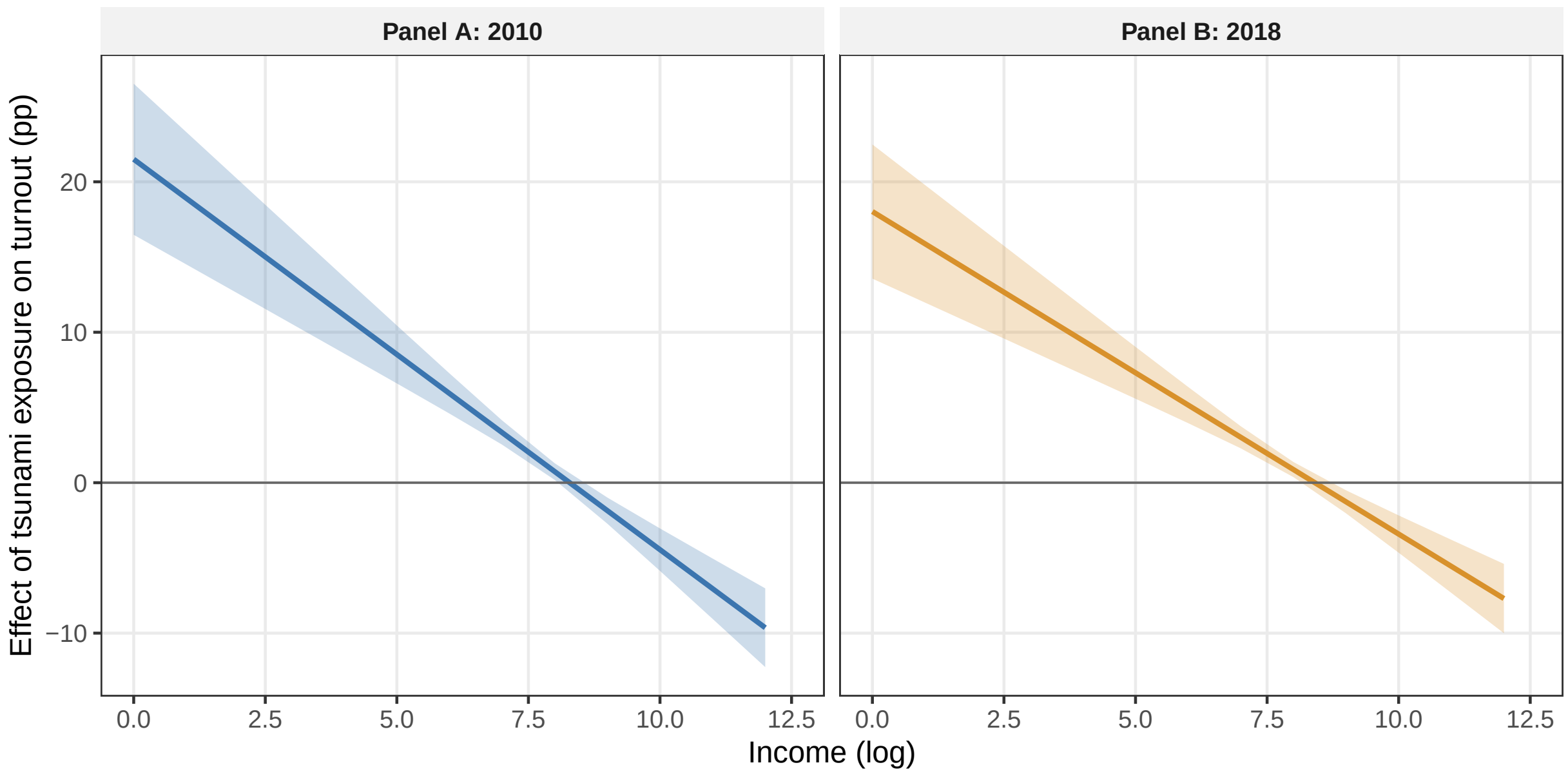

Notes: Lines represent the predicted marginal effect of tsunami exposure on voter turnout (percentage points) across the range of log income, from an entropy-balanced linear probability model including region fixed effects and covariates (sex, education, age^2). Shaded bands represent 95% confidence intervals; robust standard errors. Income is logged to limit the influence of outliers. Sample: restricted (see Methods); n = 4,374,679 (2010, Panel A) and n = 3,880,158 (2018, Panel B). P values are two-sided, not adjusted for multiple comparisons, and reported exactly in Supplementary Table 11, which also reports the full underlying model.

Supplementary Note 1: Entropy balancing weight diagnostics for restricted sample

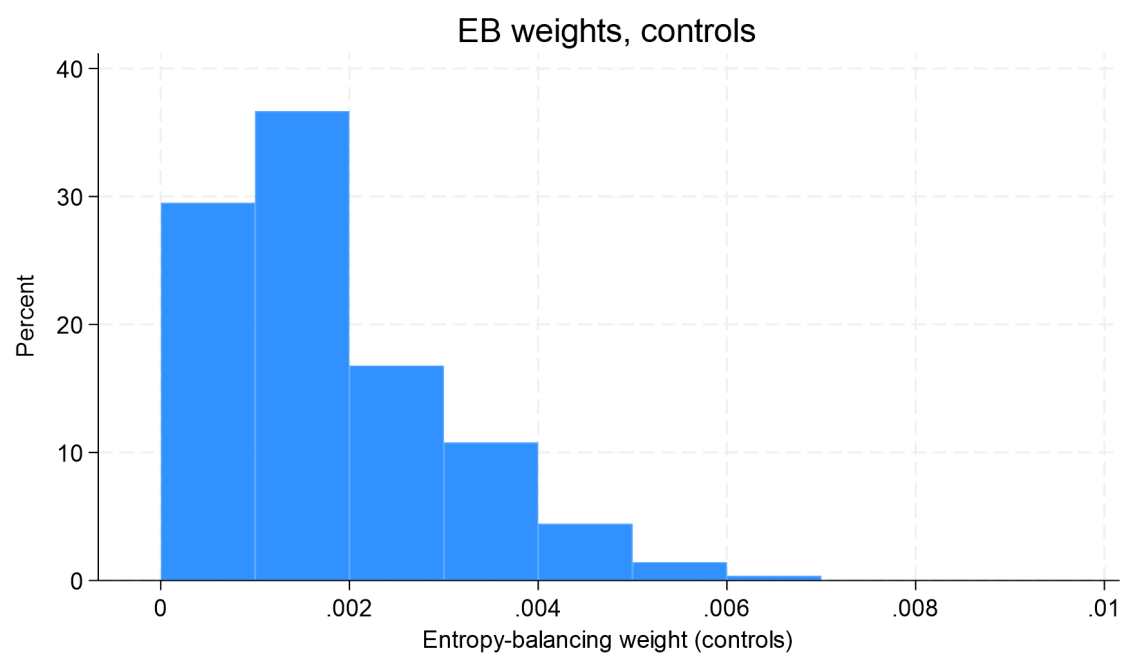

(a) 2010 controls — linear scale

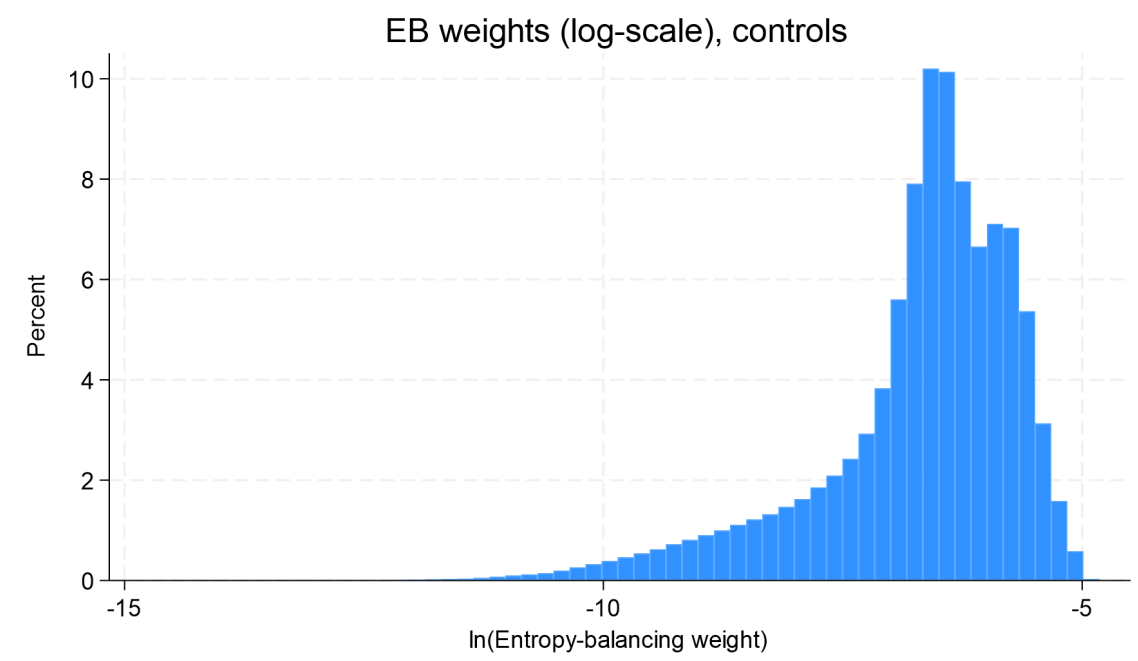

(b) 2010 controls — log scale

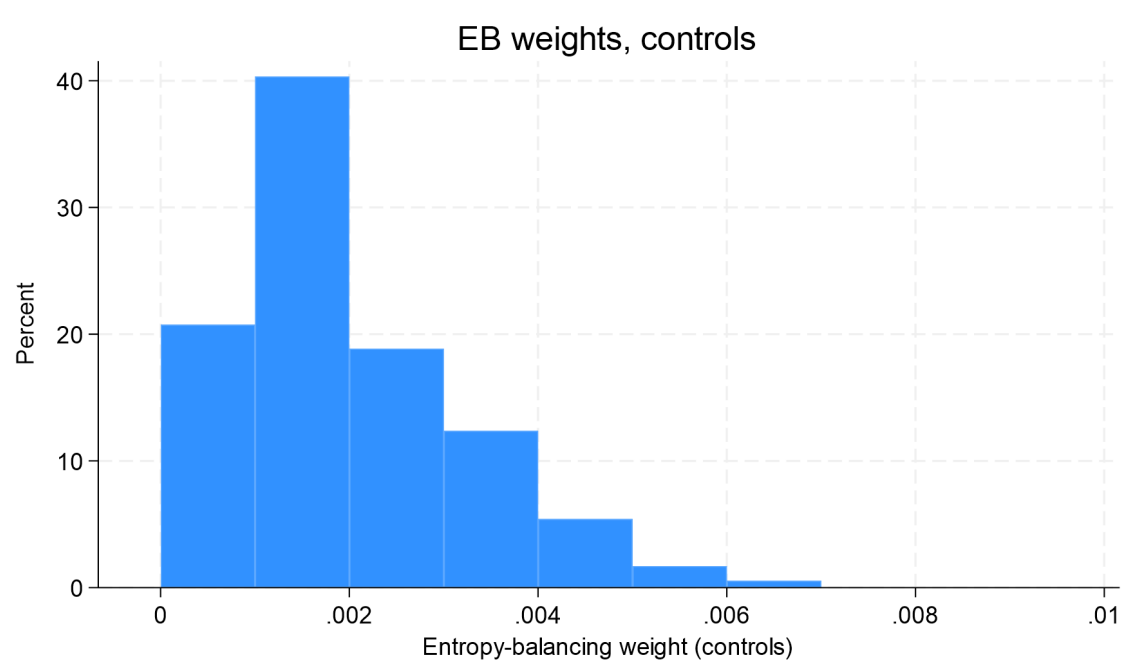

(c) 2018 controls — linear scale

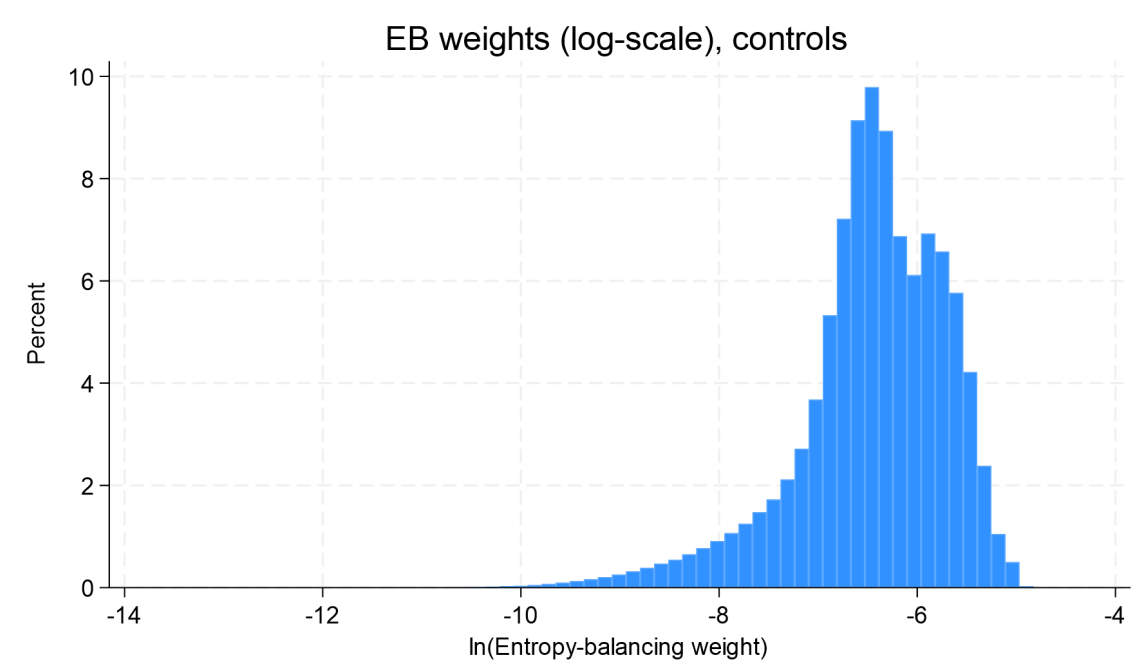

(d) 2018 controls — log scale

Figure 3: Distribution of entropy balancing weights for control units in the restricted samples (2010 and 2018). Each subfigure shows either the raw weights (linear scale) or $\ln(w)$ (log scale).

Notes: Each panel shows the distribution of entropy-balancing weights (w_{eb}) assigned to control units, either on the raw scale (panels (a) and (c)) or the natural-log scale (panels (b) and (d)). Sample: restricted (see Methods); n = 4,367,005 controls in 2010 (panels (a)–(b)) and n = 3,872,512 controls in 2018 (panels (c)–(d)).

Table 15: Entropy-Balancing Weight Diagnostics: Restricted Sample

Metric	2010	2018
Total weight, treated	7,674.00	7,646.00
Total weight, controls	7,674.00	7,646.00
Absolute difference (treated − controls)	0.000000	1.82×10^{-12}
Min. weight (all)	4.08×10^{-7}	0.000002
Max. weight (all)	1.000000	1.000000
Mean weight (all)	0.003508	0.003941
SD weight (all)	0.041792	0.044277
Min. weight (controls)	4.08×10^{-7}	0.000002
Max. weight (controls)	0.009535	0.009234

Continued on next page

Metric	2010	2018
Mean weight (controls)	0.001757	0.001974
SD weight (controls)	0.001256	0.001233
Controls with weight < 0.001	1,287,921.00	801,028.00
Controls with weight < 0.0001	173,774.00	24,691.00
Weight share, top 10 controls	0.000012	0.000011
Weight share, top 100 controls	0.000106	0.000105
Weight share, top 1,000 controls	0.000949	0.000954
Herfindahl index (controls)	3.46×10^{-7}	3.59×10^{-7}
Effective sample size, controls	2,890,722.54	2,786,613.83

Notes. The table reports diagnostics on the entropy-balancing (EB) control weights (w_eb) used in the restricted-sample analyses, for the 2010 and 2018 elections. Treated N = 7,674.00 (2010) and 7,646.00 (2018); by construction, the total weight assigned to controls after entropy balancing equals the total weight of the treated group (adding-up constraint), consistent with EB's exact first-moment matching. Weight (all) summarizes weights across both treated and control units combined (treated units receive weight 1 by construction); weight (controls) restricts to the reweighted control group. The top-10/100/1,000 shares report the proportion of total control weight held by the 10, 100, and 1,000 most heavily weighted control units. The Herfindahl index is the sum of squared normalized control weights, a standard measure of weight concentration, with lower values indicating weight is more evenly distributed across controls. The effective sample size (ESS) for controls is the reciprocal of the Herfindahl index and reflects the number of equally weighted observations that would yield the same estimation precision as the actual weighted control sample.

All weights are non-negative and strictly positive, with small minima (2010: 4.08×10^{-7}; 2018: 1.86×10^{-6}) and maximum control weights well below 0.01 (2010: 0.009535; 2018: 0.009234). These values show that no single control observation receives disproportionate influence, and that the weighted estimator is not driven by a small subset of unusually large weights.

Weight concentration is also extremely low. The largest 1,000 control weights together account for less than 0.10% of the total control weight in both years (2010: 0.09490%; 2018: 0.09539%), and the Herfindahl indices (2010: 3.46×10^{-7}; 2018: 3.59×10^{-7}) further confirm near-complete dispersion of weight mass across the control populations. This pattern demonstrates that the EB algorithm distributes weights broadly rather than concentrating influence in a small region of the empirical support.

The effective sample sizes (ESS) for controls are large (2010: 2.89 million; 2018: 2.79 million), implying that the weighted control groups retain nearly the full informational content of the underlying samples despite reweighting. Taken together, these numerical diagnostics show that entropy balancing performs in a stable and well-behaved manner in both restricted samples.

The weight histograms provide complementary visual evidence. In the restricted 2010 sample, the linear-scale histogram shows that most control weights fall between 0 and roughly 0.004, with a smooth right tail and no extreme values. The log-scale histogram reveals a gradual and continuous decline toward the minimum weight (approximately 4×10^{-7}), with no spikes or mass points at zero. This pattern indicates that optimization converged smoothly and assigned strictly positive weights to all control observations. Together, the numerical and graphical diagnostics confirm that the entropy balancing weights are strictly positive, highly dispersed, and exhibit no signs of instability or overfitting.

Supplementary Note 2: Entropy balancing weight diagnostics for unrestricted sample

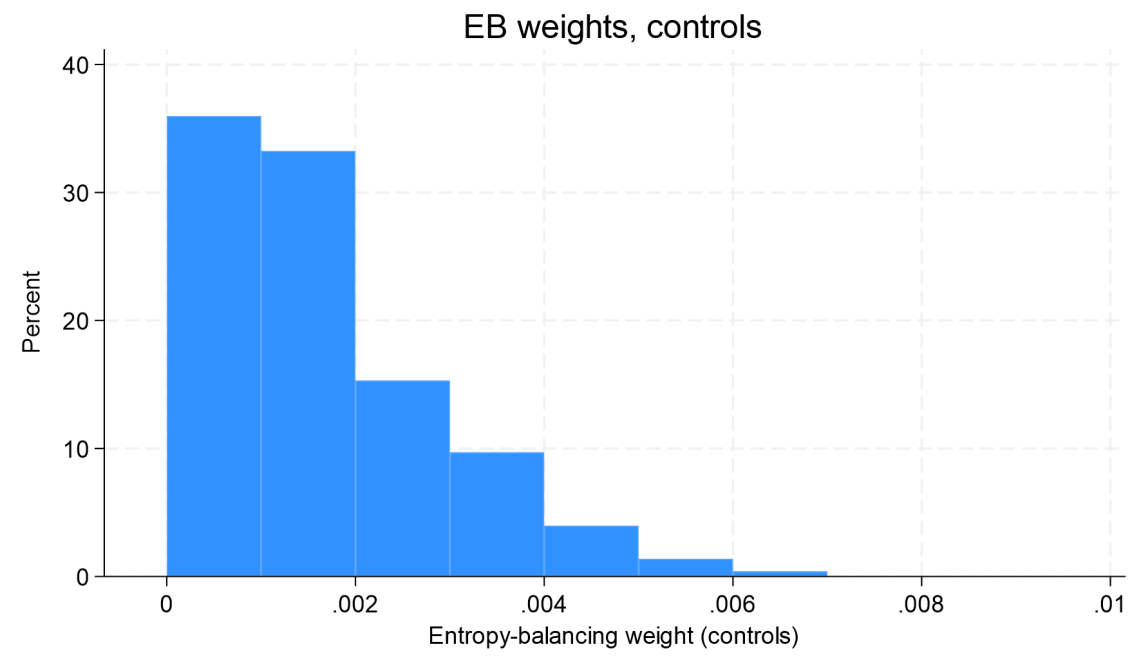

(a) 1994 controls — linear scale

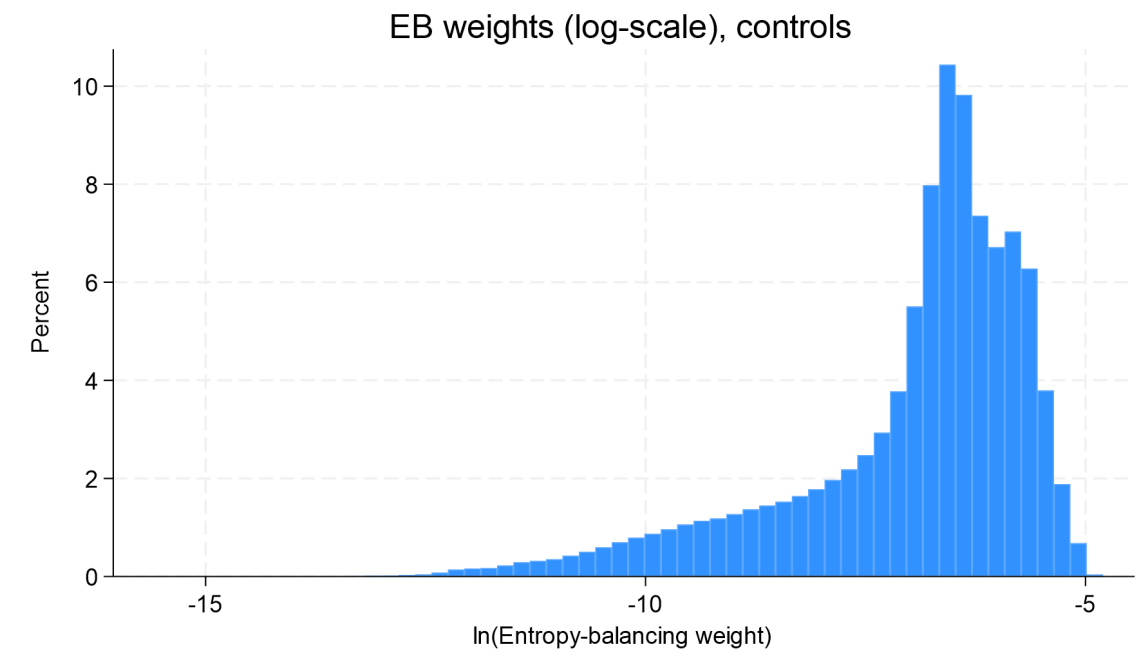

(b) 1994 controls — log scale

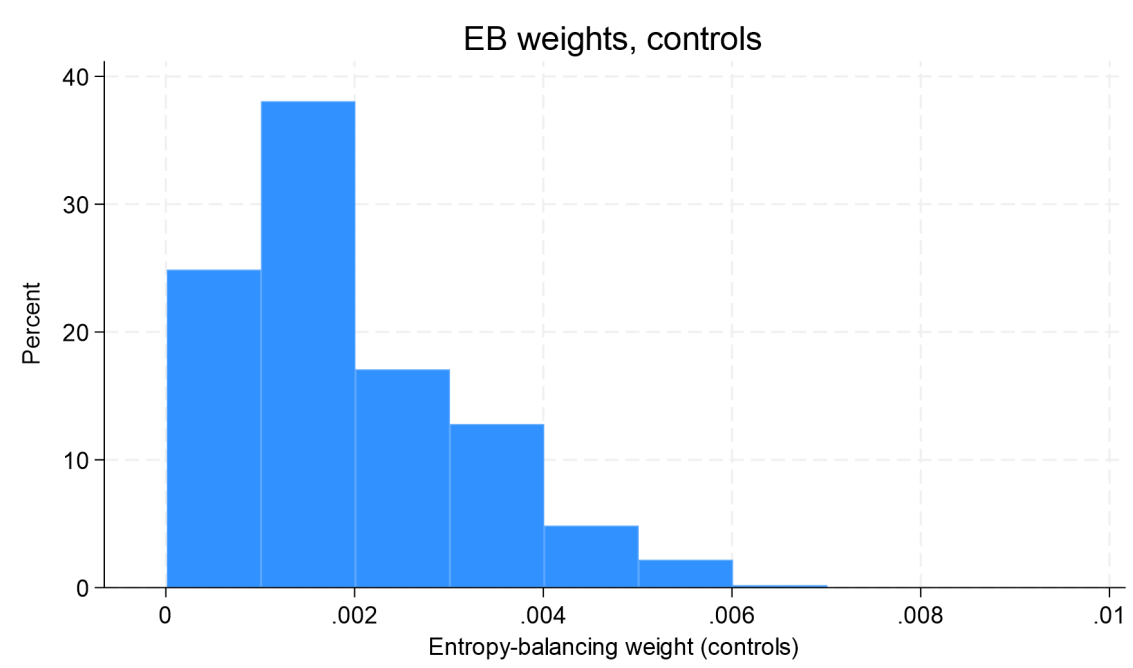

(c) 2010 controls — linear scale

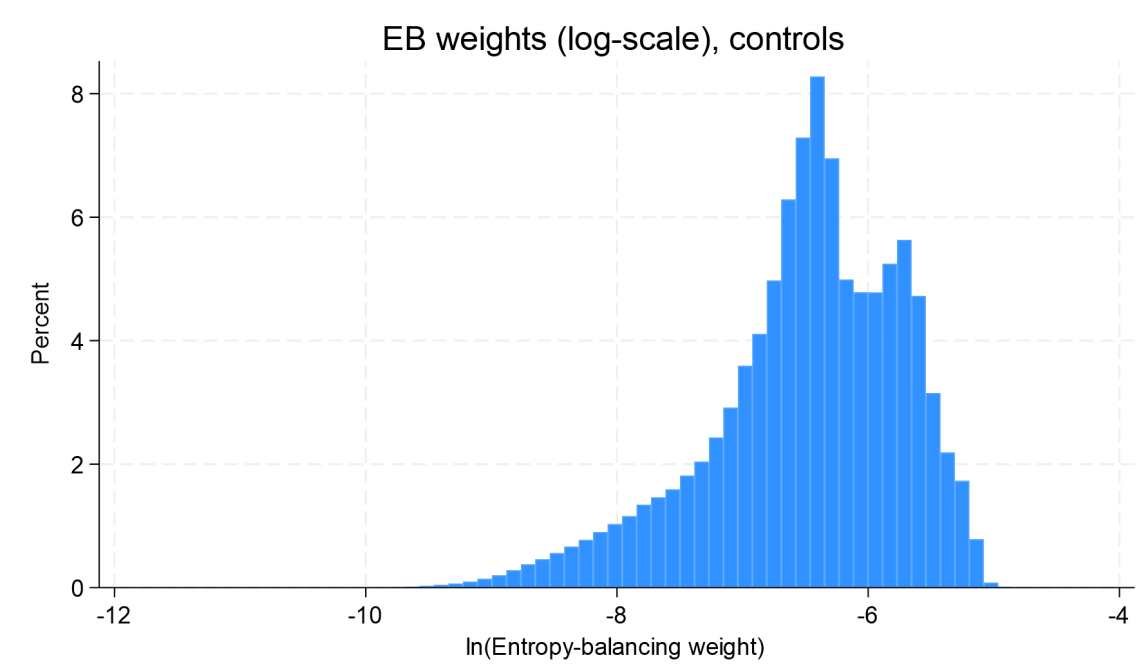

(d) 2010 controls — log scale

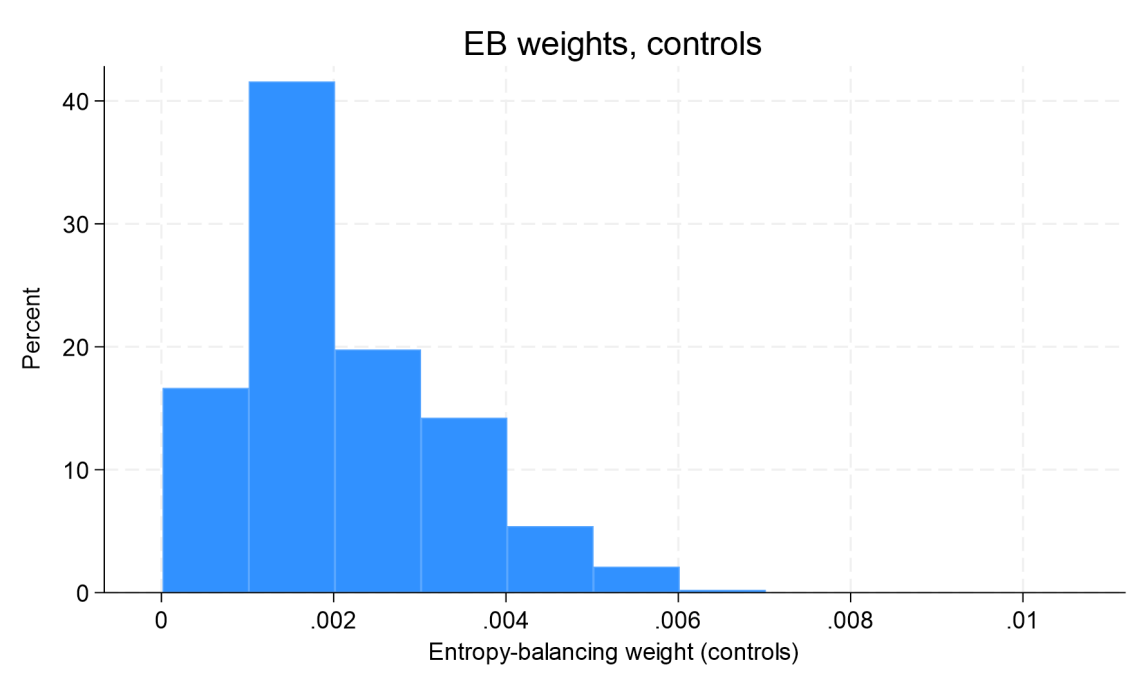

(e) 2018 controls — linear scale

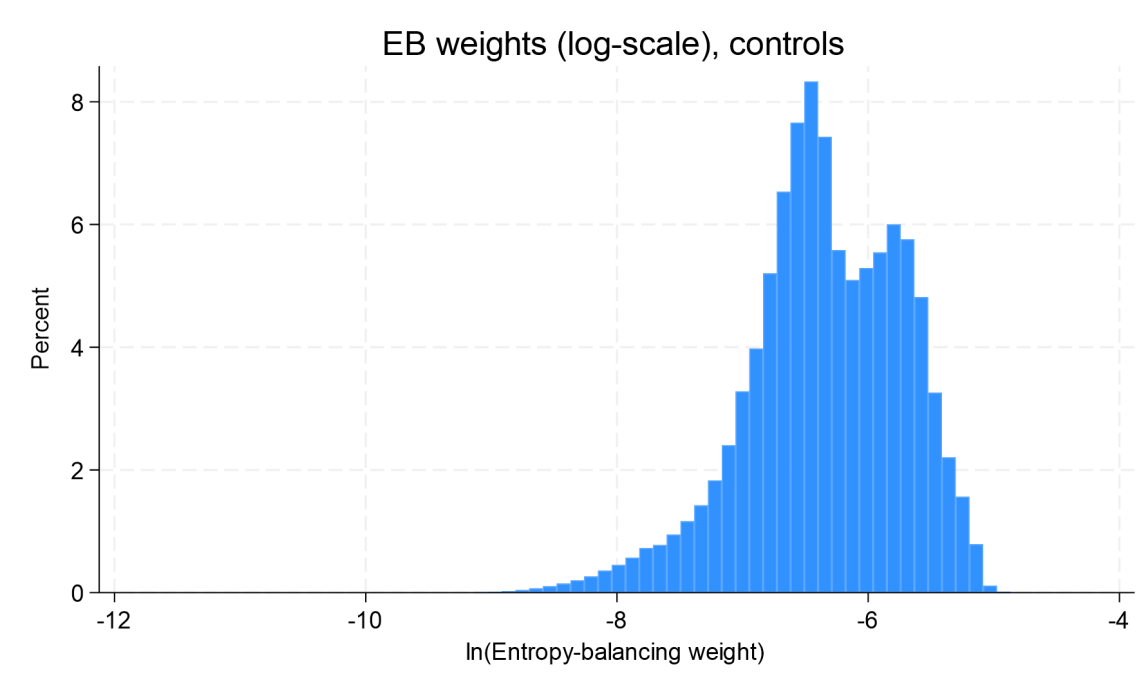

(f) 2018 controls — log scale

Figure 4: Distribution of entropy balancing weights for control units in the unrestricted samples (1994, 2010 and 2018). Each subfigure shows either the raw weights (linear scale) or $\ln(w)$ (log scale).

Notes: Each panel shows the distribution of entropy-balancing weights (w_{eb}) assigned to control units, either on the raw scale (panels (a), (c), and (e)) or the natural-log scale (panels (b), (d), and (f)). Sample: unrestricted (see Methods); n = 5,003,473 controls in 1994 (panels (a)–(b)), n = 6,689,047 controls in 2010 (panels (c)–(d)), and n = 7,024,100 controls in 2018 (panels (e)–(f)).

Table 16: Entropy-Balancing Weight Diagnostics: Unrestricted Sample

Metric	1994	2010	2018
Total weight, treated	8,065.00	12,889.00	14,668.00
Total weight, controls	8,065.00	12,889.00	14,668.00
Absolute difference (treated − controls)	2.73×10^{-12}	0.000000	9.09×10^{-12}
Min. weight (all)	1.40×10^{-7}	0.000010	0.000015
Max. weight (all)	1.000000	1.000000	1.000000
Mean weight (all)	0.003219	0.003846	0.004168
SD weight (all)	0.040040	0.043745	0.045522
Min. weight (controls)	1.40×10^{-7}	0.000010	0.000015
Max. weight (controls)	0.009908	0.009862	0.010706
Mean weight (controls)	0.001612	0.001927	0.002088
SD weight (controls)	0.001293	0.001244	0.001193
Controls with weight < 0.001	1,799,958.00	1,639,949.00	1,132,106.00
Controls with weight < 0.0001	461,987.00	12,456.00	362.00
Weight share, top 10 controls	0.000012	0.000007	0.000007
Weight share, top 100 controls	0.000105	0.000066	0.000060
Weight share, top 1,000 controls	0.000932	0.000570	0.000517
Herfindahl index (controls)	3.28×10^{-7}	2.12×10^{-7}	1.89×10^{-7}
Effective sample size, controls	3,045,231.92	4,720,843.93	5,296,415.83

Notes. The table reports diagnostics on the entropy-balancing (EB) control weights (w_eb) used in the unrestricted-sample analyses, for the 1994 (pre-treatment placebo), 2010, and 2018 elections. Treated N = 8,065.00 (1994), 12,889.00 (2010), and 14,668.00 (2018); by construction, the total weight assigned to controls after entropy balancing equals the total weight of the treated group (adding-up constraint), consistent with EB's exact first-moment matching. Weight (all) summarizes weights across both treated and control units combined (treated units receive weight 1 by construction); weight (controls) restricts to the reweighted control group. The top-10/100/1,000 shares report the proportion of total control weight held by the 10, 100, and 1,000 most heavily weighted control units. The Herfindahl index is the sum of squared normalized control weights, a standard measure of weight concentration, with lower values indicating weight is more evenly distributed across controls. The effective sample size (ESS) for controls is the reciprocal of the Herfindahl index and reflects the number of equally weighted observations that would yield the same estimation precision as the actual weighted control sample.

Across all election years, the total weights assigned to treated and control units are well matched (1994: 8,065 vs. 8,065; 2010: 12,889 vs. 12,889; 2018: 14,668 vs. 14,668), with absolute differences at or below numerical precision (1994: $\Delta = 2.73 \times 10^{-12}$; 2010: $\Delta = 0$; 2018: $\Delta = 9.09 \times 10^{-12}$). The exact zero difference in the 2010 sample indicates that the balancing constraints were satisfied to precision.

All weights are non-negative and strictly positive, with small minima across all years (1994: 1.40×10^{-7}; 2010: 9.84×10^{-6}; 2018: 1.50×10^{-5}) and maximum control weights at or near the 0.01 threshold (1994: 0.009908; 2010: 0.009862; 2018: 0.010706). These values confirm that no single control observation receives disproportionate influence, and that the weighted estimator is not driven by a small subset of unusually large weights.

Weight concentration is very low across all three samples. The largest 1,000 control weights together account for less than 0.10% of the total control weight in every year (1994: 0.093%; 2010: 0.057%; 2018: 0.052%), and the Herfindahl indices decline monotonically from the placebo year to 2018 (1994: 3.28×10^{-7}; 2010: 2.12×10^{-7}; 2018: 1.89×10^{-7}), reflecting increasing weight dispersion as the treated and control populations grow.

The effective sample sizes (ESS) for controls are large and increasing across years (1994: 3.05 million;

2010: 4.72 million; 2018: 5.30 million), implying that the weighted control groups retain the vast majority of the informational content of the underlying samples despite reweighting. The diagnostics confirm that entropy balancing performs in a stable and well-behaved manner across all unrestricted samples, including the pre-treatment placebo year.

Supplementary Note 3: Survey Analyses

Survey Items

Below we reproduce the exact wording and response options for the survey items used in the analysis.

Support Received After the Tsunami

Question(s):

What support, if any, did you receive from the following actors after the 2004 tsunami?

- Friends and acquaintances
- My family
- Public authorities

Response scale (1–6):

1.	Very good support
2.	Fairly good support
3.	Neither good nor poor support
4.	Fairly poor support
5.	Very poor support
6.	Had no contact

Each survey item was used to construct two variables shown Figure 3 of the main text. First, response options 1–5 were reverse-coded so that higher values indicate better support; these variables are displayed in Panel A of Figure 3 of the main text. Second, responses coded as 6 ("Had no contact") were transformed into an indicator capturing the share of respondents reporting no contact, which forms the basis for Panel B. This question was administered only to tsunami survivors. Supplementary Figure 5 shows the full distribution of responses to each item by exposure group.

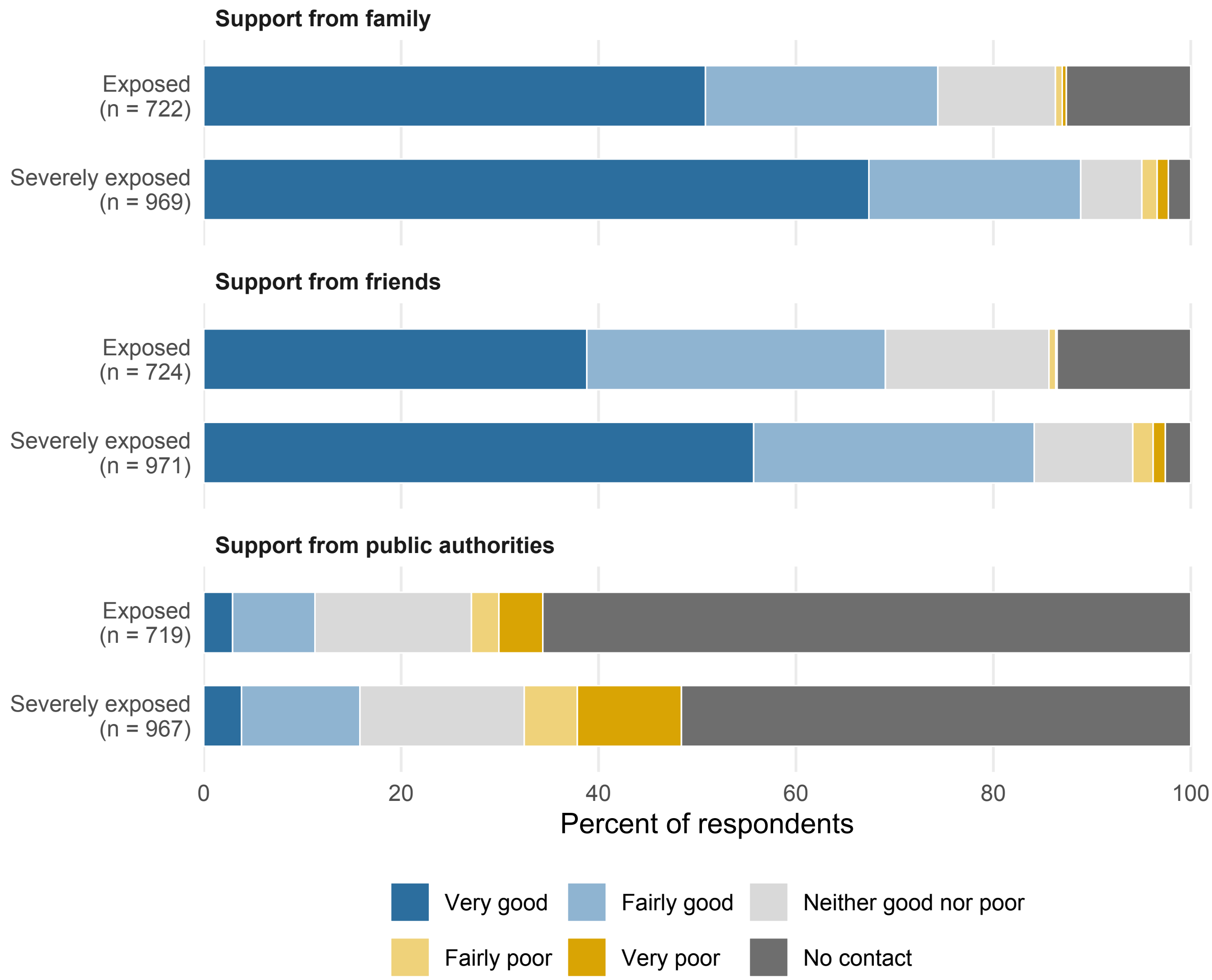

Notes: Percentage of exposed respondents in each response category, by support source and exposure group. The five rating categories underlie the means in Figure 3, Panel A, of the main text, and the "No contact" category underlies the shares in Panel B. The number of respondents is given in each bar label. No statistical tests were performed.

Figure 5: Distribution of responses to the question on support received after the tsunami

Government Handling of Crises

Question(s):

How do you think responsible public authorities and political leaders have handled the following societal accidents, failures, crises, or disasters?

- The Estonia disaster
- Storm Gudrun
- The Tsunami disaster
- The 2008 financial crisis
- The Drottninggatan terrorist attack
- The 2018 forest and grassland fires

- The COVID-19 pandemic
- The 2022 energy crisis
- The 2023 NATO–Turkey crisis

Response scale (1–6):

1.	Very good
2.	Fairly good
3.	Neither good nor poor
4.	Fairly poor
5.	Very poor
6.	Don't know

Each survey item was used to construct three variables presented in Figure 3 of the main text, Panels C–E. First, response options 1–5 were reverse-coded so that higher values indicate better government handling. Second, responses coded as 6 ("Don't know") were recoded as missing. Third, evaluations of "The tsunami disaster" were used to measure perceptions of the government's handling of the tsunami specifically (Panel C); evaluations of "Storm Gudrun" and "The 2018 forest and grassland fires" were combined to measure handling of natural disasters (Panel D); and evaluations of all remaining crises were used to measure handling of other types of crises (Panel E). These questions were administered to both tsunami survivors and the control group.

Vote Choice in the Parliamentary Election 2022

Question(s):

Which party did you vote for in the 2022 parliamentary election?

Response scale (Swedish party name and abbreviation in parenthesis):

- The Left Party (Vänsterpartiet, V)
- The Social Democratic Party (Socialdemokraterna, S)
- The Green Party (Miljöpartiet, MP)
- The Centre Party (Centerpartiet, C)
- The Liberal Party (Liberalerna, L)
- The Moderate Party (Moderaterna, M)
- The Christian Democrats (Kristdemokraterna, KD)
- The Sweden Democrats (Sverigedemokraterna, SD)

The question was used to construct a vote choice variable, presented in Figure 3 of the main text, Panel F. The question was administered to both tsunami survivors and the control group.

Trust in Crisis Management Capacity

Question(s):

More specifically, how much trust do you have in the ability of politicians, public authorities, and ordinary people to manage a serious societal accident, failure, crisis, or disaster in Sweden?

- Politicians
- Public authorities

- Ordinary people's own ability

Response scale (1–6):

1.	Very high trust
2.	Fairly high trust
3.	Neither high nor low trust
4.	Fairly low trust
5.	Very low trust
6.	No opinion

Response options 1–5 were reverse-coded so that higher values indicate greater trust. Response option 6 ("No opinion") was coded as missing. The measures are used in Table 18 in this note.

Tsunami Exposure

Respondents in the tsunami group were asked a set of factual questions about their experiences during the 2004 Indian Ocean tsunami. The severely exposed category is based on six items capturing the most acute and life-threatening dimensions of exposure: wave impact, perceived danger of death, severe physical injury, and serious harm or loss within the family. Below we list the survey questions used to construct this variable, along with their response scales.

Question(s):

Were you yourself caught by the waves?

Response scale (1–3):

1.	No
2.	Yes, completely, partly, or nearly
3.	Yes, pulled completely under water

To what extent did you believe you were in danger of dying?

Response scale (1–3):

1.	Believed there was no danger of dying
2.	Believed there was some danger of dying
3.	Believed there was great danger of dying

Did you receive any physical injuries? Were you admitted to hospital or a similar facility?

Response scale (1–3):

1.	No
2.	Injured but not admitted
3.	Injured and admitted to hospital or similar facility

If you saw anyone being caught by the waves, what was your relation to this person or these persons?

Response scale (1–3):

1.	Saw no one caught by the waves
2.	No relationship to those caught
3.	Family member(s) or close friend(s)

Was a family member or close friend injured and in need of help?
Response scale (1–2):

1.	Yes
2.	No

Did you lose a family member or close friend in the disaster?
Response scale (1–2):

1.	Yes
2.	No

Construction of the severely exposed variable. Respondents were classified as severely exposed if they selected any of the following response options: being caught by the waves (2 or 3), perceiving some or great danger of dying (2 or 3), sustaining injuries requiring hospital admission (3), witnessing a family member or close friend caught by the waves (3), having a family member or close friend injured and needing help (1), or losing a family member or close friend (1).
Demographic and socioeconomic control variables. The variables measuring age, sex, income and education were extracted from governmental registries.

Table 17: Summary Statistics, Survey Sample

Variable	Mean	SD	Min	Max	N
Panel A: Government Crisis Response Evaluations					
Tsunami handling	1.898	1.283	0	4	3,257
Natural disaster handling	2.401	0.839	0	4	3,221
General crisis handling	2.017	0.700	0	4	3,625
Panel B: Social Support					
Support from family	3.502	0.786	0	4	1,578
Support from friends	3.328	0.832	0	4	1,572
Support from authorities	1.933	1.191	0	4	715
Panel C: No Contact with Support Source (share)					
No contact: family	0.067	0.250	0	1	1,691
No contact: friends	0.073	0.259	0	1	1,695
No contact: authorities	0.576	0.494	0	1	1,686
Panel D: Party Vote (2022)					
Left Party (V)	0.063	0.243	0	1	3,198
Social Democrats (S)	0.319	0.466	0	1	3,198
Green Party (MP)	0.065	0.247	0	1	3,198
Centre Party (C)	0.078	0.268	0	1	3,198
Liberals (L)	0.064	0.244	0	1	3,198
Moderates (M)	0.218	0.413	0	1	3,198
Christian Democrats (KD)	0.040	0.195	0	1	3,198
Sweden Democrats (SD)	0.132	0.339	0	1	3,198
Panel E: Exposure and Controls					
Tsunami exposure	1.568	0.495	1	2	1,726
Age	58.67	14.73	29	92	3,669
Sex	1.501	0.500	1	2	3,669
Education (tertiary)	0.518	0.500	0	1	3,669
Income (SEK, thousands)	4,484	2,815	0	43,277	3,669

Notes. Descriptive statistics for the 2023 survey sample. Crisis response evaluations range from 0 (very poor) to 4 (very good). Social support ranges from 0 (no support) to 4 (very much support). No contact is coded 1 if the respondent reported having no contact with the respective support source following the disaster. Party vote variables are indicator variables for self-reported vote choice in the 2022 general election. N varies across items due to item non-response.

Table 18: Tsunami exposure and ratings of crisis handling

	Estimate	95% CI	t	p
Tsunami				
Exposed	-0.174	-0.294 to -0.054	-2.84	0.005
Severely exposed	-0.643	-0.753 to -0.532	-11.41	<0.001
Difference, severely exposed vs. exposed	-0.469	-0.609 to -0.329	-6.57	<0.001
Age	-0.002	-0.006 to 0.001	-1.42	0.155
Sex (female)	0.135	0.040 to 0.229	2.80	0.005
Education	0.097	0.001 to 0.194	1.97	0.049
Income	0.000	0.000 to 0.000	0.60	0.551
Intercept	1.985	1.713 to 2.256	14.33	<0.001
N, Adjusted R^2	N = 3,257; Adjusted R^2 = 0.050			
Natural disasters (Gudrun storm, wildfires)				
Exposed	-0.105	-0.189 to -0.021	-2.46	0.014
Severely exposed	-0.109	-0.182 to -0.035	-2.89	0.004
Difference, severely exposed vs. exposed	-0.003	-0.098 to 0.091	-0.07	0.946
Age	-0.002	-0.004 to 0.000	-1.65	0.099
Sex (female)	0.078	0.013 to 0.142	2.36	0.018
Education	0.091	0.025 to 0.158	2.68	0.007
Income	0.000	0.000 to 0.000	1.95	0.052
Intercept	2.347	2.156 to 2.539	24.02	<0.001
N, Adjusted R^2	N = 3,221; Adjusted R^2 = 0.012			
Other crises (Estonia ferry disaster, financial crisis, terrorism, COVID-19, energy crisis, NATO)				
Exposed	0.007	-0.056 to 0.070	0.21	0.833
Severely exposed	-0.044	-0.102 to 0.015	-1.46	0.143
Difference, severely exposed vs. exposed	-0.050	-0.122 to 0.022	-1.37	0.170
Age	0.001	-0.001 to 0.003	1.31	0.189
Sex (female)	0.084	0.034 to 0.134	3.30	<0.001
Education	0.168	0.116 to 0.220	6.35	<0.001
Income	0.000	0.000 to 0.000	6.22	<0.001
Intercept	1.602	1.454 to 1.750	21.22	<0.001
N, Adjusted R^2	N = 3,625; Adjusted R^2 = 0.034			

Notes. Survey-reported ratings of how well Swedish society handled the tsunami and, for comparison, other disasters and crises, regressed on tsunami exposure group (Control, i.e. unexposed, as the reference category), controlling for age, sex, education, and income, with survey weights applied. Each panel's block reports the exposure coefficient at each exposure level (Exposed, Severely exposed) and their difference, followed by the coefficients on the control variables. CI, confidence interval. t statistics and p-values are from two-sided t-tests of the null hypothesis that the estimate equals zero; no adjustment for multiple comparisons was made. N and adjusted R^2 are reported per item model.

Table 19: Tsunami exposure and trust in future crisis handling

	Estimate	95% CI	t	p
Politicians				
Exposed	-0.014	-0.106 to 0.078	-0.30	0.762
Severely exposed	-0.128	-0.212 to -0.045	-3.02	0.003
Difference, severely exposed vs. exposed	-0.114	-0.219 to -0.010	-2.14	0.032
Age	0.009	0.007 to 0.012	7.31	<0.001
Sex (female)	0.220	0.147 to 0.292	5.91	<0.001
Education	0.096	0.021 to 0.171	2.52	0.012
Income	0.000	0.000 to 0.000	2.48	0.013
Intercept	1.064	0.850 to 1.279	9.72	<0.001
N, Adjusted R^2	N = 3,591; Adjusted R^2 = 0.033			
Authorities				
Exposed	0.010	-0.082 to 0.102	0.21	0.831
Severely exposed	-0.086	-0.169 to -0.003	-2.03	0.043
Difference, severely exposed vs. exposed	-0.096	-0.200 to 0.008	-1.82	0.069
Age	0.002	0.000 to 0.005	1.61	0.107
Sex (female)	0.142	0.069 to 0.215	3.82	<0.001
Education	0.232	0.156 to 0.308	5.99	<0.001
Income	0.000	0.000 to 0.000	3.45	<0.001
Intercept	1.976	1.753 to 2.198	17.42	<0.001
N, Adjusted R^2	N = 3,588; Adjusted R^2 = 0.025			
The public				
Exposed	0.073	-0.012 to 0.157	1.69	0.091
Severely exposed	0.050	-0.028 to 0.128	1.26	0.207
Difference, severely exposed vs. exposed	-0.023	-0.117 to 0.072	-0.48	0.635
Age	0.010	0.008 to 0.012	8.41	<0.001
Sex (female)	0.030	-0.039 to 0.099	0.86	0.392
Education	0.036	-0.035 to 0.106	0.99	0.322
Income	0.000	0.000 to 0.000	2.39	0.017
Intercept	1.715	1.508 to 1.923	16.20	<0.001
N, Adjusted R^2	N = 3,564; Adjusted R^2 = 0.027			

Notes. Survey-reported trust in different actors' handling of a future crisis , regressed on tsunami exposure group (Control, i.e. unexposed, as the reference category), controlling for age, sex, education, and income, with survey weights applied. Each panel's block reports the exposure coefficient at each exposure level (Exposed, Severely exposed) and their difference, followed by the coefficients on the control variables. CI, confidence interval. t statistics and p-values are from two-sided t-tests of the null hypothesis that the estimate equals zero; no adjustment for multiple comparisons was made. N and adjusted R^2 are reported per item model.

Table 20: Tsunami exposure and vote choice

	Estimate	95% CI	t	p
V				
Exposed	2.494	-0.225 to 5.213	1.80	0.072
Severely exposed	0.309	-1.948 to 2.567	0.27	0.788
Difference, severely exposed vs. exposed	-2.184	-5.238 to 0.869	-1.40	0.161
Age	-0.155	-0.223 to -0.087	-4.46	<0.001
Sex (female)	-0.362	-2.337 to 1.614	-0.36	0.720
Education	3.712	1.661 to 5.764	3.55	<0.001
Income	-0.001	-0.001 to 0.000	-4.10	<0.001
Intercept	16.922	11.158 to 22.686	5.76	<0.001
N, Adjusted R^2	N = 3,198; Adjusted R^2 = 0.020			
S				
Exposed	-5.619	-9.911 to -1.326	-2.57	0.010
Severely exposed	-5.372	-9.306 to -1.438	-2.68	0.007
Difference, severely exposed vs. exposed	0.247	-4.519 to 5.013	0.10	0.919
Age	0.297	0.183 to 0.412	5.08	<0.001
Sex (female)	6.096	2.619 to 9.574	3.44	<0.001
Education	1.165	-2.350 to 4.681	0.65	0.516
Income	-0.001	-0.002 to -0.001	-4.20	<0.001
Intercept	12.660	3.224 to 22.097	2.63	0.009
N, Adjusted R^2	N = 3,198; Adjusted R^2 = 0.020			
MP				
Exposed	1.446	-1.005 to 3.897	1.16	0.247
Severely exposed	-0.761	-2.823 to 1.302	-0.72	0.470
Difference, severely exposed vs. exposed	-2.207	-4.924 to 0.510	-1.59	0.111
Age	-0.097	-0.160 to -0.034	-3.02	0.003
Sex (female)	2.050	0.226 to 3.873	2.20	0.028
Education	6.282	4.389 to 8.176	6.51	<0.001
Income	0.000	-0.001 to 0.000	-1.27	0.205
Intercept	6.405	1.188 to 11.623	2.41	0.016
N, Adjusted R^2	N = 3,198; Adjusted R^2 = 0.026			
C				
Exposed	-0.267	-2.918 to 2.385	-0.20	0.844
Severely exposed	-2.955	-5.196 to -0.714	-2.59	0.010
Difference, severely exposed vs. exposed	-2.688	-5.497 to 0.121	-1.88	0.061
Age	-0.040	-0.107 to 0.027	-1.17	0.241
Sex (female)	3.033	0.962 to 5.104	2.87	0.004
Education	4.012	1.944 to 6.080	3.80	<0.001
Income	0.001	0.000 to 0.001	2.79	0.005

Continued on next page

	Estimate	95% CI	t	p
Intercept	1.934	-3.507 to 7.375	0.70	0.486
N, Adjusted R^2	N = 3,198; Adjusted R^2 = 0.015			
L				
Exposed	1.268	-1.028 to 3.565	1.08	0.279
Severely exposed	1.834	-0.346 to 4.013	1.65	0.099
Difference, severely exposed vs. exposed	0.566	-2.169 to 3.301	0.41	0.685
Age	-0.028	-0.088 to 0.032	-0.91	0.361
Sex (female)	-1.427	-3.340 to 0.486	-1.46	0.144
Education	4.881	2.979 to 6.783	5.03	<0.001
Income	0.001	0.000 to 0.001	3.12	0.002
Intercept	3.491	-2.241 to 9.222	1.19	0.233
N, Adjusted R^2	N = 3,198; Adjusted R^2 = 0.021			
M				
Exposed	1.309	-2.523 to 5.140	0.67	0.503
Severely exposed	7.949	4.264 to 11.633	4.23	<0.001
Difference, severely exposed vs. exposed	6.640	2.135 to 11.144	2.89	0.004
Age	-0.003	-0.107 to 0.101	-0.05	0.959
Sex (female)	0.627	-2.456 to 3.710	0.40	0.690
Education	-6.174	-9.429 to -2.918	-3.72	<0.001
Income	0.002	0.001 to 0.003	6.44	<0.001
Intercept	12.699	3.573 to 21.825	2.73	0.006
N, Adjusted R^2	N = 3,198; Adjusted R^2 = 0.024			
KD				
Exposed	-0.624	-2.353 to 1.104	-0.71	0.479
Severely exposed	0.314	-1.418 to 2.047	0.36	0.722
Difference, severely exposed vs. exposed	0.939	-1.089 to 2.966	0.91	0.364
Age	0.002	-0.050 to 0.055	0.08	0.933
Sex (female)	-1.123	-2.567 to 0.322	-1.52	0.128
Education	-0.554	-2.114 to 1.007	-0.70	0.487
Income	0.000	0.000 to 0.000	0.71	0.476
Intercept	5.383	0.906 to 9.860	2.36	0.018
N, Adjusted R^2	N = 3,198; Adjusted R^2 = -0.000			
SD				
Exposed	0.701	-2.520 to 3.923	0.43	0.670
Severely exposed	-0.287	-3.151 to 2.576	-0.20	0.844
Difference, severely exposed vs. exposed	-0.989	-4.567 to 2.589	-0.54	0.588
Age	0.099	0.015 to 0.183	2.32	0.021
Sex (female)	-7.256	-9.783 to -4.729	-5.63	<0.001
Education	-11.717	-14.322 to -9.111	-8.82	<0.001
Income	-0.001	-0.001 to -0.001	-5.47	<0.001

Continued on next page

	Estimate	95% CI	t	p
Intercept	29.256	21.828 to 36.683	7.72	<0.001
N, Adjusted R^2	N = 3,198; Adjusted R^2 = 0.060			

Notes. Survey-reported vote choice in the national parliamentary election (party vote share, in percentage points) regressed on tsunami exposure group (Control, i.e. unexposed, as the reference category), controlling for age, sex, education, and income, with survey weights applied. Each party's block reports the exposure coefficient at each exposure level (Exposed, Severely exposed) and their difference, followed by the coefficients on the control variables. CI, confidence interval. t statistics and p-values are from two-sided t-tests of the null hypothesis that the estimate equals zero; no adjustment for multiple comparisons was made. N and adjusted R^2 are reported per party model.

Table 21: Covariate-Adjusted Effects of Tsunami Exposure Severity on Social Support and Contact with Support Sources

	Estimate	95% CI	t	p
Panel A: Social Support				
Support from family				
Severely exposed vs. exposed	0.156	0.071 to 0.242	3.58	<0.001
Age	0.006	0.003 to 0.009	3.64	<0.001
Sex (female)	0.029	-0.059 to 0.117	0.65	0.515
Education	0.056	-0.034 to 0.146	1.22	0.221
Income	0.000	0.000 to 0.000	3.67	<0.001
Intercept	2.883	2.615 to 3.151	21.10	<0.001
N, Adjusted R^2	N = 1,578; Adjusted R^2 = 0.022			
Support from friends				
Severely exposed vs. exposed	0.159	0.071 to 0.247	3.54	<0.001
Age	0.006	0.003 to 0.010	3.62	<0.001
Sex (female)	0.098	0.008 to 0.189	2.13	0.034
Education	0.020	-0.076 to 0.116	0.41	0.681
Income	0.000	0.000 to 0.000	5.59	<0.001
Intercept	2.561	2.296 to 2.826	18.95	<0.001
N, Adjusted R^2	N = 1,572; Adjusted R^2 = 0.029			
Support from authorities				
Severely exposed vs. exposed	-0.180	-0.371 to 0.011	-1.85	0.065
Age	0.013	0.006 to 0.020	3.61	<0.001
Sex (female)	0.057	-0.138 to 0.251	0.57	0.567
Education	0.162	-0.035 to 0.358	1.61	0.107
Income	0.000	0.000 to 0.000	-0.93	0.354
Intercept	1.187	0.582 to 1.793	3.85	<0.001
N, Adjusted R^2	N = 715; Adjusted R^2 = 0.026			
Panel B: No Contact with Support Source (share)				
No contact: family				
Severely exposed vs. exposed	-0.087	-0.112 to -0.062	-6.74	<0.001
Age	0.002	0.001 to 0.003	5.14	<0.001
Sex (female)	0.009	-0.014 to 0.033	0.78	0.434
Education	0.021	-0.003 to 0.044	1.73	0.083
Income	0.000	0.000 to 0.000	-2.48	0.013
Intercept	0.000	-0.055 to 0.056	0.02	0.986
N, Adjusted R^2	N = 1,691; Adjusted R^2 = 0.052			
No contact: friends				
Severely exposed vs. exposed	-0.092	-0.118 to -0.066	-7.03	<0.001
Age	0.002	0.001 to 0.003	5.51	<0.001
Sex (female)	0.024	0.000 to 0.047	1.99	0.047
Education	0.012	-0.012 to 0.036	0.99	0.323
Income	0.000	0.000 to 0.000	-3.28	0.001

Continued on next page

	Estimate	95% CI	t	p
Intercept	-0.020	-0.077 to 0.036	-0.70	0.484
N, Adjusted R^2	N = 1,695; Adjusted R^2 = 0.064			
No contact: authorities				
Severely exposed vs. exposed	-0.143	-0.192 to -0.093	-5.67	<0.001
Age	-0.001	-0.002 to 0.001	-0.83	0.409
Sex (female)	0.089	0.039 to 0.140	3.45	<0.001
Education	0.087	0.035 to 0.139	3.27	0.001
Income	0.000	0.000 to 0.000	-0.12	0.907
Intercept	0.527	0.377 to 0.677	6.88	<0.001
N, Adjusted R^2	N = 1,686; Adjusted R^2 = 0.035			

Notes. Estimates are from OLS regressions with survey calibration weights, adjusted for age, sex, education, and income. Because the underlying items (perceived social support and contact with support sources) were administered only to tsunami-exposed respondents, no control-group comparison is available; estimates reflect the difference between severely exposed and exposed survivors only. Corresponding unadjusted group means are shown in Figure 3, Panels A and B.

References

1. SOM Institute, University of Gothenburg. National SOM Survey Cumulative Dataset 1986–2022 version 13 (2024).
2. Fischer, E. Toten ihre Namen zurückgeben https://www.bmi.gv.at/magazinfiles/2006/05_06/files/tsunami.pdf (2026).
3. SPF Chancellerie du Premier Ministre. Tsunami : suivi de la catastrophe naturelle en Asie du Sud-Est https://news.belgium.be/fr/tsunami-suivi-de-la-catastrophe-naturelle-en-asie-du-sud-est (2026).
4. Novi list. Deset godina od najrazornijeg tsunamija u novijoj povijesti https://www.novilist.hr/novosti/svijet/deset-godina-od-najrazornijeg-tsunamija-u-novijoj-povijesti/ (2026).
5. IDNES.cz. SPECIÁL: Před rokem zabíjely vlny tsunami https://www.idnes.cz/zpravy/domaci/special-pred-rokem-zabijely-vlny-tsunami.A051202_133951_prilohy_miz (2026).
6. Heir, T., Rosendal, S. & Bergh-Johannesson, K. e. a. Tsunami-Affected Scandinavian Tourists: Disaster Exposure and Post-Traumatic Stress Symptoms. Nordic Journal of Psychiatry 65: 9–15 (2011).
7. The Baltic Times. Three Estonians believed dead as a result of tsunami https://www.baltictimes.com/news/articles/11683/ (2026).
8. Safety Investigation Authority, Finland. A2/2004Y The Natural Disaster in Asia on 26 December, 2004 https://www.turvallisuustutkinta.fi/en/investigations/investigation-reports/a2-2004y-the-natural-disaster-in-asia-on-26-december-2004/ (2026).
9. Le Parisien. La renaissance des survivants du tsunami https://www.leparisien.fr/archives/la-renaissance-des-survivants-du-tsunami-26-12-2014-4400201.php (2026).
10. Deutsche Welle. Tsunami commemorations https://www.dw.com/en/germany-joins-in-commemorating-asian-tsunami-victims/a-5059276 (2026).

11. Carolan, M. Tsunami victims died of drowning https://www.irishtimes.com/news/tsunami-victims-died-of-drowning-1.490640 (2026).
12. Il Sole 24 ORE. Tsunami, dieci anni fa l'onda che sconvolse il mondo https://st.ilsole24ore.com/art/notizie/2014-12-26/26122004-dieci-10-anni-fa-tsunami-che-sconvolse-mondo-104459.shtml?uuid=ABMjHYVC&refresh_ce=1 (2026).
13. The Guardian. First Swedish Bodies Flown Home https://www.theguardian.com/world/2005/jan/04/tsunami2004 (2026).
14. Times of Malta. 12 years after Asia tsunami, 400 bodies unidentified in Thailand https://timesofmalta.com/article/12-years-after-Asia-tsunami-400-bodies-unidentified-in-Thailand.634937 (2026).
15. Stichting Tsunami Monument. Tsunami-Monument.Nl https://www.tsunami-monument.nl/ (2026).
16. Faber, C. "Czarne i monstrualne fale". 18 lat temu tsunami zaskoczyło wszystkich RMF 24. https://www.rmf24.pl/fakty/swiat/news-czarne-i-monstrualne-fale-18-lat-temu-tsunami-zaskoczylo-wsz,nId,6494855 (2026).
17. TVI. O tsunami foi há um ano IOL. %5BURL%5D (2026).
18. TASS. Thailand remembers victims of devastating tsunami https://tass.com/world/769321 (2026).
19. El Mundo. Identificado el Primer Español Muerto en el Maremoto de Asia https://www.elmundo.es/elmundo/2005/02/04/internacional/1107528644.html (2026).
20. Federal Department of Foreign Affairs FDFA. Switzerland to Take Part in the Memorial Ceremony for the Victims of the 2004 Tsunami https://www.admin.ch/gov/en/start/dokumentation/medienmitteilungen.msg-id-55788.html (2026).
21. NTV. Deprem Bölgesinde 6 Türk Kayıp http://arsiv.ntv.com.tr/news/304576.asp (2026).
22. UK Government. Mass Fatalities Incident: Indian Ocean Tsunami 26 December 2004 https://assets.publishing.service.gov.uk/government/uploads/system/uploads/attachment_data/file/86389/MassFatalitiesIncidentIndianOceanTsunami26December2004.pdf (2026).
23. World Bank. Population, Total https://data.worldbank.org/indicator/SP.POP.TOTL (2026).
