# Supplementary material for: Long-term electoral participation among Swedish survivors of the 2004 Indian Ocean tsunami
Source: Nat Commun. 2026 Oct 22;17:11038. doi: 10.1038/s41467-026-78593-8 (PMC13691066; doi:10.1038/s41467-026-78593-8)
Supplement: Supplementary file 2 [file 41467_2026_78593_MOESM2_ESM.pdf]

nature portfolio

Corresponding author(s): Kåre Vernby

Last updated by author(s): Sep 22, 2026

Reporting Summary

Nature Portfolio wishes to improve the reproducibility of the work that we publish. This form provides structure for consistency and transparency in reporting. For further information on Nature Portfolio policies, see our Editorial Policies and the Editorial Policy Checklist.

Statistics

For all statistical analyses, confirm that the following items are present in the figure legend, table legend, main text, or Methods section.

n/a	Confirmed	
☐	☒	The exact sample size (n) for each experimental group/condition, given as a discrete number and unit of measurement
☐	☒	A statement on whether measurements were taken from distinct samples or whether the same sample was measured repeatedly
☐	☒	The statistical test(s) used AND whether they are one- or two-sided<br>*Only common tests should be described solely by name; describe more complex techniques in the Methods section.*
☐	☒	A description of all covariates tested
☐	☒	A description of any assumptions or corrections, such as tests of normality and adjustment for multiple comparisons
☐	☒	A full description of the statistical parameters including central tendency (e.g. means) or other basic estimates (e.g. regression coefficient) AND variation (e.g. standard deviation) or associated estimates of uncertainty (e.g. confidence intervals)
☐	☒	For null hypothesis testing, the test statistic (e.g. F, t, r) with confidence intervals, effect sizes, degrees of freedom and P value noted<br>*Give P values as exact values whenever suitable.*
☒	☐	For Bayesian analysis, information on the choice of priors and Markov chain Monte Carlo settings
☒	☐	For hierarchical and complex designs, identification of the appropriate level for tests and full reporting of outcomes
☒	☐	Estimates of effect sizes (e.g. Cohen's d, Pearson's r), indicating how they were calculated

Our web collection on statistics for biologists contains articles on many of the points above.

Software and code

Policy information about availability of computer code

Data collection	No software was used by the research team for data collection. All register data wereextracted and linked by Statistics Sweden, and the 2023 survey was administered by Statistics Sweden using their web and postal survey systems.
Data analysis	Software: Stata 19 and R 4.5.2. Packages: ebalance (Stata); ggplot2, dplyr, sf, rnaturalearth, rnaturalearthdata (R). Replication Code: Harvard Dataverse, https://doi.org/10.7910/DVN/0AF9U0

For manuscripts utilizing custom algorithms or software that are central to the research but not yet described in published literature, software must be made available to editors and reviewers. We strongly encourage code deposition in a community repository (e.g. GitHub). See the Nature Portfolio guidelines for submitting code & software for further information.

Data

Policy information about availability of data

All manuscripts must include a data availability statement. This statement should provide the following information, where applicable:

- Accession codes, unique identifiers, or web links for publicly available datasets
- A description of any restrictions on data availability
- For clinical datasets or third party data, please ensure that the statement adheres to our policy

The data used in this study originate from Swedish administrative registers and a survey conducted by Statistics Sweden. These data are part of a broader dataset made available to our research group, which allows for the linkage of multiple sources. In accordance with regulatory requirements, the data are stored on an encrypted server, and due to the sensitive nature of the individual-level information, we are contractually prohibited from sharing the data publicly (see Statistics

Sweden's terms of use for microdata access: https://www.scb.se/en/services/ordering-data-and-statistics/microdata/mona--statistics-swedens-platform-for-access-to-microdata/rules-and-regulations/terms-of-use/).

Researchers may apply for access to the same data directly from Statistics Sweden (https://www.scb.se/en/services/ordering-data-and-statistics/microdata/). This requires prior approval from the Swedish Ethical Review Authority (Etikprövningsmyndigheten), and Statistics Sweden grants access for a specified research purpose and project period. At the time of writing, ethical review typically takes about three months and Statistics Sweden's processing of a microdata order about six months, but processing times vary. Requests for guidance on replicating the data linkage can be directed to the corresponding author.

To facilitate replication, we provide our Stata do-files, log files and R scripts, which document all variables used in the study, at Harvard Dataverse (https://doi.org/10.7910/DVN/0AF9U0).

The raw data and sources for Figure 1 are reported in Supplementary Table 14. The analyses reported in Supplementary Table 13 use the National SOM Survey Cumulative Dataset 1986–2022 (version 13), which is publicly available from the SOM Institute, University of Gothenburg, via the Swedish National Data Service (https://snd.se). Source data for all figures are provided with this paper.

Research involving human participants, their data, or biological material

Policy information about studies with human participants or human data. See also policy information about sex, gender (identity/presentation), and sexual orientation and race, ethnicity and racism.

Reporting on sex and gender	Sex is the legal sex recorded in Statistics Sweden's population registers, which is assigned rather than self-reported; the registers contain no information on gender. Sex was considered in the study design as a balancing covariate and as a control variable in all register and survey models. We did not conduct sex-disaggregated analyses because our research questions concern the average effect of exposure on turnout and whether the effect is stronger among those exposed at a younger age; neither question calls for a comparison by sex. Sex-disaggregated counts are provided in the Source Data file.
Reporting on race, ethnicity, or other socially relevant groupings	Our study does not use the constructs of race or ethnicity. Therefore, no justification for their use was necessary, and they were not employed as proxies for other variables such as socioeconomic status or income.
Population characteristics	See the Behavioural & social sciences study design section, Research sample.
Recruitment	In the analysis based on administrative registry data, tsunami survivors were identified through the Swedish National Police Board's registry of survivors, and all identified individuals were included. The comparison group consists of the full Swedish electorate, reweighted with entropy balancing to match the survivors on pre-tsunami characteristics. See Table 4 for sample restrictions used in the article and the Supplementary Information.<br><br>For the survey, conducted in 2023, we used the registry data to survey tsunami survivors and an exactly matched control group. The survey was designed by the authors and administered by Statistics Sweden. Both the questionnaire and the sampling strategy were developed in collaboration with Statistics Sweden, with careful consideration given to the trauma experienced by those exposed. Participants were informed of the survey's subject matter prior to providing informed consent. Participants received no compensation. To ensure eligibility, we excluded individuals who had died or emigrated since the event, as well as those born in 1995 or later, who had been too young when the tsunami occurred; all respondents were therefore adults.<br><br>The goal of the sampling strategy was to obtain two comparable groups (exposed and control) with respect to income, education, region of residence, sex, and age in 2003, prior to the tsunami.<br><br>To preserve the integrity of the sampling process and minimize researcher influence, Statistics Sweden independently selected the final samples for both groups. The registry identified 16,030 tsunami survivors. To ensure eligibility, we excluded individuals who had died or emigrated since the event, as well as those born in 1995 or later. This left 12,534 survivors eligible for matching. Each was paired with 10 randomly selected control individuals with identical sampling characteristics (exact 1:10 matching on age, sex, county of residence, education level and income tertile, all measured in 2003). Eleven survivors without complete matches were excluded, resulting in 12,523 survivors and 125,230 controls. From these, Statistics Sweden randomly selected 5,000 individuals from each group.<br><br>The survey was first distributed digitally on April 4, 2023, followed by a second digital mailing and two postal follow-ups. The final reminder was sent on June 9, 2023. Response rates were 40.3% (2,013 respondents) in the tsunami group and 38.9% (1,943 respondents) in the control group (AAPOR RR2), yielding an overall response rate of 39.6%. Respondents with higher education and income were somewhat overrepresented relative to the sampling frame. To address this self-selection, Statistics Sweden provided calibration weights that align respondents with the frame on register characteristics.
Ethics oversight	This research was reviewed and approved by the Swedish Ethical Review Authority (see approval numbers 2022-02590-01 and 2022-04528-01).

Note that full information on the approval of the study protocol must also be provided in the manuscript.

Field-specific reporting

Please select the one below that is the best fit for your research. If you are not sure, read the appropriate sections before making your selection.

☐ Life sciences ☒ Behavioural & social sciences ☐ Ecological, evolutionary & environmental sciences

For a reference copy of the document with all sections, see nature.com/documents/nr-reporting-summary-flat.pdf

Behavioural & social sciences study design

All studies must disclose on these points even when the disclosure is negative.

Study description

This quantitative observational study investigates the long-term political consequences of exposure to the 2004 Indian Ocean tsunami among Swedish survivors. Using population-wide administrative data, we track individual voter turnout in the 1994, 2010 and 2018 national elections (N up to approximately 7million per election). The main analysis combines entropy balancing on pre-tsunami characteristics, including turnout in 1994, with weighted linear probability models, and finds that exposure did not reduce turnout. Rather turnout rose in both 2010 and 2018 among those exposed to the tsunami. Effects are more than twice as large among survivors who lost a close family member and strongest among those aged 16-25 at the time of the disaster, consistent with habit formation and political socialization as mechanisms of persistence. A follow-up survey in 2023 (3,956 respondents) provides descriptive evidence that survivors still evaluate the government's crisis response negatively and have shifted their party sympathies away from the then-incumbent Social Democrats.

Research sample

The exposed group is the full population of Swedish residents recorded in the National Police Board's tsunami registry (16,030 survivors registered on return), not a sample of it. The comparison group is the remainder of the Swedish electorate, drawn from Statistics Sweden's population-wide registers and reweighted with entropy balancing on pre-tsunami (2003) characteristics. We use the full electorate because it is the relevant counterfactual population for survivors and because its size allows exact reweighting on many covariates. Two analysis samples are used. The restricted samples (main analysis) include only individuals appearing in the 1994 electoral roll: 7,674 exposed and 4,367,005 controls in 2010 (N = 4,374,679) and 7,646 exposed and 3,872,512 controls in 2018 (N = 3,880,158). The unrestricted samples (age-cohort and bereavement analyses) cover the full electorate: N = 6,701,946 in 2010 and N = 7,038,768 in 2018, of whom 241 and 297 exposed individuals are classified as bereaved through linkage to the multigenerational register (318 in the full registry). Before reweighting, survivors differ from the electorate: in the restricted 2010 sample they are 46 percent female (controls 51 percent), have a mean age of about 52 (controls 58), and higher education (36 versus 25 percent with tertiary education) and income; after entropy balancing the control group matches the exposed group exactly on these characteristics. Survivors are thus not representative of the Swedish population, and the study does not claim they are; the estimand is the effect of exposure on the exposed.

For the survey, Statistics Sweden randomly selected 5,000 survivors and 5,000 controls from a sampling frame of 12,523 survivors and 125,230 controls constructed by exact 1:10 matching on 2003 register characteristics; 2,013 and 1,943 responded (3,956 in total; model-specific Ns are smaller owing to item non-response, see Data exclusions). Respondents were on average 59 years old at the time of the survey, 50 percent women, and 52 percent had tertiary education. Calibration weights constructed by Statistics Sweden adjust for differential response.

Sampling strategy

No sampling was performed for the register analyses. All identified survivors and the entire remaining electorate are included, so sample size was determined by the available population data and no a priori power calculation was conducted.

For the survey, each eligible survivor (those who had died or emigrated, and those born in 1995 or later, were excluded) was paired with 10 randomly selected control individuals with identical sampling characteristics (income tertile, education, age, sex and region of residence, measured in 2003). Eleven survivors without complete matches were excluded, resulting in 12,523 survivors and 125,230 controls. From these, Statistics Sweden randomly selected 5,000 individuals from each group. No statistical method was used to predetermine the survey sample size; we surveyed as many individuals as project resources allowed, split equally between the two groups to maximize statistical power for a given total.

Data collection

The administrative data are secondary and were originally collected by Swedish authorities. Turnout in 2010 and 2018 comes from electoral rolls digitized by the Swedish Election Authority and Statistics Sweden; turnout in 1994 comes from electoral rolls digitized by researchers at Uppsala University, covering approximately 90 percent of the electorate. All record linkages were performed by Statistics Sweden, and the research team received pseudonymized data only.

For the survey, Statistics Sweden administered the sampling, distribution and data collection. The survey was sent out four times: twice digitally (answerable via phone, tablet or computer), followed by two postal follow-ups (answered with pen and paper).

Timing

Exposure occurred on 26 December 2004. Pre-treatment covariates are measured in 2003. Outcome data cover the national elections of September 1994 (pre-treatment), September 2010 and September 2018; the register extracts were delivered by Statistics Sweden in January 2023. The survey was fielded between 4 April 2023 (first digital distribution) and 9 June 2023 (final reminder).

Data exclusions

For the register analyses, the main (restricted) samples exclude, by design, individuals who do not appear in the 1994 electoral roll: those not yet eligible to vote before the disaster and the roughly 10 percent of the electorate not covered by the digitized 1994 rolls (this missingness predates the tsunami by a decade and cannot be related to exposure). The unrestricted samples, used for the age-cohort and bereavement analyses, apply no such restriction. Individuals who were not eligible to vote in a given election (e.g. deceased or emigrated) are not part of that election's electorate.

	For the survey, the following groups were excluded from the sampling frame: individuals deemed too young to remember the events (born in 1995 or later), those who had emigrated (as they could not be contacted by Statistics Sweden), those who had died since the tsunami, and 11 survivors for whom no complete set of matched controls could be constructed. Of the 3,956 respondents, estimation samples in the survey regressions are determined by listwise deletion: respondents with missing values on any variable included in a given model (including "Don't know" responses, which are recoded as missing; see Supplementary Note 3) are excluded from that model, so Ns range from 3,196 to 3,621 across analyses, with exact Ns reported in Supplementary Note 3.
Non-participation	The register analyses are based on secondary administrative data, so there was no option for non-participation beyond the exclusions described above; individual consent was not required under Swedish law for this type of register-based research. For the survey, the response rates were 40.3% (2,013 respondents) in the tsunami group and 38.9% (1,943 respondents) in the control group (AAPOR RR2), yielding an overall response rate of 39.6%. Calibration weights provided by Statistics Sweden adjust for non-response.
Randomization	Participants were not randomized. Exposure was determined by presence in the areas affected by the tsunami on 26 December 2004. To adjust for confounding, the full control population is reweighted with entropy balancing so that covariate means (sex, log income, education, age, age squared, county of residence and, in the restricted samples, turnout in 1994; all measured in 2003) exactly match the exposed group, and the balancing covariates are also included in the outcome models. As a further check, we compare bereaved and non-bereaved survivors, who share the characteristics that led them to travel.<br><br>For the survey, the sampling frame was constructed by pairing each eligible survivor with 10 control individuals selected at random within exact-matching strata (age, sex, county of residence, education level and income tertile, measured in 2003), and the survey analyses control for age, sex, education and income.

Reporting for specific materials, systems and methods

We require information from authors about some types of materials, experimental systems and methods used in many studies. Here, indicate whether each material, system or method listed is relevant to your study. If you are not sure if a list item applies to your research, read the appropriate section before selecting a response.

Materials & experimental systems

n/a	Involved in the study
☒	☐ Antibodies
☒	☐ Eukaryotic cell lines
☒	☐ Palaeontology and archaeology
☒	☐ Animals and other organisms
☒	☐ Clinical data
☒	☐ Dual use research of concern
☒	☐ Plants

Methods

n/a	Involved in the study
☒	☐ ChIP-seq
☒	☐ Flow cytometry
☒	☐ MRI-based neuroimaging

Plants

Seed stocks	*Report on the source of all seed stocks or other plant material used. If applicable, state the seed stock centre and catalogue number. If plant specimens were collected from the field, describe the collection location, date and sampling procedures.*
Novel plant genotypes	*Describe the methods by which all novel plant genotypes were produced. This includes those generated by transgenic approaches, gene editing, chemical/radiation-based mutagenesis and hybridization. For transgenic lines, describe the transformation method, the number of independent lines analyzed and the generation upon which experiments were performed. For gene-edited lines, describe the editor used, the endogenous sequence targeted for editing, the targeting guide RNA sequence (if applicable) and how the editor was applied.*
Authentication	*Describe any authentication procedures for each seed stock used or novel genotype generated. Describe any experiments used to assess the effect of a mutation and, where applicable, how potential secondary effects (e.g. second site T-DNA insertions, mosiacism, off-target gene editing) were examined.*
