# Supplementary material for: Long-term electoral participation among Swedish survivors of the 2004 Indian Ocean tsunami
Source: Nat Commun. 2026 Oct 22;17:11038. doi: 10.1038/s41467-026-78593-8 (PMC13691066; doi:10.1038/s41467-026-78593-8)
Supplement: Supplementary file 3 [file 41467_2026_78593_MOESM3_ESM.pdf]

nature portfolio

Peer Review File

Long-term electoral participation among Swedish survivors of the 2004 Indian Ocean tsunami

Corresponding Author: Professor Kåre Vernby

This file contains all reviewer reports in order by version, followed by all author rebuttals in order by version.

Version 0:

Reviewer comments:

Reviewer #1

(Remarks to the Author)
Thank you for the opportunity to review "Long-term political mobilization among survivors after the 2004 Indian Ocean tsunami." This study examines the impact of disasters on voter turnout by analyzing a matched data set that includes voter turnout and covariates. The authors find that those who experienced the tsunami exhibit a significant increase in turnout relative to those who did not. This study draws on a fairly impressive array of data, and improves on existing studies both by disaggregating the negative and positive effects of experiencing disasters, but also by estimating the effect with individual-level data. Additionally, it provides some insights into the possible mechanism underlying these effects. Therefore, I believe that it may be suitable for publication in Nature Communications.

That said, there are a few important issues that I believe need to be addressed before the paper is ready. I have organized my comments thematically below.

1. Mechanism: The paper's main theoretical contribution lies in distinguishing between two mechanisms: retrospective accountability and post-traumatic growth. The former is supported by the data and well-situated in the literature, but the latter is not. That is, it is not clear how or why different kinds of trauma translate specific behaviors. It seems that this is an opportunity to integrate both mechanisms into a coherent model of post-crisis behavior.

2. Causal Identification: Although the matched data set does help in causal identification, it cannot rule out a number of threats to causal identification. Above all, it cannot rule out the possibility of self-selection into the treatment based on unobserved characteristics.

3. External validity: The authors claim that this study can generalize to other settings because it did not involve structural damage to their homes and communities, as in the case of other studies. I do not find this argument convincing. I would encourage the authors to develop argument more and better ground it in the literature.

Reviewer #2

(Remarks to the Author)
NCOMMS-25-67162-T
Long-term political mobilization among survivors after the 2004 Indian Ocean tsunami
This manuscript investigates the long-term political mobilization of Swedish survivors of the 2004 Indian Ocean tsunami. Using a unique registry of survivors linked to population-wide administrative data on voter turnout, the authors argue that disaster exposure led to persistently higher electoral participation in the 2010 and 2018 elections. A follow-up survey in 2023 is used to suggest possible mechanisms, namely dissatisfaction with the government's crisis response and heightened political efficacy. The study is ambitious, leverages an exceptional dataset, and addresses an important question about how disasters shape political behavior. The empirical design is also creative in that it focuses on survivors who returned to intact institutions, thereby avoiding the confounding effects of infrastructural destruction.

Despite these strengths, there are theoretical and empirical shortcomings that, in my view, prevent the paper from being publishable in its present form. The first and most important concern is conceptual. The authors present the study as isolating “motivational” effects, since survivors returned to functioning institutions and therefore faced no additional resource constraints on participation. I find this inference much too strong. Disasters can certainly increase motivation to engage politically, but trauma also has indirect consequences that the study cannot capture such as long-term mental health impacts, family disruption, or subtle economic strain. These channels matter, and they do not fit neatly into either “motivation” or “resources.” To treat all residual differences as motivation oversimplifies the reality and, to my mind, overstates the theoretical leverage. A more careful and honest framing would be to say that the results are consistent with motivational change, while acknowledging that other unobserved processes could be at work.
A second issue is the reliance on the 1994 election as a baseline. These pre-disaster data come from digitized voting rolls with incomplete coverage, while the post-disaster elections rely on complete registers. That discontinuity introduces a measurement problem that undermines the placebo tests. If the 1994 data underrepresent certain groups, and it seems quite likely that they do, then faint pre-existing differences may be hidden. Yet the manuscript treats 1994 as if it were a firm benchmark. This is overstated. The possibility that measurement error clouds the baseline should be taken more seriously and discussed directly, since it bears on whether we can really be confident about the pre-treatment equivalence of survivors and controls.
I also have concerns about the matching strategy. A great deal of the analysis rests on exact matching within broad income tertiles. In my view, this is simply too crude. Because the income–turnout relationship is nonlinear, tertile matching risks leaving behind important imbalances that matter for political participation. The supplementary analyses with income deciles move in the right direction, but they are underdeveloped and presented as secondary, almost an afterthought. What is more, the fact that estimates shift somewhat across specifications suggests that the results are not as robust as claimed. Stronger balancing approaches such as entropy balancing, coarsened exact matching, or related methods would provide greater reassurance. At the very least, the paper needs to elevate these robustness checks into the main analysis rather than treating them as supplementary reassurance.
Selection into exposure is another area where I am not persuaded by the current treatment. The authors note that survivors and controls had nearly identical turnout in 1994, and they take this as evidence that unobserved predispositions are not driving the results. But turnout is a very coarse proxy for political engagement. Swedes who vacationed in Thailand in 2004 may well have been systematically different from those who did not, that is, more cosmopolitan, more trusting of institutions, or more politically curious. These are not traits that administrative records can measure, yet they plausibly condition how people respond to traumatic events. The manuscript acknowledges this possibility only briefly, but it deserves more serious attention. Without it, the claim that selection can be safely ignored is not convincing.
The evidence for mechanisms is, to my mind, the weakest part of the paper. The 2023 follow-up survey is used to suggest retrospective blame of the Social Democrats and post-traumatic growth in political efficacy. But two decades after the event, I cannot accept this as causal evidence. Survivors’ recollections of dissatisfaction with the government’s response are almost certainly colored by subsequent political developments, and the observed partisan shifts could just as easily reflect broader realignments in Swedish politics. The efficacy measure is also thin, covering only a narrow slice of what the literature usually means by post-traumatic growth. And with a 40 percent response rate, selective non-response is a real concern. These problems are acknowledged in passing, but not given the weight they deserve. At best, the survey can add descriptive texture; it cannot credibly serve as evidence for mechanisms.
Finally, I think the authors need to be much more cautious about the substantive size of the effects. The estimated turnout increases of around one to two percentage points are statistically robust and, yes, interesting given Sweden’s high baseline turnout, but they are modest in absolute terms. Presenting them as a 15–20 percent reduction in abstention exaggerates their importance. The real contribution here is the persistence of a small effect, not the transformation of the electoral landscape. Readers would be better served by a restrained presentation that accurately conveys the modest scale of the change.

Reviewer #3

(Remarks to the Author)
“Long-term political mobilization among survivors after the 2004 Indian Ocean tsunami”
Nature Communications MS ID: NCOMMS-25-67162-T

This study investigates an innovative question: can individuals be politically mobilized by natural disasters that they have experienced abroad? The authors analyze the behavior of Swedish survivors of the 2004 Indian Ocean tsunami. Leveraging this case, they disentangle political engagement effects from voting costs induced by infrastructure damage or displacement. The empirics compare Swedish returnees exposed to the disaster with a control group matched on basic demographics across the 1994, 2010, and 2018 elections using informationally rich, register-based individual-level data. The results suggest that individuals personally affected by the tsunami became more politically active upon returning home. A complementary survey identifies enduring dissatisfaction with the incumbent government at the time of the disaster as a key mechanism. Overall, the findings offer insights on voter responses to natural disasters and speak to public debates about civic engagement in the aftermath of large-scale shocks.

This is a carefully executed study that features an innovative design and rich data. As such, it has the potential to make valuable contributions to the literature on the politics of natural disasters. I recommend a major revise and resubmit.

Major Comments:
1. The article’s structure and framing require substantial revisions. Currently, the authors devote excessive space to

supplementary analyses of the main effect of tsunami exposure on voter turnout, specifically in the subsections on treatment intensity, generalizability, and selection into travel. These sections should be considerably condensed and relegated to the appendix. The key threat to identification, the potential violation of the parallel trends assumption due to selection into travel, should be addressed earlier and more systematically, rather than dispersed across multiple robustness checks. Conversely, the mechanism linking tsunami exposure to the experience of failed preparedness investments warrants greater attention. In particular, the authors identify a persistent negative effect of exposure on voting for the then-incumbent Social Democratic Party, yet this finding requires a fuller theoretical and empirical explanation (see below). Overall, the balance of the results section demands major revision.
2. The theoretical argument to leverage this case to isolate the accountability channel is compelling and innovative. However, it remains unclear why the accountability channel that the authors propose as underpinning the effects on turnout should persist over multiple electoral cycles, despite government turnover. The argument implies that voters continue to hold the government accountable for improving disaster preparedness in the distant future although incumbent politicians and parties have long changed. Thus, given that the authors analyze long-term effects on the 2018 election, they should clarify why persistent turnout should still be understood as an expression of accountability demands when a different party (or different personnel) is in office or when these factors have changed multiple times.
3. The persistent negative effect on Social Democratic Party support in 2022, eighteen years after the disaster, challenges the authors' argument that accountability demands motivate voters. Instead, it points to an enduring shift in party preferences (realignment?) consistent with a socialization effect, particularly given the stronger impact observed among younger voters (along the lines of the impressionable years argument, Brim/Kagan 1980; Franklin 2004). The authors could address this, e.g., by presenting evidence that affected voters continue to be driven by substantive concerns, such as demands for improved preparedness investments, that emerged in the aftermath of the disaster and persist over time. This would be all the more surprising given the absence of a correlation between disaster exposure and mass support for preparedness investment (Bechtel and Mannino 2020). If such an analysis is not possible due to data limitations, the authors should at minimum provide a more detailed discussion of the enduring effects on party preferences and clarify how these long-term dynamics relate to their core argument.
4. Building on this, the authors should also provide a clearer interpretation of both (i) the persistence of turnout effects (see Theory) and (ii) the increase in the turnout gradient between 2010 and 2018. We know from previous studies that electoral returns to successful disaster relief decay over time. Why and how can accountability failures create growing effects several electoral cycles after the event?
5. In Table 1, the pre-treatment election analysis in Model (1) mechanically excludes individuals who were not yet eligible to vote in 1994, but Models (2) and (3) appear to include this subgroup. This table should instead display the results with a consistent sample, where the post-treatment models also exclude individuals who were not yet eligible to vote, as is reported in Tables S.4/5. Generally, the authors should be significantly more transparent about the samples used in the analyses, which can be easily accommodated in Table footnotes.
6. Heterogeneous age effects: The results in Table S.4 and S.5 indicate that the effects of tsunami exposure are substantially larger among younger voters and those who were not yet eligible to vote in 1994. Especially in the absence of additional pre-treatment election years, which is a data limitation beyond the authors' control, this introduces additional concerns about parallel trends. Specifically, demographics for young adults, which appear to be partially assigned based on parents' demographics, may work less well in capturing their life outcomes. Heterogeneous trends among young voters where demographics are less predictive of future life outcomes thus remains a concern that should at least be verbally addressed, although the main effects hold in the restricted sample.

Minor Comments

1. The authors show that the effects are concentrated among low-income voters and previous non-voters. Both results could be driven by the age dynamic, as young (adult) voters are more likely to earn lower incomes. The interaction with non-voting in 1994 also requires additional explanation. Given that 1994 turnout is the only pre-treatment condition in the main analysis, this analysis seems essentially equivalent to the main effects, where the positive effects on turnout should arise in significant parts from voters who abstained in 1994 and turned out in 2010/2018–at least if they are not purely driven by changing behavior in the control group.
2. The authors could elaborate more on the concept of post-traumatic growth and its relation to turnout. The link remains difficult to grasp in the analysis.
3. A descriptive table of the composition of bereaved individuals could be added in the Appendix for an improved substantive interpretation of effects in Figure 3. Specifically, were bereaved individuals disproportionately children/parents at the time?
4. The analyses conducted in the "Generalizability" section do not necessarily address the generalizability of the results but rather perform extensions.

Reviewer #4

(Remarks to the Author)
I co-reviewed this manuscript with one of the reviewers who provided the listed reports. This is part of the Nature Communications initiative to facilitate training in peer review and to provide appropriate recognition for Early Career Researchers who co-review manuscripts.

Version 1:

Reviewer comments:

Reviewer #1

(Remarks to the Author)
The authors have done much to strengthen the manuscript. I remain somewhat skeptical about the validity of the research design due to multiple threats to causal inference, and the proposed mechanism remains somewhat underdeveloped, which in turns means that the overall contribution to theory is modest. That said, I believe the manuscript merits publication given its innovative use of a unique event analysis to evaluate the effect of natural disasters on political participation.

Reviewer #2

(Remarks to the Author)
NCOMMS-25-67162A
“Long-term political mobilization among survivors after the 2004 Indian Ocean tsunami”

Thank you for the opportunity to review the revised manuscript. The paper has improved substantially. The authors have strengthened the empirical strategy, especially by replacing the original matching approach with entropy balancing and by adding complementary analyses that more directly address selection into exposure. The theoretical framing is also clearer, and the decision to treat the 2023 survey evidence as descriptive rather than causal is appropriate.

My remaining concern is about how the authors characterize what the design identifies. The manuscript still suggests that the setting allows them to isolate a “motivational channel,” because survivors returned to intact institutions and did not face the resource constraints common in many disaster settings. This claim appears already in the abstract, and I think it remains somewhat too strong. Disaster exposure can generate psychological, social, and economic consequences that are not directly observed in the data and that do not map neatly onto a motivation-versus-resources distinction. These pathways cannot be cleanly disentangled in the present design.

I therefore encourage the authors to further temper this language and to frame the results as consistent with motivational responses, rather than as isolating a specific motivational channel. This is a matter of interpretation rather than a fundamental problem with the analysis. Consequently, I recommend publication subject to minor revisions.

Reviewer #3

(Remarks to the Author)
“Long-term political mobilization among survivors after the 2004 Indian Ocean tsunami”
Nature Communications MS ID: NCOMMS-25-67162-A

The authors successfully responded to the comments and the paper makes a valuable contribution to the literature on the political consequences of natural disasters. I recommend acceptance conditional on several minor points. The revisions in response to these comments can in my view be evaluated in-house.
1. The additional difference-in-differences analysis discussed on p. 9-10 seems redundant given the inclusion of turnout in 1994 as a control variable. The authors write that the results presented in S.11 are comparable, and although the estimates are virtually identical the standard errors are slightly larger. This can be solved by dropping the additional analysis from the main body. In contrast, the additional sensitivity check bolsters the credibility of the estimated effects.
2. Figure 3, Panels A-B: The authors write that “exposed respondents report receiving almost no support from public authorities in the aftermath of the disaster” and that “[t]hose who did receive assistance overwhelmingly judged it inadequate”. I wonder whether this is actually consistent with the results in Panel A which indicate that the average rating of support from public authorities among both exposed and severely exposed respondents is at around 2, which is the midpoint of the scale (which ranges from 0 to 4).
3. All Figures: please ensure informative axis titles. Examples include: Figure 2 is missing information about the unit in which the estimated effects are measured. The notes say they are “The Effect of Tsunami Exposure on Voter Turnout”, so presumably these are changes in percentage points. This should be added as axis title information. Another example is Panel F, Figure 3 which information about the party codes is missing. Ideally, the party names are spelled out in the plot or at least informative labels should be used and explained in the note.
4. Repetitive language: The authors conclude multiple sections with the phrase „Taken together”, which makes the prose unnecessarily repetitive. Consider varying these transitions.

Reviewer #4

(Remarks to the Author)
I co-reviewed this manuscript with one of the reviewers who provided the listed reports. This is part of the Nature Communications initiative to facilitate training in peer review and to provide appropriate recognition for Early Career Researchers who co-review manuscripts.

Open Access This Peer Review File is licensed under a Creative Commons Attribution 4.0 International License, which permits use, sharing, adaptation, distribution and reproduction in any medium or format, as long as you give appropriate credit to the original author(s) and the source, provide a link to the Creative Commons license, and indicate if changes were made.
In cases where reviewers are anonymous, credit should be given to 'Anonymous Referee' and the source.
The images or other third party material in this Peer Review File are included in the article's Creative Commons license, unless indicated otherwise in a credit line to the material. If material is not included in the article's Creative Commons license and your intended use is not permitted by statutory regulation or exceeds the permitted use, you will need to obtain permission directly from the copyright holder.
To view a copy of this license, visit https://creativecommons.org/licenses/by/4.0/

Response to Reviewers

Manuscript: *Long-term political mobilization among survivors after the 2004 Indian Ocean tsunami*
Journal: *Nature Communications*

Thank you for your thorough evaluation of our manuscript and for the opportunity to submit a revised version to *Nature Communications*. We are grateful for the detailed and constructive feedback from the reviewers.

The revisions are extensive. We replaced the original dual-mechanism framework with a single account rooted in accountability demands, habit formation, and political socialization during the impressionable years; replaced exact matching with entropy balancing as the main analytical strategy; and reorganized the Results section into three streamlined sections, each addressing a clearly defined question. The Main Findings section, rewritten in its entirety, is now devoted exclusively to bounding the risk of selection into treatment. A new section on age-cohort effects tests the implication that the mobilization should be strongest among those exposed during their impressionable years. The survey section has been reframed as descriptive evidence of a lasting partisan realignment rather than as a causal mechanism test. We have also been more careful throughout in laying out the scope conditions of both our theoretical argument and our findings. We believe these changes have substantially strengthened the contribution and the clarity of the manuscript.

Reviewer Remarks and Responses

Below we reproduce each comment verbatim in a shaded box and provide our response immediately below each comment.

Reviewer #1 (Remarks to the Author)

Thank you for the opportunity to review "Long-term political mobilization among survivors after the 2004 Indian Ocean tsunami." This study examines the impact of disasters on voter turnout by analyzing a matched data set that includes voter turnout and covariates. The authors find that those who experienced the tsunami exhibit a significant increase in turnout relative to those who did not. This study draws on a fairly impressive array of data, and improves on existing studies both by disaggregating the negative and positive effects of experiencing disasters, but also by estimating the effect with individual-level data. Additionally, it provides some insights into the possible mechanism underlying these effects. Therefore, I believe that it may be suitable for publication in Nature Communications. That said, there are a few important issues that I believe need to be addressed before the paper is ready. I have organized my comments thematically below.

1. Mechanism: The paper's main theoretical contribution lies in distinguishing between two mechanisms: retrospective accountability and post-traumatic growth. The former is supported by the data and well-situated in the literature, but the latter is not. That is, it is not clear how or why different kinds of trauma translate specific behaviors. It seems that this is an opportunity to integrate both mechanisms into a coherent model of post-crisis behavior.

Response: We thank the reviewer for pressing us on the theoretical framing. In the revised manuscript, we have substantially reworked the theoretical contribution. Rather than treating

retrospective accountability and post-traumatic growth as two parallel mechanisms, we now develop a single, empirically tractable account rooted in established political science research: disaster exposure triggers an initial accountability response that mobilizes survivors, and this initial mobilization is then consolidated into a durable behavioral pattern through habit formation and political socialization, particularly among those exposed during their impressionable years [Gerber et al., 2003, Coppock and Green, 2016, Krosnick and Alwin, 1989, Franklin, 2004, Sears and Brown, 2013].

This reframing has two advantages over the original. First, it builds on the well-established findings in political behavior that voting is habit-forming and that experiences during early adulthood leave an especially durable imprint, rather than on a psychological construct (post-traumatic growth) that is difficult to test with our data. Second, each link in the chain can be evaluated empirically: the main analysis, rewritten in its entirety, establishes the long-term effect of exposure on turnout (described in detail below); a new section on age-cohort effects shows that the effect was strongest among those in their impressionable years (aged 16–25) at the time of the disaster. The descriptive survey evidence, now presented explicitly as such rather than as a mechanism test (see our response to R2 below), suggests that the accountability judgment crystallized into lasting changes in party sympathies.

We have not abandoned post-traumatic growth entirely, but we have repositioned it. Rather than presenting it as a one of the mechanisms, we now note in the Discussion that our finding of mobilization rather than demobilization is consistent with a broader literature suggesting that trauma need not lead exclusively to withdrawal and disengagement, and that post-traumatic growth remains an interesting avenue for future research with richer psychological data than ours.

These changes are reflected in the revised introduction (paragraphs 2 and 7–9), the revised sections containing main findings and age-cohort effects, and in the discussion.

> *2. Causal Identification: Although the matched data set does help in causal identification, it cannot rule out a number of threats to causal identification. Above all, it cannot rule out the possibility of self-selection into the treatment based on unobserved characteristics.*

Response: We agree that selection into treatment, i.e. the possibility that Swedes who vacationed in Thailand in December 2004 differ from those who did not on unobservable dimensions that also predict turnout trends, is the central identification concern in our study. We have substantially revised the manuscript to address this concern, both analytically and in how we frame our findings.

Analytical changes. The revised Main Findings section is now organized entirely around the selection threat, proceeding through a sequence of increasingly demanding strategies designed to bound its severity.

First, and on the suggestion of R2, we replace the original exact-matching procedure with **entropy balancing** [Hainmueller, 2012], which reweights the control group so that its covariate moments match those of the treated group continuously rather than within discrete bins. Unlike matching on income tertiles or deciles, entropy balancing achieves balance on the log of income and—crucially—on **pre-tsunami turnout in 1994**, which serves as a summary measure of the latent political engagement that observable demographics alone may not capture. We conduct this analysis exclusively on the **restricted sample** of individuals who appear in the 1994 turnout file, ensuring that neither aging into eligibility nor post-tsunami migration can alter the composition of the treatment or control groups. With this demanding design, we estimate a tsunami-exposure effect of approximately 1.4 percentage points in both 2010 and 2018—closely aligned with our earlier estimates.

Second, because entropy balancing renders observed covariates uncorrelated with treatment by

construction, formal sensitivity frameworks such as Cinelli and Hazlett [2020] cannot be applied in their standard form (the benchmarking machinery requires observable variation in treatment assignment as a reference point, which no longer exists after reweighting). We therefore report an **informal sensitivity exercise** using the standard omitted variable bias formula. We ask how strongly an unobserved confounder would need to predict both treatment assignment and the outcome to explain away the estimated effect. Anchoring the comparison in education—a canonical predictor of turnout that is associated with a 3.8 percentage-point increase in the entropy-balanced sample—an unobserved confounder of the same magnitude would need to raise the probability of tsunami exposure by 37 percentage points to eliminate the effect entirely. A confounder of this strength, operating net of a covariate distribution that has already been equalized, is difficult to sustain empirically.

Third, we estimate **entropy-balanced difference-in-differences models** that difference out all time-invariant individual-level characteristics—including value orientations such as cosmopolitanism or institutional trust that administrative data cannot measure. The results are of comparable magnitude to the cross-sectional entropy-balanced estimates.

Fourth, we present a **bereavement analysis** that approaches the selection concern from a design-based angle. Traits associated with selecting into overseas travel are shared by all tsunami-exposed individuals; by restricting the comparison to survivors, we effectively condition on propensity to travel. Within this travel-matched population, we leverage bereavement—the loss of an immediate family member—to construct a dose–response test. The turnout effect among bereaved survivors is approximately 3 percentage points larger than among non-bereaved survivors (p=0.041p in 2010; p=0.023 in 2018), consistent with a causal interpretation and difficult to reconcile with a pure selection into travel account.

Language and framing. We have also revised the manuscript to be more explicit about the observational nature of the study and the limits of our identification strategy. In the introduction, we now state that "because the study is observational, the central identification concern is that Swedes who vacationed in Southeast Asia may differ from those who did not on dimensions that also predict turnout trends" (paragraph 6). The Main Findings section opens by naming the selection threat directly and frames all subsequent analyses as strategies to bound the risk of omitted variable bias. At the close of the identification sequence, we note explicitly that "as with any observational study, selection on unobservables cannot be definitively ruled out, but the convergence of multiple independent strategies, each addressing the threat from a different angle, makes a pure selection account difficult to sustain." We are thus careful throughout to present the evidence as collectively making a selection account implausible rather than claiming it has been eliminated.

3. External validity: The authors claim that this study can generalize to other settings because it did not involve structural damage to their homes and communities, as in the case of other studies. I do not find this argument convincing. I would encourage the authors to develop argument more and better ground it in the literature.

Response: We appreciate the reviewer raising this point, as it highlights an ambiguity in our original framing that we have now corrected. We did not intend to argue that the Swedish case increases external validity. Our argument is about internal validity: because survivors returned home to intact communities, we can isolate the effect of the disaster experience itself from the displacement, infrastructure destruction, and resource loss that raise the costs of voting in most disaster settings. We think this is an important design advantage for identifying the motivational channel, but we do not want to claim that the estimated effects transfer directly to other contexts.

In the revised manuscript, we have taken care to separate the two issues. In the introduction, we now write that the setting allows us to attribute any change in voting behavior "to the immediate experience of the disaster itself rather than to the displacement, infrastructure destruction, and resource loss that raise the costs of voting in most disaster settings." In the Discussion, we are explicit about the limits of generalizability: because most disasters do raise the costs of voting through destruction and displacement, our estimates capture a component of the overall effect that is typically confounded with these cost increases. We do not claim that the magnitude of our estimates would transfer directly to other settings. However, we note that the motivational response we document may be latent in other disaster-affected populations, and that measures designed to ease the costs of voting, such as flexible registration, expanded early voting, or temporary polling stations, could counteract the drop in mobilization in the wake of natural disasters that is frequently observed.

Reviewer #2 (Remarks to the Author)

> *This manuscript investigates the long-term political mobilization of Swedish survivors of the 2004 Indian Ocean tsunami. Using a unique registry of survivors linked to population-wide administrative data on voter turnout, the authors argue that disaster exposure led to persistently higher electoral participation in the 2010 and 2018 elections. A follow-up survey in 2023 is used to suggest possible mechanisms, namely dissatisfaction with the government's crisis response and heightened political efficacy. The study is ambitious, leverages an exceptional dataset, and addresses an important question about how disasters shape political behavior. The empirical design is also creative in that it focuses on survivors who returned to intact institutions, thereby avoiding the confounding effects of infrastructural destruction.*

> *1. Despite these strengths, there are theoretical and empirical shortcomings that, in my view, prevent the paper from being publishable in its present form. The first and most important concern is conceptual. The authors present the study as isolating "motivational" effects, since survivors returned to functioning institutions and therefore faced no additional resource constraints on participation. I find this inference much too strong. Disasters can certainly increase motivation to engage politically, but trauma also has indirect consequences that the study cannot capture such as long-term mental health impacts, family disruption, or subtle economic strain. These channels matter, and they do not fit neatly into either "motivation" or "resources." To treat all residual differences as motivation oversimplifies the reality and, to my mind, overstates the theoretical leverage. A more careful and honest framing would be to say that the results are consistent with motivational change, while acknowledging that other unobserved processes could be at work.*

Response: We agree with the reviewer that our use of "motivation" was insufficiently precise in the original manuscript and risked overstating the theoretical leverage of the design. We have addressed this concern through both language changes and additional discussion.

First, we have revised the framing and language in the introduction. We clarify that by "motivational channel" we mean the effect of the disaster experience itself, net of the displacement, resource loss, and infrastructure destruction that raise the costs of voting in most disaster settings. This contrast is now made explicit in several place. In the abstract, we write: "Because the displacement, resource loss, and infrastructure destruction that raise the costs of voting in most

disaster settings are absent in our case, we can isolate the motivational channel." In the introduction, we state that our case gives us the "opportunity to disentangle the motivational effects of disaster exposure on political behavior from the resource constraints and cost-of-voting increases that confound most existing studies." and that change in voting behavior "can be attributed to the experience of the disaster itself rather than to the displacement, infrastructure destruction, and resource loss that raise the costs of voting in most disaster settings."

Second, in the discussion, we now explicitly acknowledge the reviewer's point that trauma can have indirect consequences—such as long-term mental health impacts, family disruption, or subtle economic strain—that do not fit neatly into either a "motivation" or a "resources" category. We note, however, that the positive direction of the effect weighs against explanations rooted in the demobilizing consequences of trauma: if mental health deterioration or family disruption were the dominant pathway, we would expect turnout to fall, not rise.

2. A second issue is the reliance on the 1994 election as a baseline. These pre-disaster data come from digitized voting rolls with incomplete coverage, while the post-disaster elections rely on complete registers. That discontinuity introduces a measurement problem that undermines the placebo tests. If the 1994 data underrepresent certain groups, and it seems quite likely that they do, then faint pre-existing differences may be hidden. Yet the manuscript treats 1994 as if it were a firm benchmark. This is overstated. The possibility that measurement error clouds the baseline should be taken more seriously and discussed directly, since it bears on whether we can really be confident about the pre-treatment equivalence of survivors and controls.

Response: We thank the reviewer for raising this issue. The 1994 turnout data were digitized by researchers at Uppsala University and cover approximately 90 percent of the Swedish electorate [Lindgren et al., 2019]. We agree that this incomplete coverage deserves direct discussion, and we have added a detailed treatment of the 1994 data in the Methods section of the revised manuscript (see the Registry Data subsection).

That said, we believe the concern is substantially mitigated by our research design, for three reasons.

First, and most importantly, the missingness in the 1994 file is not systematically related to tsunami exposure, which occurred a decade later. What matters for our design is not perfect population coverage, but comparability between the exposed individuals and their matched controls *within* the subset observed in 1994. Both groups are drawn from the same underlying 1994 dataset, so any residual incompleteness affects survivors and controls symmetrically. This is confirmed empirically: the pre-tsunami turnout rates in Table 1 are nearly identical across groups both before and after entropy balancing (89.2% vs. 89.2%), with post-balancing differences indistinguishable from zero.

Second, our revised main analysis substantially strengthens the role of the 1994 data relative to the original submission. Rather than using 1994 solely as a placebo test, we now *condition on 1994 turnout* directly via entropy balancing. The restricted sample includes only individuals who appear in the 1994 turnout file, and their pre-tsunami voting behavior is among the covariates on which the control group is reweighted. This means that any measurement error in the 1994 data that is common to treated and control units is absorbed by the balancing procedure, and any differential measurement error would need to be correlated with tsunami exposure—an event that had not yet occurred—to bias the results.

Third, we complement the entropy-balanced cross-sectional estimates with entropy-balanced difference-in-differences models (reported in the Supplementary Information). The DiD specification

differences out all time-invariant individual-level characteristics, including any stable traits that the 1994 data might imperfectly capture. The fact that the DiD estimates are of comparable magnitude to the cross-sectional entropy-balanced estimates provides reassurance that measurement limitations in the 1994 baseline are not driving our findings.

We have revised the manuscript to be transparent about the coverage limitations of the 1994 data rather than treating them as a firm benchmark. The relevant passage in the Methods section now reads: "The pre-tsunami turnout measure comes from digitized electoral rolls collected by researchers at Uppsala University, covering approximately 90 percent of the Swedish electorate in 1994. Although these data were digitized outside of Statistics Sweden, they provide near-complete coverage and have been validated in previous research as a highly accurate source for individual turnout. Importantly, missingness in the 1994 file is not systematically related to tsunami exposure, which occurred a decade later. What matters for our design is therefore not perfect population coverage, but comparability between the exposed individuals and their matched controls within the subset observed in 1994."

3. I also have concerns about the matching strategy. A great deal of the analysis rests on exact matching within broad income tertiles. In my view, this is simply too crude. Because the income–turnout relationship is nonlinear, tertile matching risks leaving behind important imbalances that matter for political participation. The supplementary analyses with income deciles move in the right direction, but they are underdeveloped and presented as secondary, almost an afterthought. What is more, the fact that estimates shift somewhat across specifications suggests that the results are not as robust as claimed. Stronger balancing approaches such as entropy balancing, coarsened exact matching, or related methods would provide greater reassurance. At the very least, the paper needs to elevate these robustness checks into the main analysis rather than treating them as supplementary reassurance.

Response: We thank the reviewer for pointing us in the direction of using matching techniques that allow for a more fine-grained matching. As we describe above, and in our response to R1, we have replaced the original exact-matching procedure with entropy balancing [Hainmueller, 2012], which achieves continuous balance on the log of income rather than within discrete bins, as the main analytical strategy throughout the paper. In our response to R1, Comment 2, we describe the full set of analytical changes, including the entropy-balanced restricted-sample design, the difference-in-differences specification, and the bereavement dose–response test, all of which use entropy balancing. We also use entropy balancing in our new analyses of age cohort effects, as well as in our revised analyses of whether exposure affected core or peripheral voters most.

As for the reviewer's point about estimates shifting somewhat across specifications in the previous version of manuscript, we note that in the new main analysis using entropy-balancing, the estimates are nearly identical for the 2010 and 2018 elections (Table 2). These results are also almost indistinguishable from those in the most comparable analysis (in terms of the sample employed): the entropy-balanced difference-in-differences models (see Supplementary Information, **Table S.11**).

4. Selection into exposure is another area where I am not persuaded by the current treatment. The authors note that survivors and controls had nearly identical turnout in 1994, and they take this as evidence that unobserved predispositions are not driving the results. But turnout is a very coarse proxy for political engagement. Swedes who vacationed in Thailand in 2004 may well have been systematically different from those who did not, that is, more cosmopolitan, more trusting of institutions, or more politically curious. These are not traits that administrative records can measure, yet they plausibly condition how people respond to traumatic events. The manuscript acknowledges this possibility only briefly, but it deserves more serious attention. Without it, the claim that selection can be safely ignored is not convincing.

Response: We agree that cosmopolitanism, institutional trust, and related traits represent important versions of the selection concern. As we describe in detail in our response to R1, Comment 2, we have substantially revised the identification strategy to address this threat, replacing exact matching with entropy balancing on pre-tsunami turnout and a rich set of covariates, and complementing it with a sensitivity analysis, difference-in-differences models, and a within-treatment bereavement dose-response test. We do not repeat those details here but note that each strategy was designed with precisely the reviewer's concern in mind.

Beyond those analytical changes, we want to address the specific traits the reviewer raises. Two pieces of evidence speak directly to the cosmopolitanism hypothesis. First, survey data on the Swedish population show that traveling abroad is not significantly associated with the likelihood of voting in national elections (Supplementary Information, **Table SI.15**), providing direct evidence that the travel-turnout link assumed by the cosmopolitanism concern does not hold empirically. Second, and more importantly, the bereavement analysis addresses a subtler version of the concern: even if cosmopolitanism does not predict baseline turnout but instead predicts responsiveness to politically salient events, this cannot explain why bereaved survivors — who share the same propensity to travel as non-bereaved survivors — show substantially larger effects.

We have also revised the manuscript to foreground these concerns more prominently. The Main Findings section now opens by naming selection into treatment as the central identification challenge, and the introduction states explicitly that "because the study is observational, the central identification concern is that Swedes who vacationed in Southeast Asia may differ from those who did not on dimensions that also predict turnout trends." We no longer treat selection as a concern that can be safely set aside; instead, we present the cumulative evidence as making a selection account implausible while acknowledging that it cannot be definitively ruled out.

5. The evidence for mechanisms is, to my mind, the weakest part of the paper. The 2023 follow-up survey is used to suggest retrospective blame of the Social Democrats and post-traumatic growth in political efficacy. But two decades after the event, I cannot accept this as causal evidence. Survivors' recollections of dissatisfaction with the government's response are almost certainly colored by subsequent political developments, and the observed partisan shifts could just as easily reflect broader realignments in Swedish politics. The efficacy measure is also thin, covering only a narrow slice of what the literature usually means by post-traumatic growth. And with a 40 percent response rate, selective non-response is a real concern. These problems are acknowledged in passing, but not given the weight they deserve. At best, the survey can add descriptive texture; it cannot credibly serve as evidence for mechanisms.

Response: We wish to begin by acknowledging that this comment substantially improved the

paper. As we describe in our response to R1, Comment 1, the reviewer's critique, together with R1's parallel concern about the theoretical architecture, prompted us to replace the original dual-mechanism framework (retrospective accountability and post-traumatic growth) with a single, empirically tractable account rooted in accountability demands, habit formation, and political socialization. Post-traumatic growth is no longer presented as aone of the mechanisms but is noted in the Discussion as a broader literature consistent with the positive direction of our findings.

The survey section has been rewritten accordingly. It is now titled "Did the Government's Crisis Response Trigger a Lasting Realignment?" and is presented explicitly as descriptive evidence, not as a causal mechanism test. We state at the outset that because these data are cross-sectional and collected nearly two decades after the event, the patterns reported below are descriptive; they cannot by themselves establish causal mechanisms but can indicate whether the attitudinal and partisan correlates of exposure are consistent with a lasting accountability response." This caveat is repeated in the section's concluding paragraph. The section now walks through three elements of the survey evidence in sequence: (1) survivors' reports of receiving almost no support from public authorities (Figure 5, Panels A–B); (2) the persistence and specificity of negative evaluations of the government's crisis response nearly twenty years later (Panels C–E; full regression estimates in Supplementary Information Table SI.18); and (3) the partisan consequence, with effects concentrated on the Social Democrats and Moderates (Panel F; full regression estimates in Supplementary Information Table SI.20).

While we are explicit that the survey provides only descriptive evidence, we also want to address the reviewer's specific concerns about the survey data. On the response rate: at 40 percent (calculated following AAPOR RR2), our rate is somewhat lower than Sweden's largest and most well-resourced public opinion survey, the national SOM survey fielded annually by the SOM Institute at the University of Gothenburg, whose comparable rate was 47 percent in 2022 [Bergquist, 2023]. The SOM survey's higher rate reflects substantially greater fieldwork resources, including five postal mailings, four SMS reminders, and a 109-day field period, compared to our two digital mailings and two postal follow-ups over 67 days. Crucially, however, the response rates in our two groups are nearly identical (40.3% for the tsunami group and 38.9% for controls), which makes differential nonresponse an unlikely driver of the observed patterns. To reduce nonresponse bias, Statistics Sweden provided calibration weights that minimize the difference between the weighted respondents and the population in the same stratum (the tsunami population on the one hand, and the covariate-matched universe of controls on the other) with respect to relevant registry data; we report weighted and unweighted descriptive statistics (Table 5).

On the concern that partisan shifts may reflect broader realignments in Swedish politics rather than the tsunami experience we agree that one needs to be careful. However, the survey analyses are done on samples that are matched on age, sex, education, income tertiles and geographical region (and also include controls for the matching variables), and the effects are concentrated on the Social Democrats (negative) and the Moderates (positive), the two parties most directly implicated in the accountability narrative. While these evidence are descriptive, we note that no other party shows shifts of comparable magnitude (Supplementary Information Table SI.20).

6. Finally, I think the authors need to be much more cautious about the substantive size of the effects. The estimated turnout increases of around one to two percentage points are statistically robust and, yes, interesting given Sweden's high baseline turnout, but they are modest in absolute terms. Presenting them as a 15–20 percent reduction in abstention exaggerates their importance. The real contribution here is the persistence of a small effect, not the transformation of the electoral landscape. Readers would be better served by a restrained presentation that accurately conveys the modest scale of the change.

Response: We accept this criticism and have made two changes to the presentation.

First, we have demoted the relative framing. The reviewer is right that leading with a "15–20 percent reduction in abstention" conveys a sense of practical magnitude that the absolute effect does not warrant. We have removed this from the abstract and in the introduction we now present the absolute estimate first and describe it as modest in absolute terms, noting the relative magnitude only parenthetically as context for readers unfamiliar with Sweden's high baseline turnout (so we mention actual turnout rates 85–87% for the elections studied in our paper, which would imply $\approx$ 10% reduction in non-voting).

Second, following the reviewer's suggestion, we have reframed the contribution in the abstract and introduction around the persistence of a modest effect over 14 years rather than its magnitude at any single election. As the reviewer notes, this is where the genuine theoretical leverage lies. Habit formation theory does not require a large initial shock—it requires that a shock be large enough to induce participation, after which the self-reinforcing dynamic takes over. A 1.4 percentage-point effect that remains virtually identical from 2010 to 2018 speaks directly to this mechanism, and we now make this logic explicit in the revised manuscript.

Reviewer #3 (Remarks to the Author)

This study investigates an innovative question: can individuals be politically mobilized by natural disasters that they have experienced abroad? The authors analyze the behavior of Swedish survivors of the 2004 Indian Ocean tsunami. Leveraging this case, they disentangle political engagement effects from voting costs induced by infrastructure damage or displacement. The empirics compare Swedish returnees exposed to the disaster with a control group matched on basic demographics across the 1994, 2010, and 2018 elections using informationally rich, register-based individual-level data. The results suggest that individuals personally affected by the tsunami became more politically active upon returning home. A complementary survey identifies enduring dissatisfaction with the incumbent government at the time of the disaster as a key mechanism. Overall, the findings offer insights on voter responses to natural disasters and speak to public debates about civic engagement in the aftermath of large-scale shocks. This is a carefully executed study that features an innovative design and rich data. As such, it has the potential to make valuable contributions to the literature on the politics of natural disasters. I recommend a major revise and resubmit.

Major Comments

> *1. The article's structure and framing require substantial revisions. Currently, the authors devote excessive space to supplementary analyses of the main effect of tsunami exposure on voter turnout, specifically in the subsubsections on treatment intensity, generalizability, and selection into travel. These sections should be considerably condensed and relegated to the appendix. The key threat to identification, the potential violation of the parallel trends assumption due to selection into travel, should be addressed earlier and more systematically, rather than dispersed across multiple robustness checks. Conversely, the mechanism linking tsunami exposure to the experience of failed preparedness investments warrants greater attention. In particular, the authors identify a persistent negative effect of exposure on voting for the then-incumbent Social Democratic Party, yet this finding requires a fuller theoretical and empirical explanation (see below). Overall, the balance of the results section demands major revision.*

Response: We thank the reviewer for this comment, which, together with parallel suggestions from R1 and R2, prompted us to rethink the structure of the Results section from the ground up. Apart from the Background section, which is largely unchanged, the revised Results contain three analytical sections, each with a single, clearly defined purpose.

Main Findings. This section has been rewritten in its entirety and is now organized exclusively around the selection-into-travel threat that the reviewer identifies as the key identification challenge. It opens by naming this threat directly and proceeds through four demanding strategies designed to bound its severity: entropy balancing on pre-tsunami turnout and a rich set of covariates (using the most restrictive sample), an informal sensitivity analysis, entropy-balanced difference-in-differences models, and a within-treatment bereavement dose–response test. All analyses in this section are new. We refer the reviewer to our response to R1, Comment 2, for a detailed description of each strategy and its rationale.

Differential Effects Across Age Cohorts. This is a new section that tests whether the pattern of results is consistent with the theoretical framing we develop in the revised introduction. All reviewers pushed us to clarify and improve the theoretical framing of the paper and we followed your suggestion to ground the paper's contribution in habit formation and political socialization rather than post-traumatic growth (see our response to the first comment by R1 and below). The section shows that the mobilization effect was strongest among those aged 16–25 at the time of the disaster—the impressionable-years cohort—consistent with an account in which the initial accountability-driven mobilization was consolidated into a durable behavioral pattern, especially for those were exposed during a formative period of political development.

Did the Government's Crisis Response Trigger a Lasting Realignment? The survey section has been rewritten to make clear that it provides descriptive evidence, not a causal mechanism test (see our response to R2 on this point). Its purpose is to ask a related but distinct question: did the disaster reshape not only whether survivors vote but whom they vote for? The section walks through three elements—the experience of governmental abandonment, the persistence and specificity of negative crisis-management evaluations, and the partisan consequence—and presents them as descriptive patterns consistent with an accountability response that became crystallized over time.

The sections on generalizability, and selection into travel that the reviewer found excessive have been removed from the main text. The bereavement analysis, previously a standalone robustness check, now serves a specific identification function within Main Findings, and serves no other purpose in our analysis. The core-versus-peripheral voter analysis has been redone to match the

main findings in Table 2 in terms of using the same restrictive sample and analytical techniques (entropy balancing), but have been moved to the Supplementary Information and are now only mentioned in a footnote (see footnote 3, page 10) in the main paper.

2. The theoretical argument to leverage this case to isolate the accountability channel is compelling and innovative. However, it remains unclear why the accountability channel that the authors propose as underpinning the effects on turnout should persist over multiple electoral cycles, despite government turnover. The argument implies that voters continue to hold the government accountable for improving disaster preparedness in the distant future although incumbent politicians and parties have long changed. Thus, given that the authors analyze long-term effects on the 2018 election, they should clarify why persistent turnout should still be understood as an expression of accountability demands when a different party (or different personnel) is in office or when these factors have changed multiple times.
3. The persistent negative effect on Social Democratic Party support in 2022, eighteen years after the disaster, challenges the authors' argument that accountability demands motivate voters. Instead, it points to an enduring shift in party preferences (realignment?) consistent with a socialization effect, particularly given the stronger impact observed among younger voters (along the lines of the impressionable years argument, Brim/Kagan 1980; Franklin 2004). The authors could address this, e.g., by presenting evidence that affected voters continue to be driven by substantive concerns, such as demands for improved preparedness investments, that emerged in the aftermath of the disaster and persist over time. This would be all the more surprising given the absence of a correlation between disaster exposure and mass support for preparedness investment (Bechtel and Mannino 2020). If such an analysis is not possible due to data limitations, the authors should at minimum provide a more detailed discussion of the enduring effects on party preferences and clarify how these long-term dynamics relate to their core argument.

Response: We answer Comments 2 and 3 together because they raise the same fundamental question from two angles: why does the turnout effect persist after government turnover (Comment 2), and why does the partisan shift endure eighteen years later (Comment 3)? These comments were extremely helpful and guided us to think much more clearly about the role of accountability in our argument, and how accountability shocks can have long-lasting effects through habit formation and political socialization during the impressionable years. While we have described the resulting theoretical and analytical changes in detail in our responses to R1 (Comments 1–2) and R2, they were initiated on the impulse of these two comments by R3, and the testable implications they generated are analysed in the new section *Differential Effects Across Age Cohorts*. We summarize the key elements here.

The reviewer is right that ongoing accountability demands cannot, by themselves, explain why the turnout effect persists after government turnover. In the revised manuscript, we address this by distinguishing the initial impulse from the sustaining mechanism. Our theoretical argument is now sequential: the accountability shock triggers an initial mobilization, as survivors who feel abandoned by the government turn out to vote. But accountability need not persist as an ongoing grievance to sustain the turnout effect. The sustaining mechanism is habit formation: citizens who vote in one election are more likely to vote in subsequent ones, and this self-reinforcing dynamic has been shown to operate causally [Gerber et al., 2003, Coppock and Green, 2016, Franklin, 2004]. Research on political socialization further predicts that the habit-formation channel should be strongest among those exposed during their impressionable years [Krosnick and Alwin, 1989, Sears

and Brown, 2013], when political habits are most malleable. This theoretical framework generates a testable implication: if habit formation is the sustaining mechanism, we should observe the largest turnout effects among those who were young adults at the time of the disaster. The new section on age-cohort effects tests exactly this implication using the registry data and finds that the mobilization effect is indeed largest among those aged 16–25 at the time of the disaster, consistent with the theoretical account.

Turning to the partisan shift (Comment 3): we agree with the reviewer's intuition that the persistence of the negative effect on Social Democratic support points toward an enduring re-sorting of partisan attachments rather than ongoing accountability demands. This is precisely the framing we now adopt. The revised survey section is titled "Did the Government's Crisis Response Trigger a Lasting Realignment?" and draws on Key [1955] and Cremaschi et al. [2025] to situate the findings in the literature on critical elections and durable partisan shifts following collective shocks. At the same time, we are now more careful to present the survey evidence as descriptive rather than as a causal mechanism test, given the cross-sectional nature of the data, the limited number of respondents, and the nearly two-decade gap between the event and the survey (see our response to R2 on this point).

Regarding the reviewer's suggestion to test whether survivors continue to demand improved preparedness investments: our survey does include items on crisis preparedness attitudes, and we find that exposed respondents rate the government's handling of the tsunami and of natural disasters significantly worse than controls, but do not differ on evaluations of crisis management in general (Figure 3, Panels C–E; Supplementary Information Table SI.18). This pattern of specificity is informative. It suggests that the grievance is targeted rather than generalized, consistent with a lasting accountability judgment about the 2004 response rather than a broad demand for improved preparedness. This finding is also consistent with Bechtel and Mannino [2023], who find no correlation between disaster exposure and mass support for preparedness investment: our survivors do not demand more preparedness in the abstract, but they do retain a specific, negative evaluation of how the government handled their disaster, and this evaluation coincides with a lasting shift in partisan sympathies.

4. Building on this, the authors should also provide a clearer interpretation of both (i) the persistence of turnout effects (see Theory) and (ii) the increase in the turnout gradient between 2010 and 2018. We know from previous studies that electoral returns to successful disaster relief decay over time. Why and how can accountability failures create growing effects several electoral cycles after the event?

Response: Regarding (i), we hope the reviewer finds that our theoretical reframing and the accompanying analytical changes provide a clearer interpretation of the persistence of the turnout effect (see our response to Comments 2 and 3 above).

Turning to (ii): the revised manuscript substantially refines the analytical strategy, and the apparent increase in the turnout gradient between 2010 and 2018 does not appear in our most demanding specifications. In the restricted sample, which uses entropy balancing on individuals who were eligible to vote in 1994, the estimated effect is nearly identical across both elections (Table 2). The entropy-balanced difference-in-differences results in Supplementary Information Table SI.11 are extremely close to those in Table 2 and likewise show no increase over time.

This pattern suggests that the apparent gradient in the original submission was an artifact of the less demanding matching strategy rather than a substantive feature of the data. By achieving continuous balance on income, education, pre-tsunami turnout in 1994, entropy balancing removes

the compositional imbalances that could generate a spurious upward trend. The stability of the effect across the two elections is, in fact, precisely what habit formation theory predicts: once the initial accountability shock has been consolidated into a durable voting habit, the self-reinforcing dynamic maintains participation at a roughly constant level rather than producing continued growth [Gerber et al., 2003]. A plateau, not an escalation, is the expected signature of habit-driven persistence.

5. In Table 1, the pre-treatment election analysis in Model (1) mechanically excludes individuals who were not yet eligible to vote in 1994, but Models (2) and (3) appear to include this subgroup. This table should instead display the results with a consistent sample, where the post-treatment models also exclude individuals who were not yet eligible to vote, as is reported in Tables S.4/5. Generally, the authors should be significantly more transparent about the samples used in the analyses, which can be easily accommodated in Table footnotes.

Response: The reviewer's concern about sample consistency is well taken. Our revised analytical strategy addresses this directly. The main findings in Table 2 now use the restricted sample throughout: all individuals in the analysis were eligible to vote in 1994, and the control group is entropy-balanced on pre-tsunami covariates including whether they voted in that election. The inconsistency the reviewer identified in the original Table 1 no longer exists.

More broadly, we have taken care to be transparent about the samples used across all analyses. In the revised Methods section, we describe the two sampling strategies we employ and describe the rationale for each: (i) a restricted strategy (limited to individuals eligible to vote in 1994) and an unrestricted strategy (which includes the full electorate at each election). **Table 4** lists which analyses use which sample, and individual table and figure notes refer to this section (e.g., "Uses restricted sample; see Methods"). We are happy to add longer descriptions in each table and figure note if the reviewer considers this necessary.

6. Heterogeneous age effects: The results in Table S.4 and S.5 indicate that the effects of tsunami exposure are substantially larger among younger voters and those who were not yet eligible to vote in 1994. Especially in the absence of additional pre-treatment election years, which is a data limitation beyond the authors' control, this introduces additional concerns about parallel trends. Specifically, demographics for young adults, which appear to be partially assigned based on parents' demographics, may work less well in capturing their life outcomes. Heterogeneous trends among young voters where demographics are less predictive of future life outcomes thus remains a concern that should at least be verbally addressed, although the main effects hold in the restricted sample.

Response: We appreciate this observation, which no longer applies to the revised main findings (since they use the restricted sample), but identifies a limitation of the age-cohort analysis. As we understand the concern: because income and education for the youngest individuals in our sample are imputed from parental records, these covariates may be less predictive of their adult life outcomes than they are for older individuals. If so, entropy balancing on parental characteristics may not fully equalize the relevant pre-treatment differences for younger treated and control individuals, and the larger estimated effects among the young could partly reflect weaker covariate adjustment rather than a genuine impressionable-years pattern.

We want to make two points in response. First, our main finding does not depend on this subgroup. The estimates in Table 2, which use the restricted sample limited to individuals eligible to vote in 1994, are based entirely on voters for whom we observe own income and education rather

than parental proxies. The core result is thus not vulnerable to the imputation concern the reviewer raises.

Second, the age-cohort analysis must use the unrestricted sample by design: restricting to individuals eligible in 1994 would place the youngest individual at age 28 at the time of the disaster, eliminating the impressionable-years cohort entirely. We therefore cannot avoid using parental covariates for this particular analysis. That said, the imputation concern is somewhat mitigated by the fact that parental covariates are used symmetrically for treated and control individuals. Following the reviewer's suggestion, we have added a discussion of this limitation in the manuscript. In the section on the age-cohort effects we note explicitly that the covariates for the youngest cohort are imputed from parental records and that this imputation may provide less precise covariate adjustment for younger individuals, whose adult trajectories are not yet reflected in the data. We caution that the age-cohort effects should be interpreted with this limitation in mind.

Minor Comments

> *1. The authors show that the effects are concentrated among low-income voters and previous non-voters. Both results could be driven by the age dynamic, as young (adult) voters are more likely to earn lower incomes. The interaction with non-voting in 1994 also requires additional explanation. Given that 1994 turnout is the only pre-treatment condition in the main analysis, this analysis seems essentially equivalent to the main effects, where the positive effects on turnout should arise in significant parts from voters who abstained in 1994 and turned out in 2010/2018–at least if they are not purely driven by changing behavior in the control group.*

Response: We agree that this was an important observation. Two changes address it. First, in the revised manuscript we perform these analyses on the restricted sample, in which all individuals were at least 28 years old at the time of the tsunami. The results are therefore not driven by the age dynamic the reviewer identifies. Second, because the findings are largely expected — the effect is strongest among prior non-voters and lower-income individuals — we have moved the analyses to the Supplementary Information and now reference them in a footnote in the Main Findings section. The core/peripheral analyses use the same sample and analytical strategy as Table 2 to ensure comparability, which we felt that this was the appropriate place to mention them. This change also helped streamline the main text, allowing the Results section to focus on the three analytical questions described in our response to R3's Comment 1.

> *2. The authors could elaborate more on the concept of post-traumatic growth and its relation to turnout. The link remains difficult to grasp in the analysis.*

Response: As described in our response to R1.1, we have removed post-traumatic growth as a co-equal mechanism. It is no longer used to explain the turnout results. Instead, we ground the persistence of the turnout effect in habit formation and political socialization during the impressionable years. Post-traumatic growth is now mentioned only briefly in the Discussion, where we note that the positive direction of the effect is consistent with this broader literature. While the post-traumatic growth theory may be a fruitful avenue for future research, we now realize that such a study would require richer psychological data than what we have access to.

> *3. A descriptive table of the composition of bereaved individuals could be added in the Appendix for an improved substantive interpretation of effects in Figure 3. Specifically, were bereaved individuals disproportionately children/parents at the time?*

Response: The identification procedure for the bereaved subsample is described in the Methods section: using Statistics Sweden's multigenerational register, we linked all tsunami-exposed survivors to their extended family networks and cross-referenced these with the official fatality records, identifying 318 bereaved survivors who collectively lost 510 relatives. Because some survivors lost more than one relative, or relatives in multiple categories (e.g., both a parent and a sibling), the relationship categories in Table S.10 are not mutually exclusive. As the reviewer anticipates, bereaved individuals are disproportionately parents who lost children (27.7%) and children who lost parents (41.5%). We note in the main text that bereavement clusters within families, often families with young children who were not yet eligible to vote in 1994, which is why the bereavement analysis uses the unrestricted sample. Following the reviewer's suggestion, we have added a descriptive table in the Supplementary Information (Table S.10) that breaks down the bereaved group by relationship to the deceased and the groups age and sex.

Regarding potential complications: if the bereaved group were much larger and composed predominantly of children who lost parents, bereavement could in principle confound the age-cohort effects. But the group is small (318 individuals), so this concern does not apply. A related question is whether the bereaved are systematically younger than the non-bereaved exposed, which could inflate the bereavement coefficient in Table 3. The analysis controls for age and age squared, which addresses this concern directly.

> *4. The analyses conducted in the "Generalizability" section do not necessarily address the generalizability of the results but rather perform extensions.*

Response: We agree. As described in our response to R3's minor comment 1, we have moved these analyses to the Supplementary Information, and they are referenced in a footnote in the Main Findings section. We no longer frame them as addressing generalizability; instead, they are presented as evidence that the mobilization effect is larger among prior non-voters and lower-income individuals (i.e. 'peripheral' voters).

References

Michael M. Bechtel and Massimo Mannino. Ready when the big one comes? Natural disasters and mass support for preparedness investment. *Political Behavior*, 45(3):1045–1070, 2023.

Julia Bergquist. In Ulrika Andersson, Patrik Öhberg, Anders Carlander, Johan Martinsson, and Nora Theorin, editors, *Den nationella SOM-undersökningen 2022. In* Ovisshetens tid, pages 501–510. SOM-institutet, Göteborgs universitet, 2023.

Carlos Cinelli and Chad Hazlett. Making sense of sensitivity: Extending omitted variable bias. *Journal of the Royal Statistical Society: Series B (Statistical Methodology)*, 82(1):39–67, 2020. doi: 10.1111/rssb.12348.

Alexander Coppock and Donald P. Green. Is voting habit forming? new evidence from experiments and regression discontinuities. *American Journal of Political Science*, 60(4):1044–1062, 2016. doi: 10.1111/ajps.12210.

Simone Cremaschi, Nicola Bariletto, and Catherine E. De Vries. Without roots: The political consequences of collective economic shocks. *American Political Science Review*, 2025. doi: 10.1017/S0003055424001522.

Mark N Franklin. *Voter turnout and the dynamics of electoral competition in established democracies since 1945*. Cambridge University Press, 2004.

Alan S. Gerber, Donald P. Green, and Ron Shachar. Voting may be habit-forming: Evidence from a randomized field experiment. *American Journal of Political Science*, 47(3):540–550, 2003. doi: 10.1111/1540-5907.00038.

Jens Hainmueller. Entropy balancing for causal effects: A multivariate reweighting method to produce balanced samples in observational studies. *Political Analysis*, 20(1):25–46, 2012. doi: 10.1093/pan/mpr025.

V. O. Key. A theory of critical elections. *The Journal of Politics*, 17(1):3–18, 1955. doi: 10.2307/2126401.

Jon A. Krosnick and Duane F. Alwin. Aging and susceptibility to attitude change. *Journal of Personality and Social Psychology*, 57(3):416–425, 1989. doi: 10.1037/0022-3514.57.3.416.

Karl Oskar Lindgren, Sven Oskarsson, and Mikael Persson. Enhancing electoral equality: Can education compensate for family background differences in voting participation? *American Political Science Review*, 113(1):108–22, 2019.

David O. Sears and Christia Brown. In Leonie Huddy, David O. Sears, and Jack S. Levy, editors, *Childhood and Adult Political Development. In* The Oxford Handbook of Political Psychology, pages 59–95. Oxford University Press, 2nd edition, 2013.

Response to Reviewers

Manuscript NCOMMS-25-67162A: *Long-term electoral participation among Swedish survivors of the 2004 Indian Ocean tsunami*
Journal: *Nature Communications*

We thank the reviewers for their careful reading of the revised manuscript and for their positive assessment. Reviewers 1 and 4 raised no further requests. Below we respond point by point to the remaining comments from Reviewers 2 and 3.

Reviewer #1

The authors have done much to strengthen the manuscript. I remain somewhat skeptical about the validity of the research design due to multiple threats to causal inference, and the proposed mechanism remains somewhat underdeveloped, which in turns means that the overall contribution to theory is modest. That said, I believe the manuscript merits publication given its innovative use of a unique event analysis to evaluate the effect of natural disasters on political participation.

Response: We thank the reviewer for the assessment. No further changes were requested.

Reviewer #2

Thank you for the opportunity to review the revised manuscript. The paper has improved substantially. The authors have strengthened the empirical strategy, especially by replacing the original matching approach with entropy balancing and by adding complementary analyses that more directly address selection into exposure. The theoretical framing is also clearer, and the decision to treat the 2023 survey evidence as descriptive rather than causal is appropriate.

My remaining concern is about how the authors characterize what the design identifies. The manuscript still suggests that the setting allows them to isolate a "motivational channel," because survivors returned to intact institutions and did not face the resource constraints common in many disaster settings. This claim appears already in the abstract, and I think it remains somewhat too strong. Disaster exposure can generate psychological, social, and economic consequences that are not directly observed in the data and that do not map neatly onto a motivation-versus-resources distinction. These pathways cannot be cleanly disentangled in the present design.

I therefore encourage the authors to further temper this language and to frame the results as consistent with motivational responses, rather than as isolating a specific motivational channel. This is a matter of interpretation rather than a fundamental problem with the analysis. Consequently, I recommend publication subject to minor revisions.

Response: We agree and have tempered the language throughout. The abstract, Introduction, Background and Discussion no longer state that the design isolates a motivational channel. We now say that the setting removes the cost-of-voting increases that confound most disaster studies, and

that the results are consistent with a motivational response, while noting in the Discussion that we cannot rule out indirect channels such as long-term mental health impacts, family disruption, or economic strain.

Reviewer #3

The authors successfully responded to the comments and the paper makes a valuable contribution to the literature on the political consequences of natural disasters. I recommend acceptance conditional on several minor points. The revisions in response to these comments can in my view be evaluated in-house.

1. The additional difference-in-differences analysis discussed on p. 9-10 seems redundant given the inclusion of turnout in 1994 as a control variable. The authors write that the results presented in S.11 are comparable, and although the estimates are virtually identical the standard errors are slightly larger. This can be solved by dropping the additional analysis from the main body. In contrast, the additional sensitivity check bolsters the credibility of the estimated effects.

Response: We agree and have removed the difference-in-differences analysis from the main text. Because earlier reviewer responses referred to these models, and in the interest of transparency, we report them in full in Supplementary Table 9 and retain a single sentence in the Main Findings section noting that they give nearly identical estimates. The sensitivity analysis remains in the main text.

2. Figure 3, Panels A-B: The authors write that "exposed respondents report receiving almost no support from public authorities in the aftermath of the disaster" and that "[t]hose who did receive assistance overwhelmingly judged it inadequate". I wonder whether this is actually consistent with the results in Panel A which indicate that the average rating of support from public authorities among both exposed and severely exposed respondents is at around 2, which is the midpoint of the scale (which ranges from 0 to 4).

Response: The reviewer is right that our wording overstated Panel A. We have rewritten the passage. The "almost no support" finding comes from Panel B, which shows that most exposed respondents, and close to half of the severely exposed, had no contact with public authorities at all. Among those who did have contact, the mean rating is close to the midpoint of the scale, which we now describe as "no better than neutral," and we note that it is well below the ratings given to family and friends.

3. All Figures: please ensure informative axis titles. Examples include: Figure 2 is missing information about the unit in which the estimated effects are measured. The notes say they are "The Effect of Tsunami Exposure on Voter Turnout", so presumably these are changes in percentage points. This should be added as axis title information. Another example is Panel F, Figure 3 which information about the party codes is missing. Ideally, the party names are spelled out in the plot or at least informative labels should be used and explained in the note.

Response: Done. All figures now carry axis titles that state the unit (percentage points for the

effect estimates). In Figure 3, Panel F, the party labels are the standard Swedish abbreviations. The figure legend now spells out each party's full name and party family.

> *4. Repetitive language: The authors conclude multiple sections with the phrase "Taken together", which makes the prose unnecessarily repetitive. Consider varying these transitions.*

Response: Done. The phrase now appears only once.

Reviewer #4

> *I co-reviewed this manuscript with one of the reviewers who provided the listed reports. This is part of the Nature Communications initiative to facilitate training in peer review and to provide appropriate recognition for Early Career Researchers who co-review manuscripts.*

Response: We thank the co-reviewer for their contribution. No further changes were requested.
